# Cultivation of Fungal Endophytes with Tissue Culture Grapevine Seedlings Reprograms Metabolism by Triggering Defence Responses

**DOI:** 10.3390/metabo14080402

**Published:** 2024-07-24

**Authors:** Xiaoxia Pan, Huizhi Liu, Yiqian Li, Lirong Guo, Yunuo Zhang, Youyong Zhu, Mingzhi Yang

**Affiliations:** 1 Key Laboratory of Chemistry in Ethnic Medicinal Resources, State Ethnic Affairs Commission & Ministry of Education, School of Ethnic Medicine, Yunnan Minzu University, Kunming 650504, China; pan901805@126.com (X.P.);; 2 School of Ecology and Environmental Science, Yunnan University, Kunming 650504, China; 3 Key Laboratory for Agro-Biodiversity and Pest Control of Ministry of Education, Yunnan Agricultural University, Kunming 650201, China

**Keywords:** grapevine, fungal endophytes, *Epicoccum layuense* R2-21, *Alternaria alternata* XHYN2, defense response, transcriptomic analysis, gene expression reprogramming

## Abstract

In this study, the transcriptome profiles of tissue–cultured grapevine (*Vitis vinifera* L. × *Vitis labrusca* L.: Rose Honey) seedlings inoculated with fungal endophytes *Epicoccum layuense* R2-21 (Epi R2-21) and *Alternaria alternata* XHYN2 (Alt XHYN2), were analyzed at three different time points (6 h, 6 d, and 15 d). A total of 4783 differentially expressed genes (DEGs) was found, of which 1853 (6 h), 3878 (6 d), and 4732 (15 d) were differentially expressed relative to those of the control in endophyte Epi R2-21 treatments, while a total of 5898 DEGs, of which 2726 (6 h), 4610 (6 d), and 3938 (15 d) were differentially expressed in endophyte Alt XHYN2 treatments. DEGs enriched in secondary metabolic pathways, plant–pathogen interaction, and hormone signalling were further analysed. The upregulated DEGs in the Epi R2-21 and Alt XHYN2 treatments, both enriched in the Kyoto Encyclopedia of Genes and Genomes (KEGG), were mainly involved in flavonoid biosynthesis, phenylpropanoid biosynthesis, stilbenoid, diarylheptanoid and gingerol biosynthesis, phenylalanine metabolism, and circadian rhythms–plant and plant–pathogen interactions, similar to the trend observed in our previous study conducted on the cultivar ‘Cabernet Sauvignon’ (*Vitis vinifera* L.). Taken together with the results obtained from the cultivar ‘Cabernet Sauvignon’, it was found that tissue-cultured seedlings of the cultivar ‘Rose Honey’ induced a stronger defence response to fungal endophyte infection than that of the cultivar ‘Cabernet Sauvignon’, and inoculation with the endophyte Alt XHYN2 triggered a stronger response than inoculation with the endophyte Epi R2-21. In addition, the protein–protein interaction (PPI) network revealed that the genes *VIT_16s0100g00910*, encoding CHS, and *VIT_11s0065g00350*, encoding CYP73A, were involved in secondary metabolism and thus mediated in the resistance mechanism of grapevine on both the cultivars. The results showed that inoculation with the endophytes Epi R2-21 and Alt XHYN2 had a great ability to induce defence responses and reprogram the gene expression profiles in different grapevine cultivars, which deepens our knowledge of the interaction between fungal endophytes and grapevine and gives hints for grape quality management in viticulture using candidate fungal endophytes.

## 1. Introduction

Endophytes are special microorganisms that live within plants with no obvious symptoms and establish interactions with plants ranging from mutualism to pathogenicity [1]. Some researchers have tended to define the term ‘endophytes’ as referring only to the habitat of the organisms and not to their functions, i.e., all microorganisms that colonize in the internal tissues of plants for all or part of their life [2]. Endophytes, as important cooperative partners of the host plants during their growth and development, have a major impact on the physiological metabolism of the host plant, which helps or stimulates the host plant to defence stresses [3]. Many studies have shown that endophytes are repositories of bioactive metabolites with pharmacological activities, such as antimicrobial, antitumour, antibiotic, antioxidant, and immunological agents [4]. The exploration of them has become a hot spot as the interaction between endophytes and the host plant is gradually being uncovered. Therefore, it is of great value to elucidate the interactive mechanism between plants and endophytes in order to explore and unlock the potential application of endophytes.

Endophytes can colonize the host plant in a manner similar to that of pathogen invasion [5], and host plants can synthesize a variety of stress–induced metabolites to defend themselves against stress. In recent years, the theory of balanced antagonism has emerged as a more accurate explanation of the interaction between the host plant and endophytes [6]. According to this theory, endophytes possess virulence factors that confer self−resistance to toxic metabolites of the host [7,8]. Asymptomatic colonization is a balance of antagonisms between plants and endophytes. When endophytic virulence and plant defence are in equilibrium, the endophyte manages to grow in the host plant and remains asymptomatic or avirulent [7,9]. However, the plant–endophyte interaction is not simply a balance between virulence and defence, and the regulatory network or mechanisms are much more complex [10]. 

Grapes (*Vitis vinifera* L.), as the most widely cultivated fruit in the world for the production of table fruit, dried fruit, juice, and wine, harbour diverse microorganisms, including fungal endophytes, some of which contribute to the terroir that defines the qualities and characteristics of grapes and wines [11,12]. With a full understanding of how grape metabolism responds to fungal endophyte colonization, it is important to adapt agricultural practices in viticulture and then improve the resulting products, such as wine. In recent years, the application of omics technology has gradually revealed the interactions between grapevines and endophytes or pathogens. Zhang et al. [13] investigated the interaction of grapevine green shoots with the fungal pathogen *Lasiodiplodia theobromae* at three different time points and integrated differential expression genes (DEGs) involved in biotic stress responses, such as plant–pathogen interaction, hormone signalling, and phenylpropanoid biosynthesis pathways. After inoculation with downy mildew *Plasmopara viticola* at four different time points, genes related to the synthesis of defense–related metabolites, such as phenylpropanoid, stilbenoid, and flavonoid biosynthesis genes, were activated in ‘Shuanghong’ grapevine leaves during the early stages of infection [14]. Similar results have also been reported on the mechanisms underlying the interaction between grapevines and the pathogenic fungi *Plasmopara viticola*, identifying resistance genes and pathways involved in signalling, as well as an induction of the basal immune response and the terpene metabolism [15]. Transcriptomic and metabolic analyses revealed the interaction between the grape berries and the *Botrytis cinerea* pathogen, and DEGs related to the monolignol, flavonoid, and stilbenoid biosynthetic pathways were significantly induced in the berries [16]. All of the above results were mainly related to grapevine–pathogen interactions; whether the colonization of fungal endophytes in grapevine without causing obvious symptoms leads to reprogramming of the host plant metabolism triggered in response to the defence mechanism has been scarcely reported. In our previous study, we analysed the different responses after 6 h, 6 d, and 15 d of inoculation with fungal endophytes *Epicoccum layuense* R2-21 (Epi R2-21) and *Alternaria alternata* XHYN2 (Alt XHYN2), at the transcriptomic level of tissue–cultured grapevine seedlings of the cultivar ‘Cabernet Sauvignon’ (*Vitis vinifera* L. cv., the wine grape cultivar cultivated worldwide) [17], and the results showed that grapevine genes were greatly regulated in response to Epi R2-21 and Alt XHYN2 inoculation, resulting in the reprogramming of genes related to the secondary metabolite profile.

However, it is unclear whether these responses represent cultivar– or genus–specific differences in the cultivar ‘Cabernet Sauvignon’ or common effects in different grapevine cultivars. To validate these effects, in the present work, we further investigated the effects of the same fungal endophytes on tissue–cultured grapevine seedlings of the cultivar ‘Rose Honey’ (*Vitis vinifera* L. × *Vitis labrusca* L., the wine grape cultivar grown in Yunnan Province, China), and compared the responses at the transcriptomic level in seedlings with the fungal endophytes Epi R2-21 and Alt XHYN2 to study serial changes in the gene profiles altered after 6 h, 6 d, and 15 d inoculation, providing insights into the interactions between fungal endophyte and grapevine at the early and later stages.

## 2. Materials and Methods

### 2.1. Preparation of Fungal Endophytes and Tissue–Cultured Grapevine Seedlings

The fungal endophytes Epi R2-21 and Alt XHYN2 were isolated from grapevine leaves of the cultivars ‘Cabernet Sauvignon’ and ‘Xiahei’, respectively (Figure S1A–D). After being cultured on potato dextrose agar (PDA: potato 200 g/L, dextrose 20 g/L, agar 15 g/L) medium for 7 d, the fungal growth masses were harvested and suspended in 0.9% normal saline to a final concentration of 2.5 g/L, respectively.

Tissue–cultured seedlings of *Vitis vinifera* L. × *Vitis labrusca* L. ‘Rose Honey’ (single–bud clones) were grown in Murashige and Skoog (MS) medium [18] in the greenhouse (12 h light and 12 h dark at 25 °C) for 2 months with 7–10 expanded leaves prepared for the study. The MS medium contained 3% sucrose (*m*/*v*) and 0.75% agar, and it was supplemented with vitamins (myo–inositol 100 mg/L, nicotinic acid 1 mg/L, thiamine HCl 1 mg/L, pyridoxine HCl 1 mg/L, D–calcium pantothenate 1 mg/L, and biotin 0.01 mg/L).

### 2.2. Fungal Endophytes Inoculation and Fungal Endophyte Isolation Rates Determination

Suspensions of the fungal growth masses of Epi R2-21 and Alt XHYN2 were smear inoculated onto tissue–cultured seedlings, while seedlings without fungal inoculation were used as controls. After 6 h, 6 d, and 15 d inoculation, all seedling samples were collected, dissected, mixed, and rapidly stored at −80 °C for RNA extraction. After 15 d of inoculation with the endophytes Epi R2-21 and Alt XHYN2, respectively, leaves were harvested to determine the isolation rates of fungal endophytes using the tissue patch method [19]. The isolation rates were calculated as the percentage of emerging fungal colonies per leaf patch and used to describe the symbiotic efficiency of the fungal endophytes, and the fungal colonies were identified using internal transcribed spacer (ITS) DNA sequences with primer pairs ITS4 and ITS5 [20]. The nucleotide sequences of the endophytes Epi R2-21 and Alt XHYN2 were deposited in GenBank under the accession numbers OR826156 and OR826157. Samples were defined as R2-21_6 h, R2-21_6 d, R2-21_15 d (Epi R2-21 group, tissue–cultured grapevine after co–culture with Epi R2-21 for 6 h, 6 d, 15 d, respectively); XHYN2_6 h, XHYN2_6 d, XHYN2_15 d (Alt XHYN2 group, tissue–cultured grapevine after co–culture with Alt XHYN2 for 6 h, 6 d, 15 d, respectively); and Con_6 h, Con_6 d, Con_15 d (control group). Three biological replicates were carried out with 10 plants per replicate.

### 2.3. RNA Sequencing, cDNA Library Construction and qRT–PCR

Total RNA was extracted using Trizol reagent (Invitrogen, Carlsbad, CA, USA) and purified with DNase I to remove the genomic DNA (Takara, Tokyo, Japan). The RNA samples were analysed with a NanoDrop 2000 spectrophotometer (Thermo, Waltham, MA, USA), and the RNA–seq transcriptome library was constructed using the TruSeq^TM^ RNA Sample Preparation Kit from Illumina (San Diego, CA, USA) at the Major Company (Shanghai, China). The mRNA with poly (A) was isolated with oligo (dT) beads, and then double–stranded cDNA was synthesized and repaired. Raw sequencing data were processed by trimming adapter sequences, excess poly–N, reads of <30 bp in length, empty reads, and reads with a quality score of less than 20 using SeqPrep (https://github.com/jstjohn/SeqPrep) (accessed on 16 January 2021) and Sickle (https://github.com/najoshi/sickle) (accessed on 16 January 2021) with default parameters. Paired–end reads were mapped to the *Vitis vinifera* reference genome (http://plants.ensembl.org/Vitis_vinifera/Info/Index) (accessed on 16 January 2021) using Hisat2 version 2.1.0 (http://ccb.jhu.edu/software/hisat2/index.shtml) (accessed on 16 January 2021). 

To verify the quality of the RNA–Seq data, seven genes were selected for validation by quantitative real–time PCR (qRT–PCR). Total RNA was reverse transcribed into cDNA using the HiScript Reverse Transcription Supermix for RT–qPCR (Vazyme Biotech, Nanjing, China), and 0.6 µg of RNA was used for reverse transcription in a 20 µL reaction volume. The PCR was performed using the SYBR Green’s method on an ABI7500 fluorescent quantitative PCR machine (Applied Biosystems, Waltham, MA, USA) in 96–well plates, and three biological replicates were conducted in each sample. The reaction mixture (final volume of 20 µL) contained 2 µL cDNA template (1 ng/µL), 16.4 µL ChamQ SYBR Color qPCR Master Mix (Vazyme Biotech, Nanjing, China), 0.8 µL forward primer (4 µM), and 0.8 µL reverse primer (4 µM). Primers are listed in Table S1. The reaction conditions were as follows: 95 °C for 5 min, followed by 40 cycles of denaturation at 95 °C for 5 s and annealing at 55 °C for 30 s, and extension at 72 °C for 40 s. Transcript levels were normalized to the reference gene *EF1*, and the relative quantification of gene expression was computed using the 2^−ΔΔCt^ method [21].

### 2.4. Identification of Differentially Expressed Genes (DEGs) and Analysis Enrichment Pathways

Differential gene expression was investigated using the Fragments per Kilobase of Transcript per Million Mapped Reads (FPKM) using RNA–Seq by expectation–maximization (RSEM, http://deweylab.biostat.wisc.edu/rsem/) (accessed on 17 January 2021) [22]. Differential expression analysis was performed on the identified DEGs using the DESeq2 [23]. The resulting *p*-values were adjusted using the Benjamini and Hochberg approach to control for the false discovery rate. The DEGs were evaluated using *p*-adjust < 0.05 and |log_2_FC| ≥ 1 as the criterion. The DEGs were subjected to Gene Ontology (GO) functional enrichment and Kyoto Encyclopedia of Genes and Genomes (KEGG) (http://www.genome.jp/kegg/) (accessed on 17 January 2021) pathway enrichment analysis to characterize biological functions by Goatools (https://github.com/tanghaibao/Goatools) (accessed on 17 January 2021).

K–mean clustering was conducted on log_2_–transformed FPKM values by Euclidean correlation as a similarity metric to visualize genes with similar expression patterns and explore their functions. Time–course DEG analysis was carried out based on the microarray Significant Profiles (maSigPro) (version 1.56.0) [24]. 

### 2.5. Correlation Networks Analysis for Key Gene Screening

To identify genes that play a key role in endophyte defence and metabolite production, the protein–protein interaction (PPI) network was constructed according to the Search Tool for the Retrieval of Interacting Genes (STRING, http://string-db.org/) database (accessed on 25 October 2023), and key gene clusters and network nodes were further analysed using Cytoscape software (version 3.4.1, http://cytoscape.org/) (accessed on 25 October 2023). The confidence score cut–off used for interactions was 0.4. The colour and size of the nodes were determined based on the betweenness centrality and the degree, respectively.

## 3. Results

The tissue–cultured seedlings maintained good physiological conditions after co–culture with Epi R2-21 and Alt XHYN2 (Figure S1E–G). However, some of the leaves inoculated with Alt XHYN2 were slightly yellowed with some rot. This was most likely due to the fact that Alt XHYN2 belongs to the fungal pathogen Alternaria, which triggers a stronger defence response in the host plant. Inoculation of the endophytes R2-21 and Alt XHYN2 could successfully infect and colonize in tissue–cultured seedlings ‘Rose Honey’, and the isolation rates of Epi R2-21 and Alt XHYN2 were 34% and 32%, respectively.

### 3.1. RNA–Seq Analysis of Tissue–Cultured Seedlings Treated with Endophyte Inoculation

**Figure 1:**
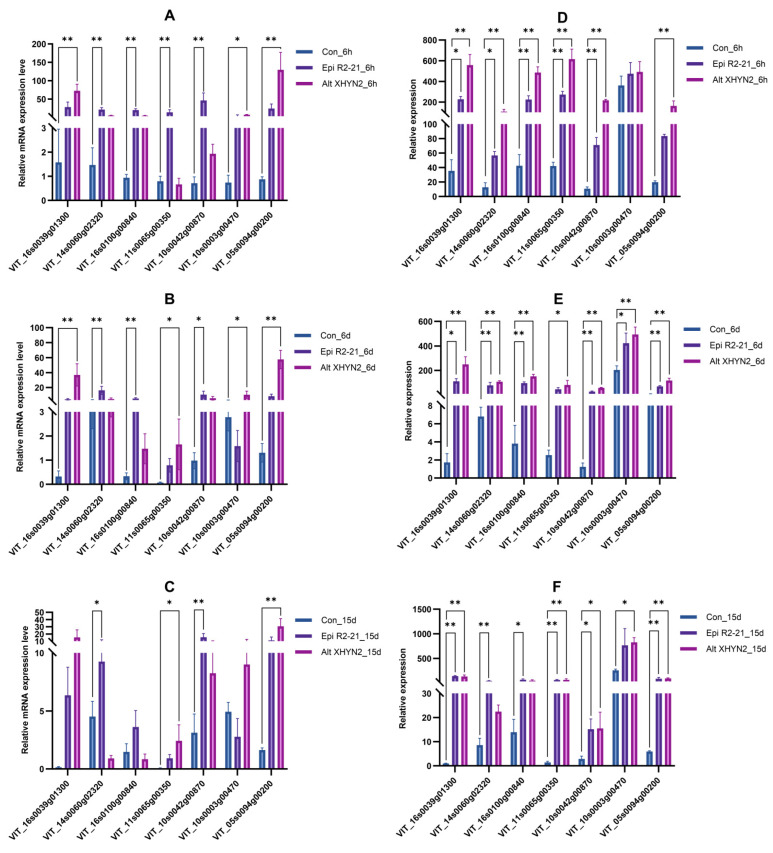
qRT–PCR validation of the expression profiles of seven genes in tissue–cultured seedlings of ‘Rose Honey’ after inoculation with Epi R2-21 and Alt XHYN2. (**A**–**C**) represent the qRT–PCR values after 6 h, 6 d, and 15 d of inoculation, respectively. (**D**–**F**) represent the transcriptome values after 6 h, 6 d, and 15 d inoculation, respectively. Values are mean ± standard deviation (SD) of three individual replicates (*n* = 3). Data were statistically analysed using Tukey’s HSD tests (* *p* < 0.05, ** *p* < 0.01).

Approximately 49.1–60.3 million raw reads were obtained from all control and treatment groups, while 48.8–59.7 million clean reads were obtained after rigorous quality checks and data filtering. GC percentages for these clean reads ranged from 45.76% to 46.84%. The Q20 and Q30 percentages (quality greater than 20 and 30, respectively, for each base) of all libraries were >95% (Table S2), indicating that the data generated by sequencing are of high quality. To analyse the transcriptome profile, clean reads were mapped to the grapevine reference genome (accession numbers PN40024.v4, http://plants.ensembl.org/Vitis_vinifera/Info/Index) (accessed on 16 January 2021). The mapping ratios for all control and treatment groups were from 88.22% to 93.25%, and between 84.59% and 90.62% of genes were uniquely mapped to a single location. In addition, the proportion of exons was the highest among all libraries, ranging from 93.25% to 95.00%, the proportion of introns ranged from 3.30% to 4.91%, and the proportion of intergenic regions was the lowest, ranging from 1.53% to 1.95% (Table S2). To validate the quality of the RNA–Seq data, seven DEGs that were enriched in primary and secondary metabolic pathways in each sample were selected for qRT–PCR analysis. The RT–qPCR results were consistent with those obtained from the RNA-Seq, except for the gene *VIT_11s0065g00350* in sample XHYN2_6h, the gene *VIT_10s0003g00470* in samples R2-21_6d and R2-21_15d, the gene *VIT_14s0060g02320* in sample XHYN2_15d, and the gene *VIT_16s0100g00840* in sample XHYN2_15d (Figure 1), indicating the reliability of the transcriptomic profiling data.

Replicates could be grouped into principal component analysis (PCA) plots based on transcript profiles in tissue–cultured seedlings (Figure S2A). Sample groups Con_6h, Con_6d, and Con_15d showed a shorter distance than other groups. In contrast, the two groups R2-21_6h and XHYN2_6h and the four groups R2-21_6d, XHYN2_6d, R2-21_15d, and XHYN2_15d showed shorter distances to each other. These data formed a continuous and distinct set, highlighting that inoculation with Epi R2-21 and Alt XHYN2 induced significant gene changes over time in tissue–cultured seedlings.

### 3.2. Identification of DEGs 

In the present study, a total of 4783 and 5898 genes were found to be differentially expressed in tissue–cultured seedlings after inoculation with Epi R2-21 and Alt XHYN2 for 6 h, 6 d, and 15 d, respectively. In tissue–cultured seedlings after inoculation with Epi R2-21 for 6 h, 6 d, and 15 d, 1853 (1125 up– and 728 downregulated), 3878 (2333 up– and 1545 downregulated), and 4732 (2588 up– and 2144 downregulated) DEGs were identified, respectively (Figure S2B). While in Alt XHYN2 treatments, 2726 (1451 up– and 1275 downregulated), 4610 (2992 up– and 1618 downregulated), and 3938 (2101 up– and 1837 downregulated) DEGs were identified after 6 h, 6 d, and 15 d inoculation, respectively (Figure S2B). A unique set of DEGs (59, 64, 323 DEGs) was upregulated after inoculation with Epi R2-21 for 6 h, 6 d, and 15 d, respectively, and a unique set of DEGs (152, 598, 124) was upregulated after inoculation with Alt XHYN2 for 6 h, 6 d, and 15 d, respectively, while 425 common DEGs were upregulated at all three time points (Figure S2C). In addition, a unique set of DEGs (38, 142, 559) was downregulated after inoculation with Epi R2-21 for 6 h, 6 d, and 15 d, respectively, and a unique set of DEGs (241, 464, 283) was downregulated after inoculation with Alt XHYN2 for 6 h, 6 d, and 15 d, respectively, while 149 common DEGs were downregulated at all three time points (Figure S2D).

### 3.3. Cluster Analysis, Function Annotation, and Expression Patterns of the DEGs

**Figure 2:**
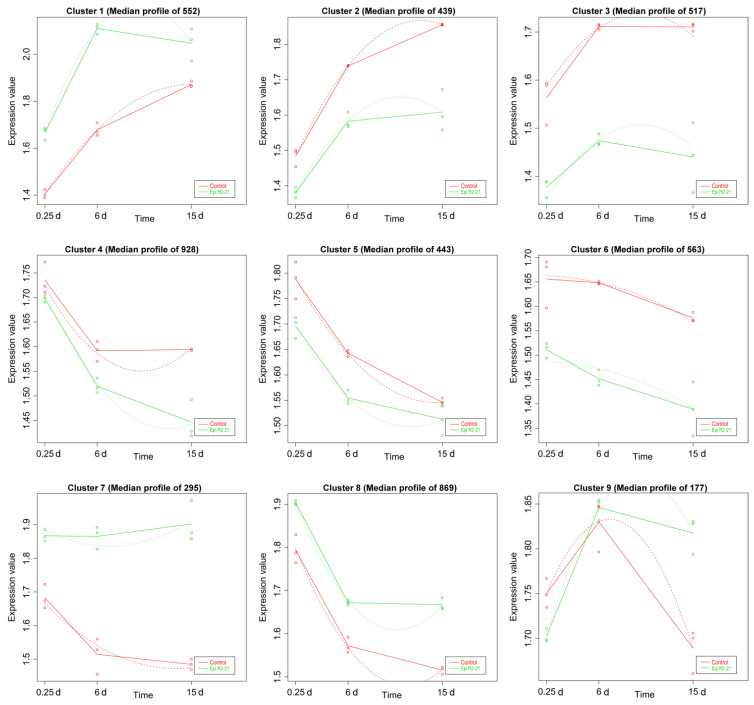
Time–course DEGs analysis of tissue–cultured seedlings after inoculation with Epi R2-21. Median profile means the average expression values of the gene clusters from all samples. The dots show the actual average expression values for each sample; red dots or lines represent the control group, and green dots or lines represent the Epi R2-21 group, respectively.

**Figure 3:**
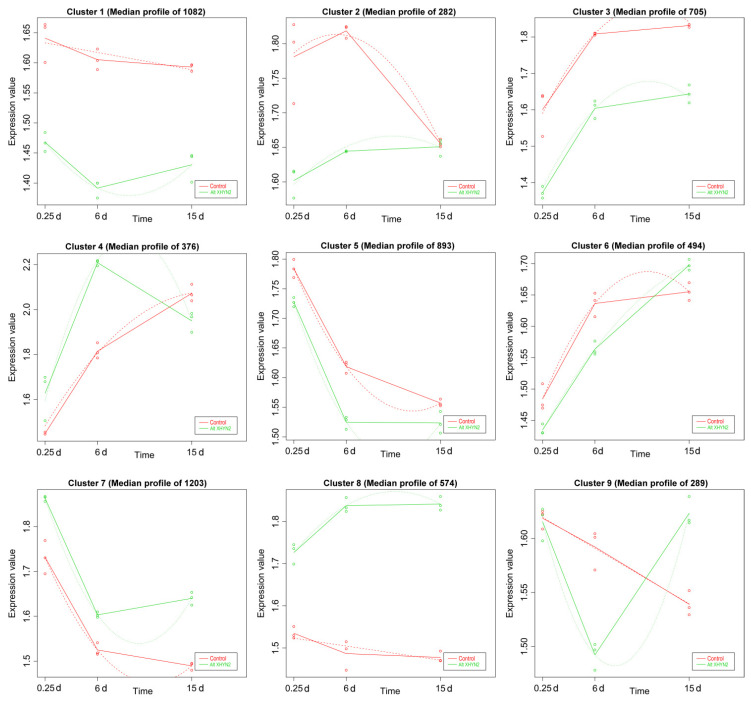
Time–course DEGs analysis of tissue–cultured seedlings after inoculation with Alt XHYN2. Median profile means the average expression values of the gene clusters from all samples. The dots show the actual average expression values for each sample; red dots or lines represent the control group, and green dots or lines represent the Alt XHYN2 group, respectively.

K–mean analysis was performed on the expression levels of the DEGs. As a result, nine expression patterns were identified in the Epi R2-21 and Alt XHYN2 treatments, respectively, and the number of genes varied from 177 to 928 and from 282 to 1203 in each cluster (Figure 2 and Figure 3). Compared to the controls, the gene expression of the Epi R2-21 treatments varied within a certain pattern in each cluster: in clusters 1, 7, 8, the expression of DEGs was consistently upregulated as compared to the control. The DEGs were mainly related to the plant–pathogen interaction, amino sugar and nucleotide sugar metabolism, phenylalanine metabolism, stilbenoid, diarylheptanoid and gingerol biosynthesis, flavonoid biosynthesis, citrate cycle, protein processing in the endoplasmic reticulum, etc. (Table 1). In clusters 2, 3, 4, 5, and 6, the expression of DEGs was consistently downregulated as compared to the control. The DEGs were mainly related to carbon fixation in photosynthetic organisms, photosynthesis–antenna proteins, photosynthesis, ribosome, spliceosome, etc. While in cluster 9, the gene expression decreased from 6 h to 5 d, and increased after 5 d, but genes were not significantly enriched in any pathway in the Epi R2-21 treatments (Figure 2; Table 1). In Alt XHYN2 treatments, the DEGs in clusters 7 and 8 were consistently upregulated as compared to control, mainly related to the citrate cycle, protein processing in the endoplasmic reticulum, glycolysis/gluconeogenesis, flavonoid biosynthesis, circadian rhythm–plant, phenylalanine metabolism, stilbenoid, diarylheptanoid and gingerol biosynthesis, etc. In clusters 1, 2, 3, and 5, the DEGs were consistently downregulated as compared to the control, mainly related to porphyrin and chlorophyll metabolism, ribosome, spliceosome, photosynthesis–antenna proteins, photosynthesis, etc. In cluster 4, the expression tended to increase from 6 h to 13 d but decreased after 13 d; the DEGs were mainly related to plant–pathogen interaction. In cluster 6, the expression tended to decrease from 6 h to 12 d but increased after 12 d; and in cluster 9, the DEGs were initially downregulated but then upregulated as compared to the control. The DEGs in these two clusters were mainly related to protein processing in the endoplasmic reticulum, spliceosome, and ubiquitin–mediated proteolysis (Figure 3; Table 1).

**Figure 4:**
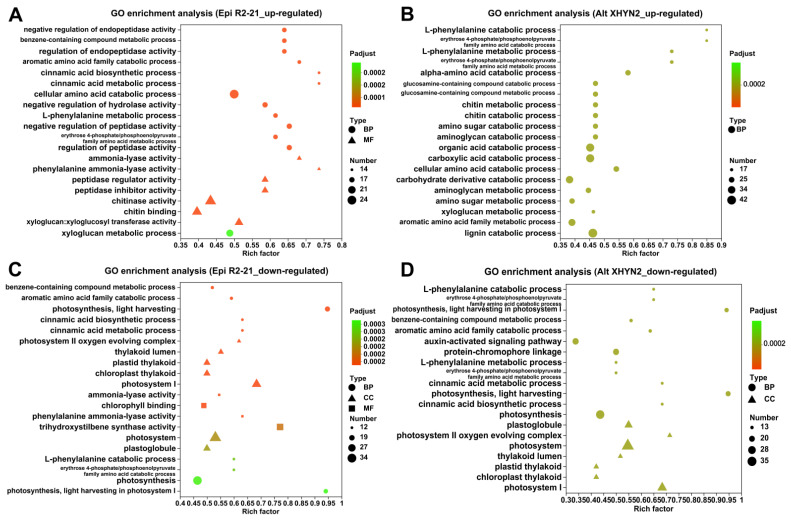
Scatter plot of the GO terms for the up– and downregulated DEGs in tissue–cultured seedlings after inoculation with Epi R2-21 and Alt XHYN2. (**A**) Upregulated DEGs inoculated with Epi R2-21; (**B**) upregulated DEGs inoculated with Alt XHYN2; (**C**) downregulated DEGs inoculated with Epi R2-21. (**D**) Downregulated DEGs inoculated with Alt XHYN2. BP: biological process; MF: molecular function; CC: cell component.

We further analysed the functions of the up– and downregulated DEGs to assess their underlying biological significance through GO enrichment analysis (Figure 4). The upregulated DEGs of Epi R2-21 with enriched GO terms were mainly enriched in the negative regulation of endopeptidase activity, metabolic process of benzene–containing compounds, and regulation of endopeptidase activity (Figure 4A); the upregulated DEGs in Alt XHYN2 treatments were significantly enriched in the L–phenylalanine catabolic process, erythrose 4–phosphate/phosphoenolpyruvate family amino acid catabolic process, L–phenylalanine metabolic process, and erythrose 4–phosphate/phosphoenolpyruvate family amino acid metabolic process (Figure 4B). While the downregulated DEGs in Epi R2-21 treatments were enriched in the benzene–containing compound metabolic process, aromatic amino acid family catabolic process, photosynthesis, and light harvesting (Figure 4C); and the downregulated DEGs of Alt XHYN2 were enriched in the L–phenylalanine catabolic process, erythrose 4–phosphate/phosphoenolpyruvate family amino acid catabolic process, photosynthesis, and light harvesting in photosystem I (Figure 4D).

**Figure 5:**
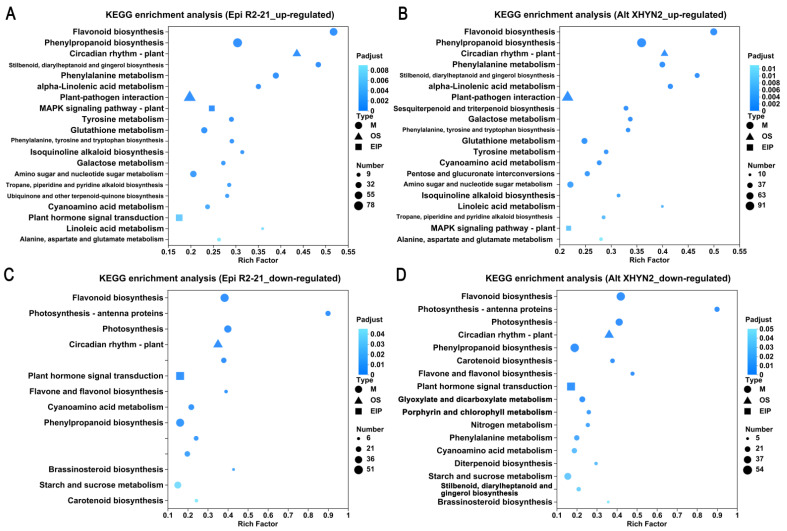
Scatter plot of KEGG pathways for the up– and downregulated DEGs in tissue–cultured seedlings after inoculation with Epi R2-21and Alt XHYN2. (**A**) Upregulated DEGs inoculated with Epi R2-21; (**B**) upregulated DEGs inoculated with Alt XHYN2; (**C**) downregulated DEGs inoculated with Epi R2-21. (**D**) Downregulated DEGs inoculated with Alt XHYN2. M: metabolism; OS: organismal systems; EIP: environmental information processing; HD: human diseases.

The functions of the up– and downregulated DEGs were further analysed to assess their metabolic pathways through KEGG enrichment analysis (Figure 5). The upregulated DEGs in the Epi R2-21 or Alt XHYN2 treatments, both enriched in the KEGG pathway, were found to be mainly involved in secondary metabolism (flavonoid, phenylpropanoid, phenylalanine, stilbenoid, diarylheptanoid and gingerol, and isoquinoline alkaloid, ubiquinone biosynthesis) and primary metabolism (α–linolenic acid metabolism, tyrosine, glutathione, galactose, and cyanoamino acid metabolism), suggesting that inoculation with Epi R2-21 and Alt XHYN2 reprograms the primary and secondary metabolism, putatively related to defence activation. The activation of stress responses involved in defence processes such as plant–pathogen interaction, plant MAPK signalling pathway, and circadian rhythm–plant was also observed (Figure 5A,B). Furthermore, the upregulated DEGs in Epi R2-21 treatments were involved in ubiquinone and other terpenoid–quinone biosynthesis and plant hormone signalling (Figure 5A), as well as sesquiterpenoid and triterpenoid biosynthesis, pentose. and glucuronate interconversions in Alt XHYN2 treatments (Figure 5B).

The downregulated DEGs in the Epi R2-21 and Alt XHYN2 treatments were significantly activated in some secondary metabolism (flavonoid, phenylpropanoid, flavone and flavonol biosynthesis, stilbenoid, diarylheptanoid. and gingerolbiosynthesis) and primary metabolism (porphyrin and chlorophyll metabolism, cyanoamino acid metabolism, carotenoid biosynthesis, starch and sucrose metabolism, glyoxylate and dicarboxylate metabolism, brassinosteroid biosynthesis) (Figure 5C,D). In addition, stress responses involved in defence processes were also observed, such as photosynthesis–antenna proteins, photosynthesis, circadian rhythm–plant, and plant hormone signalling (Figure 5C,D). Additionally, the downregulated DEGs in Alt XHYN2 treatments were involved in nitrogen metabolism, phenylalanine metabolism, and diterpenoid biosynthesis (Figure 5D).

### 3.4. DEGs Involved in Defence Responses Induced by the Endophytes Epi R2-21 and Alt XHYN2

**Figure 6:**
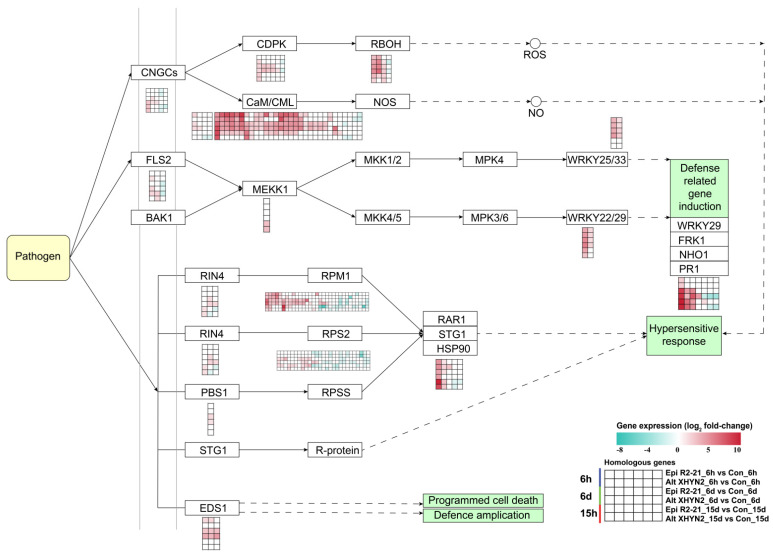
DEGs related to the plant−pathogen interaction in tissue−cultured seedlings of ‘Rose Honey’ after 6 h, 6 d, and 15 d inoculation with Epi R2-21 and Alt XHYN2 (Table S3). CNGCs, cyclic nucleotide gated channel; CDPK, calcium−dependent protein kinase; RBOH, respiratory burst oxidase; CaM, calmodulin; CML, calcium−binding protein CML; NOS, nitric oxide synthase; FLS2, serine/threonine−protein kinase FLS2; BAK1, BRI1−associated kinase 1; MEKK1, mitogen−activated extracellular signal−regulated kinase 1; MKK/MPK, mitogen−activated protein kinase kinase; WRKY33, WRKY transcription factor 33; WRKY22, WRKY transcription factor 22; PR1, pathogenesis−related protein 1; RIN4, RPM1−interacting protein 4; RPM1, disease resistance protein RPM1; RPS2, disease resistance protein RPS2; STG1, stay green 1; HSP90, heat shock protein 90; EDS1, enhanced disease susceptibility 1 protein.

**Figure 7:**
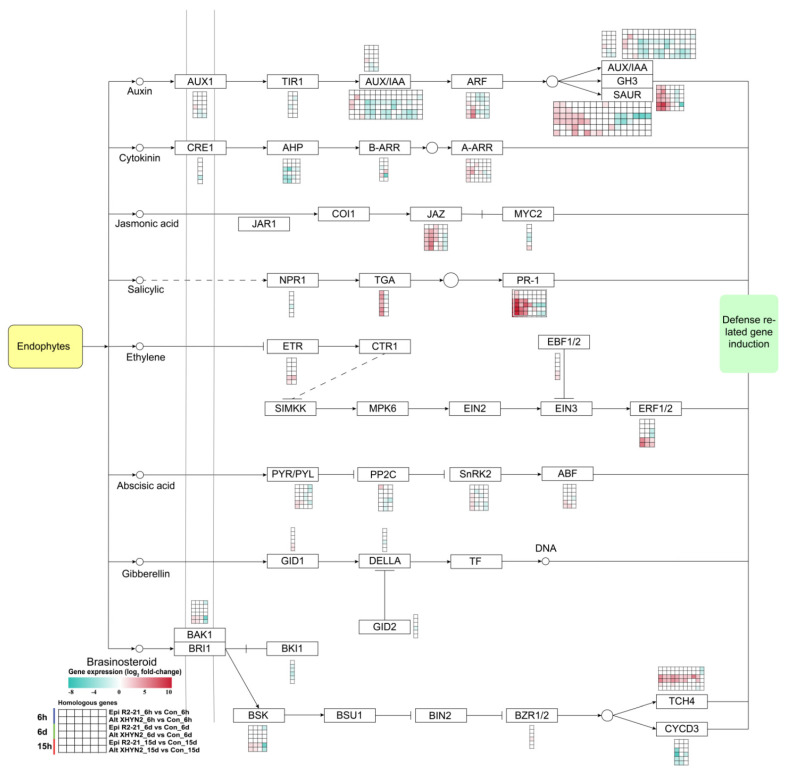
DEGs related to the plant hormone signal transduction in tissue−cultured seedlings of ‘Rose Honey’ after 6 h, 6 d, and 15 d inoculation with Epi R2-21 and Alt XHYN2 (Table S4). AUX1, auxin influx carrier; TIR1, transport inhibitor response 1; IAA, indole acetic acid; ARF, auxin response factor; SAUR, small auxin−up RNA, SAUR family protein; GH3, Gretchen Hagen 3; CRE1, cytokinin receptor; AHP, Arabidopsis histidine phosphotransfer protein; ARR−B, response regulator−B; ARR−A, response regulator−A; JAR1, jasmonic acid related Arabidopsis genes; JAZ, jasmonate ZIM−domain; MYC2, myelocytomatosis protein 2; NPR1, regulatory protein NPR1; TGA, transcription factor TGA; PR1, pathogenesis−related protein 1; ETR, ethylene receptor; CTR1, constitutive tripe response 1; SIMKK, a mitogen−activated protein kinase kinase; MPK6, mitogen−activated protein kinase kinase 6; EIN2/3, ethylene insensitive 2/3; EBF1/2, EIN3−binding F−box protein; ERF1/2, ethylene−responsive transcription factor 1/2; PYR/PYL, abscisic acid receptor; PP2C, protein phosphatase 2C; SnRK2, serine/threonine−protein kinase; ABF, ABA responsive element binding factor; GID1, gibberellin receptor GID1; GID2, F−box protein; DELLA, negative regulation factor of gibberellin; TF, transcription factor; BSK, BR−signalling kinase; BAK1, BRI1−associated kinase 1; BKI1, BRI1 kinase inhibitor 1; BSU1, bri1 suppressor 1; BIN2, brassinosteroid−insensitive 2; BZR1/2, brassinosteroid resistant 1/2; TCH4, touch gene 4; CYCD3, cyclin D3.

After inoculation with Epi R2-21 and Alt XHYN2 at 6 h, 6 d, and 15 d, a considerable number of DEGs related to defence response pathways, such as the plant–pathogen interaction, plant hormone signal transduction, plant MAPK signalling pathway, brassinoid steroid biosynthesis, glutathione metabolism pathway, were induced in tissue–cultured seedlings. In particular, most DEGs were induced in the plant–pathogen interaction and plant hormone signalling pathways (Figure 6 and Figure 7).

After 6 h, 6 d, and 15 d inoculation, a total of 140 DEGs (88 upregulated and 52 downregulated) related to the plant–pathogen interaction pathway were identified in tissue-cultured seedlings, but 117 and 123 DEGs were identified in Epi R2-21 and Alt XHYN2 treatments, respectively (Table S3). In the plant hormone signalling pathway, a total of 122 DEGs (59 upregulated and 63 downregulated) were induced, but 96 and 102 DEGs were identified in Epi R2-21 and Alt XHYN2 treatments, respectively (Table S4). Among the above DEGs, 10 upregulated genes (1.36–8.77–fold) were commonly expressed across the time points involved in the plant–pathogen interaction and the plant hormone signalling pathways (Figure S3). In the plant–pathogen interaction pathway, one gene encoding calmodulin–like protein (CML), one gene encoding pathogenesis–related protein PR1, two genes encoding disease resistance protein RPM1, one gene encoding respiratory burst oxidase (RBOH), one gene encoding transcription factor WRKY22, and one gene encoding heat shock protein HSP90 were upregulated. In the plant hormone signalling pathway, two genes encoding the auxin–responsive GH3 gene family GH3 and one gene encoding the transcription factor TGA were upregulated.

### 3.5. DEGs Involved in Secondary Metabolism Induced by the Endophytes Epi R2-21 and Alt XHYN2

**Figure 8:**
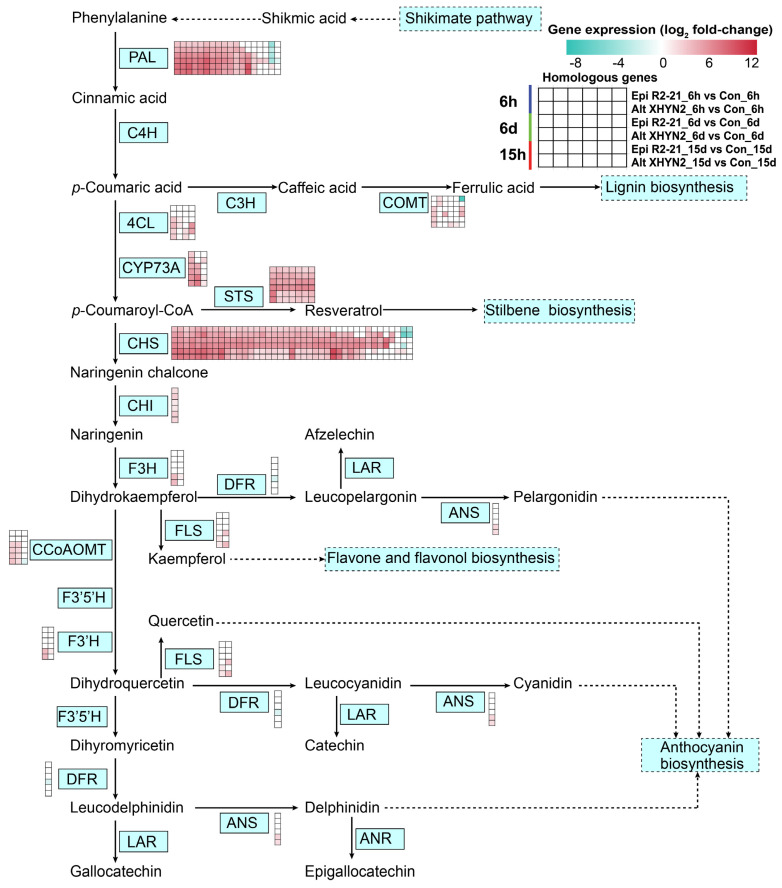
DEGs related to secondary metabolism in tissue−cultured seedlings of ‘Rose Honey’ after 6 h, 6 d, and 15 d inoculation with Epi R2−21 and Alt XHYN2. Omitted steps were represented by dashed lines. Heatmap colours represent the gene expression for each comparison (Tables S5–S8). PAL, phenylalanine ammonia−lyase; C4H, cinnamate 4−hydroxylase; C3H, cinnamate 3−hydroxylase; 4CL, 4−coumaroyl−CoA ligase; COMT, caffeic acid 3−*O*−methyltransferase; CYP73A, cytochrome P450; STS, stilbene synthase; CHS, chalcone synthase; CHI, chalcone isomerase; F3H, flavonone 3−hydroxylase; DFR, dihydroflavanol 4−reductase; LAR, leucoanthocyanidin reductase; CCoAOMT, caffeoyl−CoA *O*−methyltransferase; FLS, flavonol synthase; F3′5′H, flavonoid−3′,5′−hydroxylase; F3′H, flavonoid 3′−monooxygenase; ANS, anthocyanidin synthase.

In particular, secondary metabolism, such as phenylpropanoid, flavonoid, and stilbenes biosynthesis, was affected by Epi R2-21 and Alt XHYN2 after inoculation in tissue–cultured seedlings for 6 h, 6 d, and 15 d (Figure 8). According to the KEGG pathway analysis, 39 and 44 DEGs involved in the phenylalanine metabolism pathway were identified in tissue–cultured seedlings inoculated with Epi R2-21 and Alt XHYN2, respectively (Table S5). In the phenylpropanoid biosynthesis pathway (Table S6), 98 and 120 DEGs were identified after inoculation with Epi R2-21 and Alt XHYN2, respectively. Among them, 12 upregulated DEGs were commonly differentially expressed in the above two pathways at all time points. In the flavonoid biosynthesis pathway (Table S7), 69 and 67 DEGs were identified after inoculation with Epi R2-21 and Alt XHYN2, respectively. Among them, 29 upregulated DEGs were commonly expressed at all time points. In the stilbenoid, diarylheptanoid, and gingerol biosynthesis pathway (Table S8), 32 and 30 DEGs were identified after inoculation with the endophytes Epi R2-21 and Alt XHYN2, respectively, of which 10 upregulated DEGs were commonly differentially expressed across the time points. Among the above DEGs, 43 upregulated genes (1.24–8.55–fold) were commonly differentially expressed across the time points (Figure S4). In addition, the gene *VIT_11s0065g00350* encoding transcinnamate 4–monooxygenase (CYP73A) was involved in phenylalanine metabolism, phenylpropanoid biosynthesis, the flavonoid biosynthesis, stilbenoid, diarylheptanoid, and gingerol biosynthesis pathways; 11 genes (*VIT_16s0039g01240*, *VIT_00s2849g00010*, *VIT_00s2508g00010*, *VIT_16s0039g01110*, *VIT_16s0039g01300*, *VIT_16s0039g01360*, *VIT_16s0039g01130*, *VIT_16s0039g01280*, *VIT_16s0039g01120*, *VIT_16s0039g01170*, *VIT_08s0040g01710*) encoding phenylalanine ammonia–lyase (PAL) were involved in the phenylalanine metabolism and phenylpropanoid biosynthesis pathways; 6 genes (*VIT_16s0100g00810*, *VIT_16s0100g00960*, *VIT_16s0100g00950*, *VIT_16s0100g00860*, *VIT_16s0100g01130*, *VIT_10s0042g00870*) commonly encoding chalcone synthase (CHS) and stilbene synthase (STS) were involved in the flavonoid biosynthesis, stilbenoid, diarylheptanoid, and gingerol biosynthesis pathways.

### 3.6. PPI Analysis of Key Genes Related to Secondary Metabolism against Endophyte Defence

**Figure 9:**
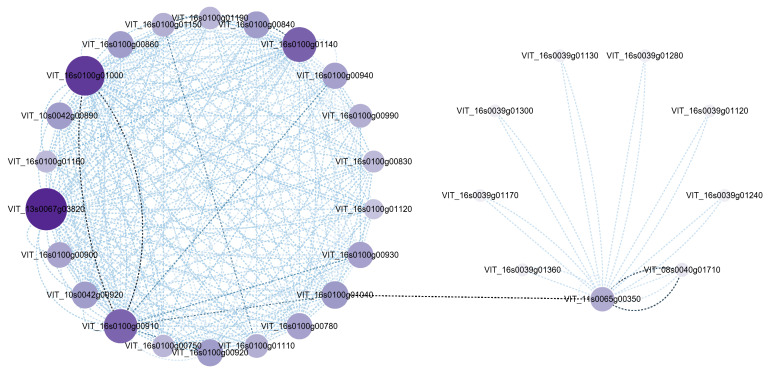
Network analysis of common DEGs related to secondary metabolism in tissue–cultured seedlings of ‘Rose Honey’ after inoculation with Epi R2-21 and Alt XHYN2. The size of each node corresponds to the degree (number of connections), and the node colour from dark to light represents the highest to lowest betweenness centrality.

At the same time, an interaction network, based on the common upregulated DEGs related to secondary metabolism in tissue–cultured seedlings of ‘Rose Honey’ after inoculation with Epi R2-21 and Alt XHYN, was constructed to select key genes involved in the production of stress–related secondary metabolites (Figure 9). The network consisted of two clusters with 37 nodes and 300 edges. In cluster I, the gene *VIT_13s0067g03820* and the genes *VIT_16s0100g01000*, *VIT_16s0100g00910*, and *VIT_16s0100g01140*, encoding CHI and CHS, respectively, involved in the flavonoid biosynthesis, had the highest betweenness centrality scores. In addition, the gene *VIT_11s0065g00350* in cluster II was connected to cluster I by the gene *VIT_16s0100g01040*. The gene *VIT_11s0065g00350*, encoding CYP73A, was commonly involved in the phenylalanine metabolism, phenylpropanoid biosynthesis, flavonoid biosynthesis, stilbenoid, diarylheptanoid, and gingerol biosynthesis pathways. It is predicted that the above genes play a positive regulatory role in endophyte defence and metabolite production.

## 4. Discussion

As a crucial cooperative partner of the grapevine, endophytes strongly influence the growth of the host plant, even the physiological metabolism [3]. During the long–term interaction, endophytes and their host grapevine establish a mutualistic symbiotic relationship. The genetics and metabolism of the endophytes would modify the metabolic pathway of their host plants and vice versa [25,26]. Most of the studies have tried to explore the reciprocal mechanism of the complex interaction between grapevine and endophytes, but there is still a great gap in knowledge about the exact mechanisms of the interaction. Today, multi–omics technology can provide insight into the interactions between host plants and endophytes. In our study, the different responses of tissue–cultured seedlings of the cultivar ‘Rose Honey’ inoculated with Epi R2-21 and Alt XHYN2 at three different time points were analysed at the transcriptomic level, compared to our previous study carried out on the cultivar ‘Cabernet Sauvignon’. This work has attempted to illustrate the differential response between grapevine cultivars and the interactions between endophytes and their host grapevine, as well as give insights for grape quality management in viticulture through the use of fungal endophytes.

### 4.1. Endophytic–Induced Resistance in Grapevines, Similar to Pathogen Invasion

Endophytic fungi undergo a series of complex processes as mycorrhizal and pathogenic fungi, including spore and affinity host recognition, activation, adhesion to surface substrates, germination, and invasion into the interior of the tissue, as well as penetration of the host plant’s mechanical barrier and overcoming its defence response [27]. However, similar to mycorrhizal fungi, symbiotic fungi have been reported to induce a weaker plant defence response than pathogenic fungi, presumably due to endophytic fungi with lower virulence factors, the mycelial secretion of certain glycoproteins, or a change in cell wall composition that weakens the host’s ability to strongly recognise them [7,28].

It has been reported that fungi infect the host grapevine as follows: the fungus/spore adheres to the surface of the tissue, then produces the infection structures for attaching stably to the host, invades the host tissue, and successfully colonizes and spreads within the host [29,30]. During infection, fungi release various metabolites that are detrimental to the plant host, i.e., known as virulence factors, including related enzymes and toxins. In turn, the grapevine responds to the fungi by activating defence responses and downstream resistance signalling against invasion and colonization, such as the stimulation of defence–related enzymes, phytohormones, and pathogen-related proteins [31]. 

The pathogen–associated molecular patterns (PAMPs)–triggered immunity (PTI) and effector (virulence protein)–triggered immunity (ETI) were the primary defence responses [32]. Pattern recognition receptors (PRRs) recognise PAMPs and induce mitogen–activated protein kinases (MAPKs) and calcium signalling; subsequently, MAPKs and calcium signalling activate defence responses to suppress pathogen colonization [33,34]. However, endophytes have evolved numerous virulence effectors to avoid PTI and achieve infection. In turn, the host plants have also evolved genes that can recognise specifical effectors and trigger a second host immune response, termed ETI, to restrict pathogen growth [35]. 

Cytoplasmic Ca^2+^ rapidly accumulates when plants perceive PAMPs, calcium–dependent protein kinases (CDPKs), RBOH, calmodulin (CaM), and CML produce reactive oxygen species (ROS) and nitric oxide (NO) separately to activate plant defence responses [36]. In our study, we identified several genes, including cyclic nucleotide–gated channels (CNGCs), CDPK, RBOH, CaM, and CML, which were upregulated to some extent after Epi R2-21 and Alt XHYN2 infection. Additionally, once plants recognise PAMPs, MAPK cascades are activated followed by WRKY transcription factors that induce the expression of defence–related genes [32,37]. Here, we observed that the MAPK signalling pathway was upregulated in grapevine seedlings after inoculation with Epi R2-21 and Alt XHYN2, and we identified flagellin–sensing 2 (FLS2), mitogen–activated protein kinase kinase 1 (MEKK1), WRKY transcription factor 22 (WRKY22/29), and WRKY transcription factor 33 (WRKY 25/33). While in the ETI pathway, PRM1–interacting protein 4 (RIN4), *R* genes (RPM1 and RPS2), disease resistance protein (PBS1), enhanced disease susceptibility protein (EDS1), and heat shock protein 90 (HSP90) had variable expression levels, most of which were upregulated or downregulated over time, suggesting that genes would be reprogrammed when Epi R2-21 and Alt XHYN2 invade host grapevine. 

### 4.2. Fungal Endophytes Inoculation Induced the Expression of Genes Involved in Phytohormones and Secondary Metabolites in Tissue-Cultured Grapevine Seedlings 

The major phytohormones, such as salicylic acid (SA), jasmonic acid (JA), indole–3–acetic acid (IAA), abscisic acid (ABA), and ethylene (ET), play an important role in plant growth, plant immune response via transduction of cellular signals, and the induction of defence–related gene expression [30]. The transcriptome combined with the metabolome and hormone metabolism revealed that grape berries (*Vitis vinifera* L. cv. Carignan) had an activated defence response involving salicylic acid and jasmonates, along with the accumulation of defence–related genes and metabolites, such as phenylpropanoids and fatty acids [38]. During infection with the pathogen *Lasiodiplodia theobromae*, the homologous DEGs in the auxin transduction pathway were all significantly upregulated, and DEGs in the cytokinin, JA, SA, and ET transduction pathways were also mainly upregulated [13]. After inoculation with the pathogen *Plasmopara viticola*, the defence responses in *V. vinifera* cv. Mgaloblishvili were mainly mediated by ET [39]. In our study, these phytohormones were fine–tuned due to Epi R2-21 and Alt XHYN2 infection, especially auxin, JA, and SA (Figure 6; Table S3). Auxin signalling is critical for plant resistance to the necrotrophic fungi *Plectosphaerella cucumerina* and *Botrytis cinerea* [40]. And 59 upregulated and 63 downregulated DEGs were induced in the plant hormone signalling pathway in our study. The homologous genes *Aux/IAA* and *TIR1* were mostly downregulated in response to Epi R2-21 and Alt XHYN2, while the homologous genes *SAUR* and *GH3* were mostly upregulated. In addition, the JA– and SA–mediated defence response is critical in the fight against pathogens. The genes *PR-1*, *JAZ*, and *TGA*, which are involved in JA or SA biosynthesis and signalling, were significantly upregulated in grapevine seedlings, in agreement with previous studies carried out with the fungal pathogens *Lasiodiplodia theobromae* on grapevine and *Schizaphis graminum* on wheat [41,42]. However, in our study, some genes also involved in cytokinin, IAA, ABA, gibberellin, and brasinosteoid signalling, such as *CRE1*, *AHP*, *PP2C*, *SnRK2*, *DELLA*, *GID2*, *BKI1*, and *CYCD3*, were transcriptionally affected by Epi R2-21 and Alt XHYN2, being mostly upregulated in the early phase of the infection and downregulated at later stages.

Secondary metabolites can act as physical and chemical barriers to pathogen infection and induce defence–related gene expression in plants to inhibit fungal growth and rot development [43,44]. Similarly, pathogenic fungal invasion leads to the upregulation of genes involved in primary and secondary metabolism related to the defence response in grapes. The susceptible *Vitis vinifera* subsp. *vinifera* and the tolerant *Vitis vinifera* subsp. *sylvestris* inoculated with *Neofusicoccum parvum* displayed great changes in both primary and specialized metabolites, especially stilbene and flavonoid compounds [45]. In our study, some of the secondary metabolic pathways were upregulated in infected grapevines seedlings, such as flavonoid biosynthesis, phenylpropanoid biosynthesis, phenylalanine metabolism, stilbenoid, diarylheptanoid, and gingerol biosynthesis, etc. These pathways may be involved in the fungal resistance mechanism of grapevine (Figure 7). Similarly, phenylalanine, phenylpropanoid, and stilbenoid biosynthesis were enriched with several upregulated genes in grape berries from *Vitis vinifera* cv. Trincadeira with *Botrytis cinerea* infection [46]. Furthermore, the *STS* and *ROMT* genes were also expressed at higher levels in grapes infected with *Aspergillus niger* [47].

In addition, transcriptomic analysis revealed that several pathways related to plant growth and development were depressed in grapevine seedlings challenged with Epi R2-21 and Alt XHYN2, such as photosynthesis, photosynthesis–antenna proteins, carbon fixation in photosynthetic organisms, pentose phosphate pathway, porphyrin and chlorophyll metabolism, N–glycan biosynthesis, ribosome, spliceosome, etc. (Table 1). The results showed that inoculation with Epi R2-21 and Alt XHYN2 led to a reduction in photosynthetic activity and subsequently inhibited the growth of grapevine seedlings. As proposed by the optimal defence theory, secondary metabolites are produced when plants experience environmental stress, which act as a defence but reduce plant growth until the plant and its symbiotic fungi reach an equilibrium [48]. 

### 4.3. Differences in Responses between Endophyte Strains and Grapevine Cultivars

Together with these transcriptome data and our previous study [17], it was shown that tissue–cultured seedlings rapidly initiated a large number of DEGs due to infection with Epi R2-21 and Alt XHYN2 in either the ‘Cabernet Sauvignon’ or ‘Rose Honey’ cultivars. 

Regarding the grape cultivars, the response trends were similar. For both the grape cultivars ‘Cabernet Sauvignon’ and ‘Rose Honey’, the endophyte Alt XHYN2 inoculation triggered a stronger response than the endophyte Epi R2-21, most likely due to the fact that the endophyte Alt XHYN2 belongs to the fungal pathogen *Alternaria* and lives in a mutualistic relationship or as a pathogen causing disease in the host plant [49]. With the exception of the samples of endophyte Alt XHYN2 after 15 d of inoculation on the cultivar ‘Rose Honey’, DEGs were more abundant on the cultivar ‘Rose Honey’ than on the cultivar ‘Cabernet Sauvignon’, especially in terms of the downregulated DEGs, suggesting that tissue–cultured seedlings of the cultivar ‘Rose Honey’ induced stronger defence responses to endophyte infection, which is most likely due to the fact that the cultivar ‘Cabernet Sauvignon’ is a worldwide planted wine grape variety with strong vitality and good adaptability.

Although the transcriptional responses of the two grape cultivar (‘Cabernet Sauvignon’ and ‘Rose Honey’) were very similar, some differences could be observed. In ‘Rose Honey’, all DEGs (up– and downregulated) increased over time during Epi R2-21 infection, whereas in ‘Cabernet Sauvignon’, only the upregulated DEGs followed this trend. An opposite situation occurred with Alt XHYN2, as all DEGs (up– and downregulated) increased over time in ‘Cabernet Sauvignon’, but not in ‘Rose Honey’. Here, the downregulated DEGs increased over time, while the total DEGs and the upregulated DEGs increased from 6 h to 6 d, but then tended to decrease from 6 d to 15 d.

In addition, according to the PPI network, the genes *VIT_16s0100g00910* encoding CHS involved in the flavonoid biosynthesis, and *VIT_11s0065g00350* encoding CYP73A commonly involved in phenylalanine metabolism, phenylpropanoid biosynthesis, flavonoid biosynthesis, stilbenoid, diarylheptanoid and gingerol biosynthesis pathways, were suggested to play a crucial role in endophyte defense and metabolite production in both the ‘Cabernet Sauvignon’ and ‘Rose Honey’ cultivars.

## 5. Conclusions

Based on transcriptome analyses performed on the the cultivar ‘Rose Honey’ in this study and the cultivar ‘Cabernet Sauvignon’ in our previous study, it was shown that grapevine transcriptomes were remarkably regulated in response to inoculation with endophytes Epi R2-21 and Alt XHYN2 in tissue-cultured seedlings of different grapevine cultivars. This give us an indication that we can prepare the suspensions of fungal endophytes to be sprayed on the berries at the stage of the berry ripening to improve the quality of the grapes and the resulting wines.

In addition, the genes *VIT_16s0100g00910,* encoding CHS, and *VIT_11s0065g00350*, encoding CYP73A, were involved in secondary metabolism in both ‘Rose Honey’ and ‘Cabernet Sauvignon’ cultivars, suggesting the use of the two candidate genes for further study of the mechanism on the effects and mechanisms of endophytes involved in secondary metabolism modulations in grapevine, and thus improving grape quality and resultant products, such as wine, in practice. Together with the results on ‘Cabernet Sauvignon’ and ‘Rose Honey’ after inoculation with the endophytes Epi R2-21 and Alt XHYN2, it was found that tissue–cultured seedlings of the cultivar ‘Rose Honey’ induced stronger defence responses to fungal endophyte infection than those of ‘Cabernet Sauvignon’, and inoculation with the endophyte Alt XHYN2 triggered a stronger response than inoculation with the endophyte Epi R2-21. This study would suggest the use of candidate fungal endophytes as a strategy for shaping the metabolic profiles of grapes on different grape cultivars in viticulture. Overall, this study fills in the gaps in our knowledge of grapevine resistance to fungal endophytes, deepens our understanding of the host plant response to fungal endophytes, and will help us to better understand the molecular interactions between grapevine and fungal endophytes.

## Figures and Tables

**Table 1:** KEGG pathway analysis of the significantly time–course DEGs of the tissue–cultured seedlings with Epi R2-21 and Alt XHYN2 at 6 h, 6 d, and 15 d after inoculation, respectively.

Cluster name	Number of DEGs	KEGG pathway	Cluster name	Number of DEGs	KEGG pathway
R2-21			XHYN2		
DEGs were consistently upregulated as compared to control			DEGs were consistently upregulated as compared to control		
Cluster 1	20	Plant–pathogen interaction	Cluster 7	21	Citrate cycle (TCA cycle)
Cluster 7	13	Amino sugar and nucleotide sugar metabolism		45	Protein processing in endoplasmic reticulum
	7	Phenylalanine metabolism		26	Glycolysis/Gluconeogenesis
	5	Stilbenoid, diarylheptanoid, and gingerol biosynthesis		12	Protein export
	6	Flavonoid biosynthesis		14	Proteasome
	6	Circadian rhythm–plant		13	Phenylalanine, tyrosine, and tryptophan biosynthesis
	5	Arginine and proline metabolism		19	Pyruvate metabolism
	3	Ether lipid metabolism		9	Pantothenate and CoA biosynthesis
	4	Protein export		18	Phagosome
	4	Fatty acid degradation		23	Endocytosis
	4	beta-Alanine metabolism		10	Fatty acid degradation
	4	alpha-Linolenic acid metabolism		7	Valine, leucine and isoleucine biosynthesis
Cluster 8	17	Citrate cycle (TCA cycle)		8	N-Glycan biosynthesis
	34	Protein processing in endoplasmic reticulum		13	Peroxisome
	19	Glycolysis/Gluconeogenesis		20	Amino sugar and nucleotide sugar metabolism
	22	Endocytosis		5	Histidine metabolism
	10	Fatty acid degradation		15	Cysteine and methionine metabolism
	9	Protein export	Cluster 8	16	Flavonoid biosynthesis
	13	Peroxisome		15	Circadian rhythm–plant
	10	Phenylalanine, tyrosine, and tryptophan biosynthesis		12	Phenylalanine metabolism
	14	Pyruvate metabolism		7	Stilbenoid, diarylheptanoid, and gingerol biosynthesis
	6	Valine, leucine, and isoleucine biosynthesis		12	Amino sugar and nucleotide sugar metabolism
	14	Phagosome		7	alpha-Linolenic acid metabolism
	9	Proteasome		7	Valine, leucine, and isoleucine degradation
	8	Propanoate metabolism		6	Fatty acid degradation
	13	Cysteine and methionine metabolism		13	Phenylpropanoid biosynthesis
	9	alpha-Linolenic acid metabolism		4	Ether lipid metabolism
	9	Valine, leucine, and isoleucine degradation		3	Arachidonic acid metabolism
	6	Sulfur metabolism		5	Inositol phosphate metabolism
	8	Pentose phosphate pathway		6	Tyrosine metabolism
	10	Glyoxylate and dicarboxylate metabolism	DEGs were consistently downregulated as compared to control		
	6	Pantothenate and CoA biosynthesis	Cluster 1	12	Porphyrin and chlorophyll metabolism
	2	Caffeine metabolism		38	Ribosome
	9	Glycine, serine, and threonine metabolism		14	Aminoacyl-tRNA biosynthesis
DEGs were consistently down-egulated as compared to control				14	Glyoxylate and dicarboxylate metabolism
Cluster 2	12	Carbon fixation in photosynthetic organisms		9	Alanine, aspartate and glutamate metabolism
	5	Photosynthesis—antenna proteins		7	Carotenoid biosynthesis
	7	Glyoxylate and dicarboxylate metabolism		7	Butanoate metabolism
	6	Pentose phosphate pathway		6	One carbon pool by folate
	6	Circadian rhythm–plant	Cluster 2	9	Spliceosome
	8	mRNA surveillance pathway	Cluster 3	13	Photosynthesis—antenna proteins
	4	Porphyrin and chlorophyll metabolism		20	Photosynthesis
Cluster 3	22	Photosynthesis		12	Carbon fixation in photosynthetic organisms
	7	Photosynthesis—antenna proteins		7	Porphyrin and chlorophyll metabolism
Cluster 4	171	Ribosome	Cluster 5	104	Ribosome
	29	Spliceosome		20	Ribosome biogenesis in eukaryotes
	17	Ribosome biogenesis in eukaryotes		24	Spliceosome
	9	N-Glycan biosynthesis		12	Proteasome
	5	Lysine biosynthesis		15	Pyruvate metabolism
	9	Protein export		10	Pentose phosphate pathway
Cluster 5	13	Spliceosome		8	Protein export
Cluster 6	17	Spliceosome	DEGs were first upregulated but then downregulated as compared to control		
DEGs were first downregulated but then upregulated as compared to control			Cluster 4	13	Plant–pathogen interaction
Cluster 9	None		DEGs were first downregulated but then upregulated as compared to control		
			Cluster 6	14	Protein processing in endoplasmic reticulum
			Cluster 9	15	Spliceosome
				9	Ubiquitin-mediated proteolysis

## Data Availability

The datasets for this study can be found in the Sequence Read Archive (SRA) under the study accession number PRJNA667208.

## Supplementary Materials

The following supporting information can be downloaded at: https://www.mdpi.com/article/10.3390/metabo14080402/s1, Figure S1: Fungal endophytes Epi R2-21 (A, C) and Alt XHYN2 (B, D) used in this study. Tissue–cultured grapevine seedlings of ‘Rose Honey’ after 15 d from the inoculation with Epi R2-21 (E), Alt XHYN2 (F) or not inoculation (G). Figure S2: PCA (A), DEGs analysis (B), Venn diagram of the common and unique upregulated (C) or downregulated DEGs analysis (D) of tissue–cultured grapevine seedlings of ‘Rose Honey’ after 6 h, 6 d and 15 d from the inoculation with Epi R2-21 and Alt XHYN2. Figure S3: Common DEGs related to secondary metabolism in tissue–cultured grapevine seedlings of ‘Rose Honey’ after co–cultured with Epi R2-21 and Alt XHYN2. Figure S4: Common DEGs related to disease resistance in tissue–cultured grapevine seedlings of ‘Rose Honey’ after co–cultured with Epi R2-21 and Alt XHYN2. Table S1: Primer sequences used in the qRT–PCR. Table S2: Quality assessment of RNA–seq data from the tissue–cultured seedlings of ‘Rose Honey’ with Epi R2-21 and Alt XHYN2 after 6 h, 6 d, and 15 d, inoculation, respectively. Table S3: DEGs related to the plant–pathogen interaction pathway of the tissue–cultured seedlings of ‘Rose Honey’ in response to endophytes fungi Epi R2-21 and Alt XHYN2 after 6 h, 6 d, and 15 d inoculation, respectively (|Log_2_FC| ≥ 1; *p*–adjust < 0.05). Table S4: DEGs related to the plant hormone signal transduction pathway of the tissue–cultured seedlings of ‘Rose Honey’ in response to endophytes fungi Epi R2-21 and Alt XHYN2 after 6 h, 6 d, and 15 d inoculation, respectively (|Log_2_FC| ≥ 1; *p*–adjust < 0.05). Table S5: DEGs related to the phenylalanine metabolism pathway of the tissue–cultured seedlings of ‘Rose Honey’ in response to endophytes fungi Epi R2-21 and Alt XHYN2 after 6 h, 6 d, and 15 d inoculation, respectively (|Log_2_FC| ≥ 1; *p*–adjust < 0.05). Table S6: DEGs related to the phenylpropanoid biosynthesis pathway of the tissue–cultured seedlings of ‘Rose Honey’ in response to endophytes fungi Epi R2-21 and Alt XHYN2 after 6 h, 6 d, and 15 d inoculation, respectively (|Log_2_FC| ≥ 1; *p*–adjust < 0.05). Table S7: DEGs related to the flavonoid biosynthesis pathway of the tissue–cultured seedlings of ‘Rose Honey’ in response to endophytes fungi Epi R2-21 and Alt XHYN2 after 6 h, 6 d, and 15 d inoculation, respectively (|Log_2_FC| ≥ 1; *p*–adjust < 0.05). Table S8: DEGs related to the stilbenoid, diarylheptanoid and gingerol biosynthesis pathway of the tissue–cultured seedlings of ‘Rose Honey’ in response to endophytes fungi Epi R2-21 and Alt XHYN2 after 6 h, 6 d, and 15 d inoculation, respectively (|Log_2_FC| ≥ 1; *p*–adjust < 0.05).
