# Supplementary material for: Cultivation of Fungal Endophytes with Tissue Culture Grapevine Seedlings Reprograms Metabolism by Triggering Defence Responses
Source: Metabolites. 2024 Jul 24;14(8):402. doi: 10.3390/metabo14080402 (PMC11356313; doi:10.3390/metabo14080402)
Supplement: Supplementary file 1 [file metabolites-14-00402-s001.zip › Supplementary tables.pdf]

**Table S1. Primer sequences used in the qRT-PCR.**

| **Gene ID** | | **Sequences (5'-3')** |
|---|---|---|
| *VIT_16s0039g01300* | F | GAGCAAAGGCACCGACAG |
| | R | TCCGTTCCCAAATATCCC |
| *VIT_14s0060g02320* | F | GCCACAAGACGAGCCTAC |
| | R | CCCGAGTCCCTGAAACTA |
| *VIT_16s0100g00840* | F | ATGGACGAGATGAGAAAG |
| | R | CTAGGAATACTGTGGAGGA |
| *VIT_11s0065g00350* | F | CTGTAGCAGGTGGGAAGG |
| | R | TATGCGGTGATTGGAGTG |
| *VIT_10s0042g00870* | F | GCAGCCTAAGTCCAAGAT |
| | R | GTTTCAGAAGGTCCACGA |
| *VIT_05s0094g00200* | F | TCGTATCCTCGGTTTGGC |
| | R | GCATCGGTTGCTACTATTTC |
| *EF1* | F | AGACCACCAAATACTACTGC |
| | R | GATCATCTGCCTGACACC |

Note: F and R represent the upstream and downstream primer, respectively.

**Table S2.** Quality assessment of RNA-seq data from the tissue cultured seedlings of 'Rose Honey' with Epi R2-21 and Alt XHYN2 after 6 h, 6 d, and 15 d inoculation, respectively.

| Samples | Raw reads | Clean reads | Clean Bases | Error% | GC% | Q20% | Q30% | Mapped Reads | Multiple Mapped | Uniquely Mapped | Exons | Introns | Intergenic |
|---|---|---|---|---|---|---|---|---|---|---|---|---|---|
| Con_6h_1 | 54,184,136 | 53,773,580 | 8,016,046,049 | 0.02 | 46.31 | 98.81 | 96.09 | 49,890,636(92.78%) | 1437033(2.67%) | 48453603(90.11%) | 94.72% | 3.60% | 1.69% |
| Con_6h_2 | 55,242,922 | 54,704,358 | 8,145,765,795 | 0.02 | 46.28 | 98.71 | 95.88 | 51,012,338(93.25%) | 1437903(2.63%) | 49574435(90.62%) | 94.91% | 3.49% | 1.61% |
| Con_6h_3 | 60,270,018 | 59,740,710 | 8,914,374,014 | 0.02 | 46.18 | 98.69 | 95.75 | 55,479,546(92.87%) | 1698555(2.84%) | 53780991(90.02%) | 94.64% | 3.73% | 1.64% |
| Con_6d_1 | 57,683,732 | 57,235,856 | 8,554,525,029 | 0.02 | 46.20 | 98.76 | 95.94 | 52,911,684(92.44%) | 1364777(2.38%) | 51546907(90.06%) | 93.25% | 4.88% | 1.87% |
| Con_6d_2 | 51,509,520 | 51,018,298 | 7,631,403,560 | 0.02 | 46.18 | 98.46 | 95.08 | 46,928,279(91.98%) | 1249455(2.45%) | 45678824(89.53%) | 93.25% | 4.91% | 1.83% |
| Con_6d_3 | 52,976,228 | 52,463,688 | 7,801,067,565 | 0.02 | 46.18 | 98.73 | 95.92 | 48,375,547(92.21%) | 1303921(2.49%) | 47071626(89.72%) | 93.26% | 4.80% | 1.94% |
| Con_15d_1 | 54,682,760 | 54,045,972 | 8,055,109,650 | 0.02 | 45.87 | 98.75 | 95.99 | 50,219,628(92.92%) | 1280655(2.37%) | 48938973(90.55%) | 93.82% | 4.59% | 1.60% |
| Con_15d_2 | 52,864,836 | 52,445,768 | 7,824,746,388 | 0.02 | 46.31 | 98.71 | 95.81 | 48,438,377(92.36%) | 1156915(2.21%) | 47281462(90.15%) | 93.79% | 4.50% | 1.71% |
| Con_15d_3 | 49,281,910 | 48,750,010 | 7,287,229,445 | 0.02 | 46.30 | 98.68 | 95.74 | 45,016,992(92.34%) | 1066974(2.19%) | 43950018(90.15%) | 93.39% | 4.78% | 1.82% |
| R2-21_6h _1 | 57,101,718 | 56,590,196 | 8,450,816,708 | 0.02 | 46.35 | 98.78 | 95.99 | 52,660,487(93.06%) | 1672180(2.95%) | 50988307(90.1%) | 94.70% | 3.53% | 1.77% |
| R2-21_6h _2 | 52,700,918 | 52,278,792 | 7,800,010,587 | 0.02 | 46.31 | 98.74 | 95.88 | 48,627,810(93.02%) | 1555492(2.98%) | 47072318(90.04%) | 94.71% | 3.55% | 1.74% |
| R2-21_6h _3 | 56,227,034 | 55,719,602 | 8,320,442,695 | 0.02 | 46.26 | 98.73 | 95.87 | 51,875,057(93.10%) | 1633869(2.93%) | 50241188(90.17%) | 94.67% | 3.60% | 1.73% |
| R2-21_6d _1 | 58,400,072 | 57,981,860 | 8,632,502,925 | 0.02 | 46.38 | 98.78 | 96.00 | 53,547,967(92.35%) | 1679195(2.9%) | 51868772(89.46%) | 94.56% | 3.76% | 1.69% |
| R2-21_6d _2 | 53,901,072 | 53,552,218 | 7,993,111,290 | 0.02 | 46.65 | 98.76 | 95.93 | 48,438,753(90.45%) | 1612578(3.01%) | 46826175(87.44%) | 94.17% | 3.91% | 1.93% |
| R2-21_6d _3 | 59,502,084 | 58,958,162 | 8,776,985,547 | 0.02 | 46.84 | 98.72 | 95.87 | 53,302,819(90.41%) | 1681938(2.85%) | 51620881(87.56%) | 94.60% | 3.62% | 1.78% |
| R2-21_15d _1 | 55,551,398 | 55,139,602 | 8,237,232,616 | 0.02 | 46.43 | 98.71 | 95.78 | 50,600,394(91.77%) | 1641359(2.98%) | 48959035(88.79%) | 94.35% | 3.93% | 1.71% |
| R2-21_15d _2 | 54,199,628 | 53,772,752 | 8,037,240,907 | 0.02 | 46.72 | 98.70 | 95.77 | 47,440,147(88.22%) | 1951694(3.63%) | 45488453(84.59%) | 93.73% | 4.47% | 1.80% |
| R2-21_15d _3 | 55,053,364 | 54,650,894 | 8,179,037,704 | 0.02 | 46.56 | 98.70 | 95.75 | 49,665,369(90.88%) | 2635114(4.82%) | 47030255(86.06%) | 94.93% | 3.39% | 1.67% |
| XHYN2_6h _1 | 49,137,744 | 48,756,496 | 7,329,671,392 | 0.02 | 45.76 | 98.72 | 95.82 | 45,419,963(93.16%) | 1756901(3.6%) | 43663062(89.55%) | 94.71% | 3.77% | 1.53% |
| XHYN2_6h _2 | 53,514,166 | 53,061,792 | 7,932,430,397 | 0.02 | 46.19 | 98.76 | 95.95 | 49,154,713(92.64%) | 1766733(3.33%) | 47387980(89.31%) | 94.60% | 3.66% | 1.74% |
| XHYN2_6h _3 | 52,294,012 | 51,952,030 | 7,769,568,289 | 0.02 | 46.19 | 98.74 | 95.86 | 48,319,463(93.01%) | 1878174(3.62%) | 46441289(89.39%) | 95.00% | 3.30% | 1.70% |
| XHYN2_6d _1 | 51,770,382 | 51,360,932 | 7,652,187,801 | 0.02 | 46.46 | 98.77 | 95.97 | 47,170,921(91.84%) | 1483465(2.89%) | 45687456(88.95%) | 93.97% | 4.11% | 1.92% |
| XHYN2_6d _2 | 57,695,812 | 57,242,472 | 8,544,401,330 | 0.02 | 46.35 | 98.75 | 95.93 | 52,487,597(91.69%) | 1828469(3.19%) | 50659128(88.5%) | 93.68% | 4.47% | 1.85% |
| XHYN2_6d _3 | 50,676,370 | 50,202,096 | 7,498,364,423 | 0.02 | 46.4 | 98.74 | 95.94 | 45,645,636(90.92%) | 1705478(3.4%) | 43940158(87.53%) | 93.62% | 4.43% | 1.95% |

| | | | | | | | | | | | | | |
|---|---|---|---|---|---|---|---|---|---|---|---|---|---|
| XHYN2_15d _1 | 53,005,684 | 52,685,876 | 7,870,226,413 | 0.02 | 46.15 | 98.77 | 95.95 | 48,728,933(92.49%) | 1549075(2.94%) | 47179858(89.55%) | 94.07% | 4.17% | 1.76% |
| XHYN2_15d _2 | 58,746,294 | 58,249,400 | 8,667,421,721 | 0.02 | 46.44 | 98.77 | 95.99 | 53,235,224(91.39%) | 1894021(3.25%) | 51341203(88.14%) | 94.58% | 3.79% | 1.63% |
| XHYN2_15d _3 | 57,458,414 | 56,962,534 | 8,476,836,113 | 0.02 | 46.35 | 98.71 | 95.78 | 52,389,959(91.97%) | 1777486(3.12%) | 50612473(88.85%) | 93.95% | 4.28% | 1.77% |

**Table S3.** DEGs related to the plant-pathogen interaction pathway of the tissue cultured seedlings of 'Rose Honey' in response to endophytes fungi Epi R2-21 and Alt XHYN2 after 6 h, 6 d, and 15 d inoculation, respectively ($|Log_2FC| \geq 1$; $p$-adjust < 0.05).

| **No.** | **gene ID** | **KO ID** | **Gene product** | **KEGG definition** | **Gene description** | **$Log_2FC$** | | | | | |
|---|---|---|---|---|---|---|---|---|---|---|---|
| | | | | | | **R2-21_6h vs Con_6h** | **R2-21_6d vs Con_6d** | **R2-21_15d vs Con_15d** | **XHYN2_6h vs Con_6h** | **XHYN2_6d vs Con_6d** | **XHYN2_15d vs Con_15d** |
| 1 | *VIT_00s0179g00280* | K13448 | CML | calcium-binding protein CML | Calmodulin-like protein 8 | 3.77 | 5.37 | 6.48 | 3.75 | 6.26 | 6.02 |
| 2 | *VIT_01s0010g02980* | K13448 | CML | calcium-binding protein CML | Probable calcium-binding protein CML31 | 5.65 | 3.27 | 1.82 | 3.60 | 3.30 | / |
| 3 | *VIT_01s0010g02970* | K13448 | CML | calcium-binding protein CML | Probable calcium-binding protein CML31 | 6.29 | 3.58 | 1.76 | 4.48 | 3.40 | / |
| 4 | *VIT_01s0010g02930* | K13448 | CML | calcium-binding protein CML | Probable calcium-binding protein CML31 | 5.86 | 4.40 | 2.14 | 4.52 | 4.55 | / |
| 5 | *VIT_01s0010g03020* | K13448 | CML | calcium-binding protein CML | Probable calcium-binding protein CML31 | 5.09 | 3.31 | 1.80 | 2.68 | 3.52 | / |
| 6 | *VIT_01s0010g02950* | K13448 | CML | calcium-binding protein CML | Probable calcium-binding protein CML31 | 7.26 | 4.75 | 2.08 | 5.99 | 4.74 | / |
| 7 | *VIT_14s0030g02150* | K13448 | CML | calcium-binding protein CML | Calmodulin-like protein 11 | 1.84 | 2.83 | 4.73 | / | 3.39 | 4.84 |
| 8 | *VIT_07s0031g00700* | K13448 | CML | calcium-binding protein CML | Caltractin | / | 2.05 | 1.28 | / | 1.83 | 1.04 |
| 9 | *VIT_08s0056g00290* | K13448 | CML | calcium-binding protein CML | Calcium-binding allergen Bet v 3 | 1.77 | 1.61 | / | 1.97 | 2.01 | / |
| 10 | *VIT_01s0011g02470* | K13448 | CML | calcium-binding protein CML | Probable calcium-binding protein CML16 | 1.96 | 1.47 | / | 1.59 | 1.46 | / |
| 11 | *VIT_01s0010g03040* | K13448 | CML | calcium-binding protein CML | Putative calcium-binding protein CML23 | 5.42 | 3.08 | 1.76 | / | 3.36 | / |
| 12 | *VIT_05s0102g00450* | K13448 | CML | calcium-binding protein CML | Calmodulin-like protein 5 | 1.17 | 2.67 | 1.68 | / | 2.84 | / |
| 13 | *VIT_01s0010g03000* | K13448 | CML | calcium-binding protein CML | Probable calcium-binding protein CML31 | 3.35 | 3.49 | 2.54 | / | 3.46 | / |
| 14 | *VIT_17s0000g01630* | K13448 | CML | calcium-binding protein CML | Putative calcium-binding protein CML19 | 5.40 | 3.44 | 1.27 | 6.16 | 4.66 | / |
| 15 | *VIT_01s0010g03010* | K13448 | CML | calcium-binding protein CML | Probable calcium-binding protein CML31 | 5.71 | 3.53 | 2.37 | 3.56 | 3.69 | / |
| 16 | *VIT_01s0010g02960* | K13448 | CML | calcium-binding protein CML | Probable calcium-binding protein CML31 | 4.91 | 3.88 | 2.03 | 3.23 | 3.68 | / |
| 17 | *VIT_01s0010g02940* | K13448 | CML | calcium-binding protein CML | Probable calcium-binding protein CML31 | 5.14 | 3.98 | 1.92 | 3.58 | 3.78 | / |
| 18 | *VIT_05s0020g04420* | K13448 | CML | calcium-binding protein CML | Calmodulin-like protein 11 | 1.51 | 3.40 | / | 3.37 | 4.25 | / |
| 19 | *VIT_18s0122g00180* | K13448 | CML | calcium-binding protein CML | Calcium-binding protein CML37 | 1.64 | 1.22 | / | / | 1.24 | / |
| 20 | *VIT_06s0080g00450* | K13448 | CML | calcium-binding protein CML | Probable calcium-binding protein CML48 | / | 2.55 | / | / | 2.75 | / |
| 21 | *VIT_04s0023g01100* | K13448 | CML | calcium-binding protein CML | Probable calcium-binding | / | 1.85 | / | / | 1.88 | / |

| | | | | | | | | | | | |
|---|---|---|---|---|---|---|---|---|---|---|---|
| | | | | | protein CML41 | | | | | | |
| 22 | *VIT_14s0006g01400* | K13448 | CML | calcium-binding protein CML | Probable calcium-binding protein CML23 | / | 2.19 | / | / | 2.55 | / |
| 23 | *VIT_18s0001g11830* | K13448 | CML | calcium-binding protein CML | Probable calcium-binding protein CML41 | / | 1.96 | / | / | 1.86 | / |
| 24 | *VIT_02s0012g02060* | K13448 | CML | calcium-binding protein CML | Probable calcium-binding protein CML18 | 1.21 | / | / | 1.91 | / | / |
| 25 | *VIT_14s0171g00150* | K13448 | CML | calcium-binding protein CML | Probable calcium-binding protein CML31 | / | / | 2.67 | / | 4.98 | / |
| 26 | *VIT_16s0039g01880* | K13448 | CML | calcium-binding protein CML | Probable calcium-binding protein CML18 | / | / | 1.20 | / | / | / |
| 27 | *VIT_05s0029g00070* | K13448 | CML | calcium-binding protein CML | Probable calcium-binding protein CML22 | / | -1.20 | / | / | / | -1.16 |
| 28 | *VIT_17s0000g04460* | K13448 | CML | calcium-binding protein CML | Probable calcium-binding protein CML15 | / | / | / | / | / | -1.39 |
| 29 | *VIT_11s0016g05740* | K13448 | CML | calcium-binding protein CML | Calmodulin-like protein 5 | / | / | / | -1.24 | / | / |
| 30 | *VIT_03s0063g00530* | K13448 | CML | calcium-binding protein CML | Calmodulin-like protein 1 | -1.44 | / | / | -1.62 | / | / |
| 31 | *VIT_03s0097g00700* | K13449 | PR1 | pathogenesis-related protein 1 | Basic form of pathogenesis-related protein 1 | 1.68 | 6.87 | 8.00 | 1.82 | 7.27 | 7.76 |
| 32 | *VIT_03s0088g00690* | K13449 | PR1 | pathogenesis-related protein 1 | Basic form of pathogenesis-related protein 1 | / | 3.47 | 5.06 | / | 2.56 | 5.27 |
| 33 | *VIT_03s0088g00700* | K13449 | PR1 | pathogenesis-related protein 1 | Basic form of pathogenesis-related protein 1 | / | 4.20 | 3.58 | / | 5.98 | 4.01 |
| 34 | *VIT_03s0088g00750* | K13449 | PR1 | pathogenesis-related protein 1 | Basic form of pathogenesis-related protein 1 | / | / | / | / | 3.47 | / |
| 35 | *VIT_03s0088g00710* | K13449 | PR1 | pathogenesis-related protein 1 | Basic form of pathogenesis-related protein 1 | / | / | 1.21 | / | -2.03 | / |
| 36 | *VIT_03s0088g00810* | K13449 | PR1 | pathogenesis-related protein 1 | Basic form of pathogenesis-related protein 1 | / | / | / | / | -3.35 | -1.72 |
| 37 | *VIT_03s0088g00780* | K13449 | PR1 | pathogenesis-related protein 1 | Basic form of pathogenesis-related protein 1 | / | / | / | / | -3.51 | -2.43 |
| 38 | *VIT_01s0011g00990* | K13457 | RPM1, RPS3 | disease resistance protein RPM1 | Disease resistance protein RPM1 | 2.36 | 5.32 | 2.76 | 2.14 | 5.59 | 1.36 |
| 39 | *VIT_07s0005g06210* | K13457 | RPM1, RPS3 | disease resistance protein RPM1 | Disease resistance protein RPM1 | 2.45 | 2.36 | 2.08 | 2.70 | 2.31 | 1.75 |
| 40 | *VIT_15s0045g00880* | K13457 | RPM1, RPS3 | disease resistance protein RPM1 | Putative disease resistance protein At1g50180 | 3.56 | 2.26 | / | 2.85 | 2.11 | / |
| 41 | *VIT_15s0045g00980* | K13457 | RPM1, RPS3 | disease resistance protein RPM1 | Putative disease resistance protein At1g50180 | 4.59 | 2.84 | / | 4.44 | 3.12 | / |
| 42 | *VIT_12s0057g01130* | K13457 | RPM1, | disease resistance protein | Disease resistance protein | 1.78 | 1.44 | / | 1.18 | 1.24 | / |

| | | | | | | | | | | | |
|---|---|---|---|---|---|---|---|---|---|---|---|
| | | | RPS3 | RPM1 | RPM1 | | | | | | |
| 43 | *VIT_03s0038g01400* | K13457 | RPM1, RPS3 | disease resistance protein RPM1 | Probable disease resistance RPP8-like protein 2 | / | 3.08 | 7.26 | / | 3.80 | 5.73 |
| 44 | *VIT_00s0515g00020* | K13457 | RPM1, RPS3 | disease resistance protein RPM1 | Probable disease resistance RPP8-like protein 4 | / | 1.58 | 1.47 | 1.26 | 1.32 | / |
| 45 | *VIT_15s0045g01020* | K13457 | RPM1, RPS3 | disease resistance protein RPM1 | Probable disease resistance RPP8-like protein 4 | / | 1.87 | 1.24 | / | 2.08 | / |
| 46 | *VIT_15s0045g00810* | K13457 | RPM1, RPS3 | disease resistance protein RPM1 | Putative disease resistance protein At1g50180 | 1.93 | 2.50 | / | / | 2.37 | / |
| 47 | *VIT_15s0046g03660* | K13457 | RPM1, RPS3 | disease resistance protein RPM1 | Disease resistance protein RPH8A | / | 3.01 | / | / | 3.04 | / |
| 48 | *VIT_15s0045g00680* | K13457 | RPM1, RPS3 | disease resistance protein RPM1 | Putative disease resistance protein At1g50180 | / | 1.86 | / | / | 2.34 | / |
| 49 | *VIT_15s0045g00920* | K13457 | RPM1, RPS3 | disease resistance protein RPM1 | Putative disease resistance protein At1g50180 | / | 2.67 | / | / | 2.91 | / |
| 50 | *VIT_15s0045g00640* | K13457 | RPM1, RPS3 | disease resistance protein RPM1 | Putative disease resistance protein At1g50180 | / | 5.03 | / | / | / | / |
| 51 | *VIT_01s0011g01000* | K13457 | RPM1, RPS3 | disease resistance protein RPM1 | Disease resistance protein RPM1 | / | / | / | / | 1.13 | / |
| 52 | *VIT_03s0038g01530* | K13457 | RPM1, RPS3 | disease resistance protein RPM1 | Putative disease resistance protein At1g50180 | / | / | / | / | / | 1.08 |
| 53 | *VIT_05s0020g04930* | K13457 | RPM1, RPS3 | disease resistance protein RPM1 | Disease resistance protein RPM1 | 3.37 | / | / | / | / | / |
| 54 | *VIT_15s0046g02810* | K13457 | RPM1, RPS3 | disease resistance protein RPM1 | Putative disease resistance protein At1g58400 | / | 1.53 | -4.77 | / | 1.96 | -1.97 |
| 55 | *VIT_09s0096g00930* | K13457 | RPM1, RPS3 | disease resistance protein RPM1 | Putative disease resistance protein At1g50180 | / | / | -6.63 | / | 2.92 | / |
| 56 | *VIT_03s0038g01770* | K13457 | RPM1, RPS3 | disease resistance protein RPM1 | Putative disease resistance protein At1g50180 | / | / | -2.52 | / | 1.50 | / |
| 57 | *VIT_05s0020g04940* | K13457 | RPM1, RPS3 | disease resistance protein RPM1 | unnamed protein product | / | / | / | / | / | -1.18 |
| 58 | *VIT_06s0009g01360* | K13457 | RPM1, RPS3 | disease resistance protein RPM1 | Disease resistance protein RPM1 | / | / | -1.04 | / | / | / |
| 59 | *VIT_07s0005g06200* | K13457 | RPM1, RPS3 | disease resistance protein RPM1 | Putative disease resistance RPP13-like protein 3 | / | / | -1.87 | / | / | -1.08 |
| 60 | *VIT_15s0046g02750* | K13457 | RPM1, RPS3 | disease resistance protein RPM1 | Putative disease resistance protein At1g50180 | / | -2.79 | -1.59 | -1.47 | -3.20 | / |
| 61 | *VIT_03s0038g01670* | K13457 | RPM1, RPS3 | disease resistance protein RPM1 | Putative disease resistance protein At1g50180 | / | / | -1.25 | / | / | / |
| 62 | *VIT_16s0022g01330* | K13457 | RPM1, RPS3 | disease resistance protein RPM1 | Putative disease resistance RPP13-like protein 3 | / | -2.13 | / | / | -2.71 | / |
| 63 | *VIT_01s0011g01040* | K13457 | RPM1, RPS3 | disease resistance protein RPM1 | Putative disease resistance RPP13-like protein 3 | / | / | / | -5.00 | / | / |
| 64 | *VIT_13s0067g00830* | K13457 | RPM1, | disease resistance protein | Putative disease resistance | / | / | -1.92 | / | / | / |

| | | | | | | | | | | | |
|---|---|---|---|---|---|---|---|---|---|---|---|
| | | | RPS3 | RPM1 | RPP13-like protein 3 | | | | | | |
| 65 | *VIT_03s0038g01620* | K13457 | RPM1, RPS3 | disease resistance protein RPM1 | Putative disease resistance protein At1g50180 | -2.08 | / | -1.39 | / | / | / |
| 66 | *VIT_03s0038g01520* | K13457 | RPM1, RPS3 | disease resistance protein RPM1 | Disease resistance RPP8-like protein 3 | / | / | / | / | -1.13 | / |
| 67 | *VIT_03s0038g01550* | K13457 | RPM1, RPS3 | disease resistance protein RPM1 | Disease resistance protein RPP8 | / | / | -5.96 | / | / | / |
| 68 | *VIT_15s0046g02820* | K13457 | RPM1, RPS3 | disease resistance protein RPM1 | Probable disease resistance protein RXW24L | / | -1.79 | -1.53 | / | -1.73 | / |
| 69 | *VIT_14s0060g02320* | K13447 | RBOH | respiratory burst oxidase [EC:1.6.3.- 1.11.1.-] | Respiratory burst oxidase homolog protein B | 2.27 | 3.72 | 2.52 | 3.27 | 4.21 | 1.51 |
| 70 | *VIT_01s0150g00440* | K13447 | RBOH | respiratory burst oxidase [EC:1.6.3.- 1.11.1.-] | Respiratory burst oxidase homolog protein D | 2.86 | 4.32 | 1.41 | 4.34 | 5.29 | / |
| 71 | *VIT_02s0025g00510* | K13447 | RBOH | respiratory burst oxidase [EC:1.6.3.- 1.11.1.-] | Respiratory burst oxidase homolog protein A | / | 1.59 | 1.57 | 1.01 | 1.91 | 1.69 |
| 72 | *VIT_19s0014g02830* | K13447 | RBOH | respiratory burst oxidase [EC:1.6.3.- 1.11.1.-] | Respiratory burst oxidase homolog protein C | / | / | / | -1.78 | -1.04 | / |
| 73 | *VIT_10s0003g02910* | K13420 | FLS2 | LRR receptor-like serine/threonine-protein kinase FLS2 [EC:2.7.11.1] | LRR receptor-like serine/threonine-protein kinase FLS2 | / | / | 1.42 | 1.06 | 1.44 | / |
| 74 | *VIT_10s0003g02930* | K13420 | FLS2 | LRR receptor-like serine/threonine-protein kinase FLS2 [EC:2.7.11.1] | LRR receptor-like serine/threonine-protein kinase FLS2 | / | / | 1.56 | / | / | 1.30 |
| 75 | *VIT_12s0142g00640* | K13420 | FLS2 | LRR receptor-like serine/threonine-protein kinase FLS2 [EC:2.7.11.1] | LRR receptor-like serine/threonine-protein kinase FLS2 | / | -1.59 | -1.76 | / | -1.01 | / |
| 76 | *VIT_13s0047g00260* | K13412 | CPK | calcium-dependent protein kinase [EC:2.7.11.1] | Calcium and calcium/calmodulin-dependent serine/threonine-protein kinase DMI-3 | / | 1.51 | 1.81 | 1.32 | 1.39 | 1.70 |
| 77 | *VIT_08s0007g08300* | K13412 | CPK | calcium-dependent protein kinase [EC:2.7.11.1] | Calcium-dependent protein kinase 32 | / | 2.01 | / | / | 2.42 | / |
| 78 | *VIT_03s0038g03960* | K13412 | CPK | calcium-dependent protein kinase [EC:2.7.11.1] | Calcium-dependent protein kinase 4 | / | 1.76 | / | / | 2.03 | / |
| 79 | *VIT_04s0023g03420* | K13412 | CPK | calcium-dependent protein kinase [EC:2.7.11.1] | Calcium-dependent protein kinase 28 | / | 1.61 | / | / | 1.94 | / |
| 80 | *VIT_08s0032g00780* | K13412 | CPK | calcium-dependent protein kinase [EC:2.7.11.1] | Calcium-dependent protein kinase 20 | / | / | / | / | 1.08 | / |
| 81 | *VIT_18s0001g00990* | K13412 | CPK | calcium-dependent protein kinase [EC:2.7.11.1] | Calcium-dependent protein kinase 29 | / | / | / | -1.19 | -1.46 | / |
| 82 | *VIT_09s0002g06260* | K13459 | RPS2 | disease resistance protein RPS2 | Probable disease resistance protein At5g63020 | / | 1.11 | 1.17 | / | 1.13 | / |
| 83 | *VIT_09s0002g05840* | K13459 | RPS2 | disease resistance protein RPS2 | Disease resistance protein SUMM2 | / | 1.32 | 1.35 | / | / | / |

| | | | | | | | | | | | |
|---|---|---|---|---|---|---|---|---|---|---|---|
| 84 | *VIT_14s0036g00030* | K13459 | RPS2 | disease resistance protein RPS2 | Disease resistance protein At4g27190 | / | 2.55 | / | / | / | / |
| 85 | *VIT_19s0014g00600* | K13459 | RPS2 | disease resistance protein RPS2 | Disease resistance protein At4g27190 | / | / | 1.39 | / | / | 1.33 |
| 86 | *VIT_09s0096g00420* | K13459 | RPS2 | disease resistance protein RPS2 | Probable disease resistance protein At5g63020 | / | / | 1.31 | / | / | / |
| 87 | *VIT_11s0052g00210* | K13459 | RPS2 | disease resistance protein RPS2 | Disease resistance protein At4g27190 | / | / | 1.05 | / | / | / |
| 88 | *VIT_11s0052g00200* | K13459 | RPS2 | disease resistance protein RPS2 | Probable disease resistance protein At4g27220 | / | 1.03 | / | / | / | / |
| 89 | *VIT_12s0035g01280* | K13459 | RPS2 | disease resistance protein RPS2 | Disease resistance protein SUMM2 | -1.74 | / | -2.69 | / | 1.99 | / |
| 90 | *VIT_10s0042g00460* | K13459 | RPS2 | disease resistance protein RPS2 | Probable disease resistance protein At4g27220 | -1.80 | / | -1.74 | -1.70 | 1.49 | / |
| 91 | *VIT_19s0014g00900* | K13459 | RPS2 | disease resistance protein RPS2 | Probable disease resistance protein At4g27220 | -1.14 | / | -1.38 | -1.36 | / | -1.12 |
| 92 | *VIT_19s0090g00260* | K13459 | RPS2 | disease resistance protein RPS2 | Disease resistance protein At4g27190 | / | / | / | / | -1.08 | / |
| 93 | *VIT_19s0090g00240* | K13459 | RPS2 | disease resistance protein RPS2 | Disease resistance protein At4g27190 | / | / | / | / | -1.52 | -2.06 |
| 94 | *VIT_19s0027g01740* | K13459 | RPS2 | disease resistance protein RPS2 | Probable disease resistance protein At5g63020 | -1.21 | / | / | -1.51 | -1.18 | / |
| 95 | *VIT_14s0036g00010* | K13459 | RPS2 | disease resistance protein RPS2 | Disease resistance protein At4g27190 | / | / | -1.62 | / | / | -1.01 |
| 96 | *VIT_11s0052g00270* | K13459 | RPS2 | disease resistance protein RPS2 | Probable disease resistance protein At4g27220 | / | / | -2.53 | / | / | -1.65 |
| 97 | *VIT_19s0027g01750* | K13459 | RPS2 | disease resistance protein RPS2 | Disease resistance protein SUMM2 | -1.07 | -1.04 | / | -1.20 | -1.47 | / |
| 98 | *VIT_14s0036g01110* | K13459 | RPS2 | disease resistance protein RPS2 | Probable disease resistance protein At4g27220 | / | -1.30 | / | / | -1.28 | / |
| 99 | *VIT_09s0002g05870* | K13459 | RPS2 | disease resistance protein RPS2 | Disease resistance protein SUMM2 | -1.38 | / | / | -1.36 | / | / |
| 100 | *VIT_09s0002g05000* | K13459 | RPS2 | disease resistance protein RPS2 | Probable disease resistance protein At4g14610 | / | / | / | / | -1.36 | / |
| 101 | *VIT_09s0002g04940* | K13459 | RPS2 | disease resistance protein RPS2 | Disease resistance protein SUMM2 | / | / | / | / | -1.16 | / |
| 102 | *VIT_19s0014g00570* | K13459 | RPS2 | disease resistance protein RPS2 | Probable disease resistance protein At4g27220 | / | / | -1.46 | -1.14 | / | / |

| | | | | | | | | | | | |
|---|---|---|---|---|---|---|---|---|---|---|---|
| 103 | *VIT_19s0027g01700* | K13459 | RPS2 | disease resistance protein RPS2 | Probable disease resistance protein At5g63020 | -3.68 | / | / | -5.35 | / | / |
| 104 | *VIT_09s0002g05070* | K13459 | RPS2 | disease resistance protein RPS2 | Disease resistance protein SUMM2 | -1.01 | -1.36 | -1.06 | -1.10 | -1.05 | / |
| 105 | *VIT_09s0002g05220* | K13459 | RPS2 | disease resistance protein RPS2 | Probable disease resistance protein At1g61190 | / | -1.05 | / | / | -1.46 | / |
| 106 | *VIT_09s0002g06240* | K13459; K20599 | RPS2;SUMM2 | disease resistance protein RPS2; NB-LRR protein SUMM2 | Probable disease resistance protein At5g63020 | / | 1.37 | 1.21 | / | 1.22 | 1.66 |
| 107 | *VIT_09s0002g06180* | K13459; K20599 | RPS2;SUMM2 | disease resistance protein RPS2; NB-LRR protein SUMM2 | unnamed protein product, partial | / | 1.11 | 1.78 | / | 1.59 | / |
| 108 | *VIT_09s0018g00480* | K13459; K20599 | RPS2;SUMM2 | disease resistance protein RPS2; NB-LRR protein SUMM2 | Probable disease resistance protein At5g63020 | / | 1.60 | 2.50 | / | / | / |
| 109 | *VIT_17s0000g07420* | K18875 | EDS1 | enhanced disease susceptibility 1 protein | Protein EDS1 | 1.37 | 2.05 | / | / | 2.91 | / |
| 110 | *VIT_17s0000g07400* | K18875 | EDS1 | enhanced disease susceptibility 1 protein | Protein EDS1L | 1.38 | 2.04 | / | / | 2.54 | / |
| 111 | *VIT_17s0000g07370* | K18875 | EDS1 | enhanced disease susceptibility 1 protein | Protein EDS1 | / | 2.24 | / | / | 3.48 | / |
| 112 | *VIT_14s0108g01000* | K02183 | CALM | calmodulin | Probable calcium-binding protein CML45 | 1.44 | 1.24 | / | / | 1.32 | / |
| 113 | *VIT_08s0040g00470* | K02183 | CALM | calmodulin | Calmodulin-1 | / | / | / | / | 1.02 | / |
| 114 | *VIT_05s0077g00810* | K02183 | CALM | calmodulin | Calcium-binding protein CP1 | / | / | / | / | / | -1.19 |
| 115 | *VIT_05s0077g00190* | K02183 | CALM | calmodulin | Calcium-binding protein CP1 | / | / | / | / | / | -1.37 |
| 116 | *VIT_17s0053g00860* | K05391 | CNGC | cyclic nucleotide gated channel, plant | Probable cyclic nucleotide-gated ion channel 20, chloroplastic | / | / | 2.21 | / | 2.08 | 2.11 |
| 117 | *VIT_14s0108g01420* | K05391 | CNGC | cyclic nucleotide gated channel, plant | Cyclic nucleotide-gated ion channel 2 | / | 1.17 | / | / | 1.09 | / |
| 118 | *VIT_08s0040g01770* | K05391 | CNGC | cyclic nucleotide gated channel, plant | Protein CNGC15c | / | / | / | / | 1.09 | / |
| 119 | *VIT_15s0046g00980* | K05391 | CNGC | cyclic nucleotide gated channel, plant | Cyclic nucleotide-gated ion channel 1 | -1.03 | / | -1.10 | -1.33 | / | -1.25 |
| 120 | *VIT_09s0018g00410* | K05391 | CNGC | cyclic nucleotide gated channel, plant | Putative cyclic nucleotide-gated ion channel 8 | / | / | -1.90 | / | / | / |
| 121 | *VIT_00s0231g00040* | K13456 | RIN4 | RPM1-interacting protein 4 | RPM1-interacting protein 4 | / | / | 1.09 | / | / | 1.15 |
| 122 | *VIT_05s0029g00690* | K13456 | RIN4 | RPM1-interacting protein 4 | RPM1-interacting protein 4 | / | 1.21 | / | / | 1.49 | -1.15 |
| 123 | *VIT_00s0516g00010* | K13456 | RIN4 | RPM1-interacting protein 4 | NOI-like protein | / | / | -1.40 | / | 1.33 | / |

| | | | | | | | | | | | |
|---|---|---|---|---|---|---|---|---|---|---|---|
| 124 | *VIT_08s0058g00690* | K13424 | WRKY33 | WRKY transcription factor 33 | Probable WRKY transcription factor 33 | 2.69 | 2.98 | / | 2.80 | 3.54 | / |
| 125 | *VIT_06s0004g07500* | K13424 | WRKY33 | WRKY transcription factor 33 | WRKY transcription factor WRKY24 | 1.05 | 1.49 | / | 1.14 | 1.67 | / |
| 126 | *VIT_02s0025g00420* | K13425 | WRKY22 | WRKY transcription factor 22 | WRKY transcription factor 22 | 3.03 | 4.14 | 3.22 | 3.65 | 4.56 | 2.13 |
| 127 | *VIT_15s0046g02190* | K13425 | WRKY22 | WRKY transcription factor 22 | WRKY transcription factor 22 | 1.37 | 1.30 | / | 1.48 | 1.53 | / |
| 128 | *VIT_18s0001g14500* | K09487 | HSP90B, TRA1 | heat shock protein 90kDa beta | Endoplasmin homolog | 1.01 | 1.14 | 2.10 | 1.56 | / | 1.98 |
| 129 | *VIT_01s0010g00680* | K09487 | HSP90B, TRA1 | heat shock protein 90kDa beta | Heat shock protein 90-5, chloroplastic | / | / | -1.16 | / | / | -1.31 |
| 130 | *VIT_05s0062g00690* | K04079 | HSP90A, htpG | heat shock protein 90kDa beta | Heat shock protein 83 | 3.73 | 3.26 | 7.78 | 3.98 | 3.53 | 6.43 |
| 131 | *VIT_02s0025g00280* | K04079 | HSP90A, htpG | heat shock protein 90kDa beta | Heat shock protein 83 | / | / | / | 1.97 | / | / |
| 132 | *VIT_16s0050g01150* | K04079 | HSP90A, htpG | heat shock protein 90kDa beta | Heat shock protein 83 | / | / | -1.10 | / | / | / |
| 133 | *VIT_08s0032g01220* | K13412 | CPK25 | calcium-dependent protein kinase [EC:2.7.11.1] | Calcium-dependent protein kinase 1 | 1.50 | 5.00 | / | 1.87 | 5.66 | / |
| 134 | *VIT_01s0146g00250* | K13430 | PBS1 | serine/threonine-protein kinase PBS1 [EC:2.7.11.1] | Serine/threonine-protein kinase PBS1 | / | 1.27 | / | / | 1.05 | / |
| 135 | *VIT_11s0016g02970* | K04368 | MAP2K1 , MEK1 | mitogen-activated protein kinase kinase 1 [EC:2.7.12.2] | Mitogen-activated protein kinase kinase 6 | / | / | 2.26 | / | / | 2.30 |
| 136 | *VIT_06s0004g03540* | K20536 | MPK3 | mitogen-activated protein kinase 3 [EC:2.7.11.24] | Mitogen-activated protein kinase 3 | / | 1.82 | / | 1.08 | 2.31 | / |
| 137 | *VIT_09s0018g01820* | K13413 | MKK4_5 | mitogen-activated protein kinase kinase 4/5 [EC:2.7.12.2] | Mitogen-activated protein kinase kinase 5 | / | 1.05 | / | / | / | / |
| 139 | *VIT_06s0004g08190* | K13434 | PTI6 | Pathogenesis-related genes transcriptional activator PTI6 | pathogenesis-related genes transcriptional activator PTI6 | / | -1.35 | -1.26 | / | -1.39 | -1.18 |
| 138 | *VIT_04s0023g00470* | K18835 | WRKY2 | WRKY transcription factor 2 | Probable WRKY transcription factor 2 | / | -1.05 | -2.83 | / | / | -1.83 |
| 140 | *VIT_10s0116g00910* | K13429 | CERK1 | chitin elicitor receptor kinase 1 | LysM domain receptor-like kinase 3 | / | / | / | / | -1.26 | / |

**Table S4.** DEGs related to the plant hormone signal transduction pathway of the tissue cultured seedlings of 'Rose Honey' in response to endophytes fungi Epi R2-21 and Alt XHYN2 after 6 h, 6 d, and 15 d inoculation, respectively ($|Log_2FC| \geq 1$; *p*-adjust < 0.05).

| No. | gene ID | KO ID | Gene product | KEGG definition | Gene description | $Log_2FC$ | | | | | |
|---|---|---|---|---|---|---|---|---|---|---|---|
| | | | | | | R2-21_6h vs Con_6h | R2-21_6d vs Con_6d | R2-21_15d vs Con_15d | XHYN2_6h vs Con_6h | XHYN2_6d vs Con_6d | XHYN2_15d vs Con_15d |
| 1 | *VIT_19s0014g04690* | K14487 | GH3 | auxin responsive GH3 gene family | Indole-3-acetic acid-amido synthetase GH3.6 | 2.40 | 4.44 | 7.02 | 2.36 | 3.82 | 5.85 |
| 2 | *VIT_07s0129g00660* | K14487 | GH3 | auxin responsive GH3 gene family | Probable indole-3-acetic acid-amido synthetase GH3.1 | 2.44 | 3.60 | 8.77 | 5.24 | 2.98 | 6.63 |
| 3 | *VIT_03s0091g00310* | K14487 | GH3 | auxin responsive GH3 gene family | Probable indole-3-acetic acid-amido synthetase GH3.1 | / | 1.31 | 4.25 | / | 2.11 | 2.90 |
| 4 | *VIT_07s0104g00800* | K14487 | GH3 | auxin responsive GH3 gene family | Probable indole-3-acetic acid-amido synthetase GH3.6 | / | / | 1.05 | / | / | / |
| 5 | *VIT_12s0059g01870* | K14487 | GH3 | auxin responsive GH3 gene family | Indole-3-acetic acid-amido synthetase GH3.10 | / | -1.22 | / | -1.98 | -1.57 | / |
| 6 | *VIT_01s0150g00300* | K14487 | GH3 | auxin responsive GH3 gene family | Indole-3-acetic acid-amido synthetase GH3.17 | / | / | -5.87 | / | / | / |
| 7 | *VIT_19s0085g00010* | K14488 | SAUR | SAUR family protein | Auxin-responsive protein SAUR72 | 1.55 | 2.64 | / | 1.95 | 2.60 | 2.23 |
| 8 | *VIT_15s0048g00530* | K14488 | SAUR | SAUR family protein | Auxin-responsive protein SAUR36 | 1.37 | 1.91 | / | 1.27 | 2.37 | / |
| 9 | *VIT_16s0098g01150* | K14488 | SAUR | SAUR family protein | Auxin-responsive protein SAUR32 | / | 2.13 | 1.23 | 1.51 | 2.46 | / |
| 10 | *VIT_11s0016g00520* | K14488 | SAUR | SAUR family protein | Auxin-induced protein 15A | / | 2.34 | 3.81 | / | 2.59 | 3.62 |
| 11 | *VIT_08s0058g01160* | K14488 | SAUR | SAUR family protein | indole-3-acetic acid-induced protein ARG7-like | / | 2.60 | / | / | 3.82 | / |
| 12 | *VIT_12s0028g00690* | K14488 | SAUR | SAUR family protein | Auxin-responsive protein SAUR71 | / | / | / | / | 2.74 | 4.26 |
| 13 | *VIT_01s0146g00180* | K14488 | SAUR | SAUR family protein | Auxin-responsive protein SAUR32 | / | / | / | / | / | 1.03 |
| 14 | *VIT_09s0002g00670* | K14488 | SAUR | SAUR family protein | Auxin-responsive protein SAUR71 | / | / | / | / | / | 1.97 |
| 15 | *VIT_02s0154g00010* | K14488 | SAUR | SAUR family protein | Auxin-responsive protein SAUR36 | 1.18 | / | / | / | / | / |
| 16 | *VIT_01s0146g00210* | K14488 | SAUR | SAUR family protein | auxin-responsive protein SAUR71 | / | / | 1.96 | / | / | / |
| 17 | *VIT_04s0023g03230* | K14488 | SAUR | SAUR family protein | Auxin-responsive protein SAUR50 | / | -2.73 | -1.11 | / | -2.86 | / |
| 18 | *VIT_03s0038g00950* | K14488 | SAUR | SAUR family protein | Auxin-responsive protein SAUR50 | / | -3.67 | / | / | -4.29 | / |

| | | | | | | | | | | | |
|---|---|---|---|---|---|---|---|---|---|---|---|
| 19 | *VIT_15s0048g02860* | K14488 | SAUR | SAUR family protein | Auxin-responsive protein SAUR32 | / | -1.14 | / | / | -1.18 | / |
| 20 | *VIT_03s0038g01110* | K14488 | SAUR | SAUR family protein | Auxin-responsive protein SAUR20 | / | -5.24 | / | / | / | / |
| 21 | *VIT_03s0038g01130* | K14488 | SAUR | SAUR family protein | Auxin-responsive protein SAUR20 | / | -5.81 | / | / | / | / |
| 22 | *VIT_03s0038g01150* | K14488 | SAUR | SAUR family protein | Auxin-responsive protein SAUR21 | / | -4.80 | / | / | / | / |
| 23 | *VIT_09s0002g00890* | K13464 | JAZ | jasmonate ZIM domain-containing protein | Protein TIFY 10A | / | 2.40 | 2.46 | 1.85 | 2.73 | 2.68 |
| 24 | *VIT_01s0146g00480* | K13464 | JAZ | jasmonate ZIM domain-containing protein | Protein TIFY 9 | / | 4.11 | 5.77 | 3.76 | 4.65 | 5.61 |
| 25 | *VIT_11s0016g00710* | K13464 | JAZ | jasmonate ZIM domain-containing protein | Protein TIFY 10A | 1.68 | 2.14 | / | 1.89 | 2.67 | / |
| 26 | *VIT_01s0011g05560* | K13464 | JAZ | jasmonate ZIM domain-containing protein | Protein TIFY 6B | / | / | 1.08 | / | / | 1.18 |
| 27 | *VIT_17s0000g02230* | K13464 | JAZ | jasmonate ZIM domain-containing protein | Protein TIFY 6B | / | -1.57 | -1.69 | / | -1.38 | -1.43 |
| 28 | *VIT_11s0016g04490* | K14484 | IAA | auxin-responsive protein IAA | Auxin-responsive protein IAA17 | / | 1.98 | / | / | 2.27 | / |
| 29 | *VIT_09s0002g05150* | K14484 | IAA | auxin-responsive protein IAA | Auxin-induced protein 22A | / | / | / | 2.87 | / | / |
| 30 | *VIT_09s0002g03410* | K14484 | IAA | auxin-responsive protein IAA | Auxin-responsive protein IAA26 | / | / | / | / | / | 1.22 |
| 31 | *VIT_14s0030g02310* | K14484 | IAA | auxin-responsive protein IAA | Auxin-induced protein 22D | / | -3.74 | -2.42 | / | -2.00 | -1.26 |
| 32 | *VIT_07s0141g00290* | K14484 | IAA | auxin-responsive protein IAA | Auxin-responsive protein IAA16 | / | -2.42 | -3.19 | / | -1.38 | -1.91 |
| 33 | *VIT_05s0020g04680* | K14484 | IAA | auxin-responsive protein IAA | Auxin-induced protein 22D | / | -1.28 | / | / | / | / |
| 34 | *VIT_14s0081g00010* | K14484 | IAA | auxin-responsive protein IAA | Auxin-responsive protein IAA16 | / | -1.43 | -2.30 | / | / | -1.47 |
| 35 | *VIT_07s0141g00270* | K14484 | IAA | auxin-responsive protein IAA | Auxin-induced protein 22D | / | -1.19 | -2.56 | / | / | -2.47 |
| 36 | *VIT_11s0016g05640* | K14484 | IAA | auxin-responsive protein IAA | auxin-responsive protein IAA9 | / | -2.94 | / | / | -3.61 | / |
| 37 | *VIT_04s0008g05560* | K14484 | IAA | auxin-responsive protein IAA | Auxin-responsive protein IAA29 | / | -1.50 | / | / | / | -1.66 |
| 38 | *VIT_05s0020g04690* | K14484 | IAA | auxin-responsive protein IAA | Auxin-responsive protein IAA7 | / | / | -1.79 | / | / | -1.66 |
| 39 | *VIT_09s0002g04080* | K14484 | IAA | auxin-responsive protein IAA | Auxin-responsive protein IAA27 | / | -1.37 | -3.40 | / | / | -2.58 |
| 40 | *VIT_11s0016g03540* | K14484 | IAA | auxin-responsive protein IAA | Auxin-responsive protein IAA27 | / | / | -2.06 | / | / | -1.91 |
| 41 | *VIT_04s0008g00220* | K14484 | IAA | auxin-responsive protein IAA | Auxin-responsive protein IAA26 | / | / | / | -1.13 | / | / |
| 42 | VIT_03s0097g00700 | K13449 | PR1 | pathogenesis-related protein 1 | Basic form of | 1.68 | 6.87 | 8.00 | 1.82 | 7.27 | 7.76 |

| | | | | | | | | | | | |
|---|---|---|---|---|---|---|---|---|---|---|---|
| | | | | | pathogenesis-related protein 1 | | | | | | |
| 43 | VIT_03s0088g00690 | K13449 | PR1 | pathogenesis-related protein 1 | Basic form of pathogenesis-related protein 1 | / | 3.47 | 5.06 | / | 2.56 | 5.27 |
| 44 | VIT_03s0088g00700 | K13449 | PR1 | pathogenesis-related protein 1 | Basic form of pathogenesis-related protein 1 | / | 4.20 | 3.58 | / | 5.98 | 4.01 |
| 45 | VIT_03s0088g00750 | K13449 | PR1 | pathogenesis-related protein 1 | Basic form of pathogenesis-related protein 1 | / | / | / | / | 3.47 | / |
| 46 | VIT_03s0088g00710 | K13449 | PR1 | pathogenesis-related protein 1 | Basic form of pathogenesis-related protein 1 | / | / | 1.21 | / | -2.03 | / |
| 47 | VIT_03s0088g00810 | K13449 | PR1 | pathogenesis-related protein 1 | Basic form of pathogenesis-related protein 1 | / | / | / | / | -3.35 | -1.72 |
| 48 | VIT_03s0088g00780 | K13449 | PR1 | pathogenesis-related protein 1 | Basic form of pathogenesis-related protein 1 | / | / | / | / | -3.51 | -2.43 |
| 49 | *VIT_13s0067g03070* | K14492 | ARR-A | two-component response regulator ARR-A family | Two-component response regulator ARR17 | 1.33 | 1.86 | 1.57 | / | 1.92 | 1.24 |
| 50 | *VIT_01s0026g00940* | K14492 | ARR-A | two-component response regulator ARR-A family | Two-component response regulator ARR4 | / | 2.23 | / | / | 4.05 | / |
| 51 | *VIT_17s0000g07580* | K14492 | ARR-A | two-component response regulator ARR-A family | Two-component response regulator ARR6 | / | / | / | 1.46 | / | / |
| 52 | *VIT_13s0067g03430* | K14492 | ARR-A | two-component response regulator ARR-A family | Two-component response regulator ORR9 | / | / | / | / | 1.23 | / |
| 53 | *VIT_13s0067g03510* | K14492 | ARR-A | two-component response regulator ARR-A family | Two-component response regulator ORR9 | / | / | / | / | -1.48 | / |
| 54 | *VIT_13s0067g03490* | K14492 | ARR-A | two-component response regulator ARR-A family | Two-component response regulator ORR9 | / | / | -1.06 | / | / | / |
| 55 | *VIT_11s0052g01280* | K14504 | TCH4 | xyloglucan:xyloglucosyl transferase TCH4 [EC:2.4.1.207] | Probable xyloglucan endotransglucosylase/hydrolase protein 23 | / | 4.91 | 1.21 | / | 5.37 | / |
| 56 | *VIT_11s0052g01180* | K14504 | TCH4 | xyloglucan:xyloglucosyl transferase TCH4 [EC:2.4.1.207] | Probable xyloglucan endotransglucosylase/hydrolase protein 23 | / | 2.76 | / | / | 3.09 | / |
| 57 | *VIT_11s0052g01190* | K14504 | TCH4 | xyloglucan:xyloglucosyl transferase TCH4 [EC:2.4.1.207] | Probable xyloglucan endotransglucosylase/hydrolase protein 23 | / | 2.63 | / | / | 2.94 | / |
| 58 | *VIT_11s0052g01330* | K14504 | TCH4 | xyloglucan:xyloglucosyl transferase TCH4 [EC:2.4.1.207] | Probable xyloglucan endotransglucosylase/hydrolase protein 23 | / | 4.58 | 1.20 | / | 4.79 | / |
| 59 | *VIT_11s0052g01310* | K14504 | TCH4 | xyloglucan:xyloglucosyl transferase TCH4 [EC:2.4.1.207] | Xyloglucan endotransglucosylase/hydrolase 2 | / | 3.59 | / | / | 3.90 | / |

| | | | | | | | | | | | |
|---|---|---|---|---|---|---|---|---|---|---|---|
| 60 | *VIT_11s0052g01220* | K14504 | TCH4 | xyloglucan:xyloglucosyl transferase TCH4 [EC:2.4.1.207] | Probable xyloglucan endotransglucosylase/hydrolase protein 23 | / | 1.94 | / | / | 2.10 | / |
| 61 | *VIT_11s0052g01340* | K14504 | TCH4 | xyloglucan:xyloglucosyl transferase TCH4 [EC:2.4.1.207] | Xyloglucan endotransglucosylase/hydrolase 2 | / | 2.62 | / | / | 3.26 | / |
| 62 | *VIT_11s0052g01230* | K14504 | TCH4 | xyloglucan:xyloglucosyl transferase TCH4 [EC:2.4.1.207] | Probable xyloglucan endotransglucosylase/hydrolase protein 25 | / | 2.22 | / | / | / | / |
| 63 | *VIT_11s0052g01210* | K14504 | TCH4 | xyloglucan:xyloglucosyl transferase TCH4 [EC:2.4.1.207] | Probable xyloglucan endotransglucosylase/hydrolase protein 23 | / | 5.71 | / | / | / | / |
| 64 | *VIT_11s0052g01200* | K14504 | TCH4 | xyloglucan:xyloglucosyl transferase TCH4 [EC:2.4.1.207] | Probable xyloglucan endotransglucosylase/hydrolase protein 23 | / | 2.49 | / | / | 2.68 | -1.15 |
| 65 | *VIT_11s0052g01250* | K14504 | TCH4 | xyloglucan:xyloglucosyl transferase TCH4 [EC:2.4.1.207] | Xyloglucan endotransglucosylase/hydrolase 2 | -2.17 | / | / | -1.85 | / | / |
| 66 | *VIT_18s0001g13930* | K14486 | ARF | auxin response factor | Auxin response factor 5 | / | 1.21 | 1.56 | / | / | 1.49 |
| 67 | *VIT_07s0104g01230* | K14486 | ARF | auxin response factor | Auxin response factor 2 | / | / | 4.70 | / | 2.65 | 5.64 |
| 68 | *VIT_15s0046g00290* | K14486 | ARF | auxin response factor | Auxin response factor 7 | -1.73 | / | -2.36 | -1.96 | / | / |
| 69 | *VIT_02s0025g01740* | K14486 | ARF | auxin response factor | Auxin response factor 7 | -1.56 | -1.10 | -1.42 | -1.73 | / | / |
| 70 | *VIT_10s0003g00420* | K14486 | ARF | auxin response factor | Auxin response factor 3 | / | / | -1.27 | -1.01 | -1.50 | -1.68 |
| 71 | *VIT_06s0004g05810* | K14500 | BSK | BR-signaling kinase [EC:2.7.11.1] | Serine/threonine-protein kinase BSK5 | / | / | 1.57 | / | / | 1.37 |
| 72 | *VIT_03s0038g03270* | K14500 | BSK | BR-signaling kinase [EC:2.7.11.1] | Serine/threonine-protein kinase BSK1 | / | / | 1.11 | / | / | 1.06 |
| 73 | *VIT_18s0001g00180* | K14500 | BSK | BR-signaling kinase [EC:2.7.11.1] | Serine/threonine-protein kinase BSK1 | / | / | 1.14 | / | / | / |
| 74 | *VIT_02s0012g01140* | K14500 | BSK | BR-signaling kinase [EC:2.7.11.1] | Serine/threonine-protein kinase BSK2 | -2.04 | / | -5.52 | / | / | -2.92 |
| 75 | *VIT_07s0005g03230* | K14516 | ERF1 | ethylene-responsive transcription factor 1 | Ethylene-responsive transcription factor 1B | / | / | 5.23 | / | / | 6.09 |
| 76 | *VIT_05s0049g00510* | K14516 | ERF1 | ethylene-responsive transcription factor 1 | Ethylene-responsive transcription factor 1B | / | / | 2.91 | / | / | 2.83 |
| 77 | *VIT_07s0005g03260* | K14516 | ERF1 | ethylene-responsive transcription factor 1 | Ethylene-responsive transcription factor 1B | / | -2.16 | 1.35 | / | -2.33 | 2.40 |
| 78 | *VIT_05s0049g00090* | K14509 | ETR, ERS | ethylene receptor [EC:2.7.13.-] | Ethylene receptor 2 | / | / | 1.85 | / | / | 1.10 |
| 79 | *VIT_06s0004g05240* | K14509 | ETR, ERS | ethylene receptor [EC:2.7.13.-] | Ethylene receptor 2 | / | / | 2.78 | / | / | / |
| 80 | *VIT_12s0055g00420* | K14432 | ABF | ABA responsive element binding factor | bZIP transcription factor 46 | / | / | 1.59 | / | / | 1.37 |
| 81 | *VIT_04s0069g01150* | K14432 | ABF | ABA responsive element binding factor | ABSCISIC ACID-INSENSITIVE 5-like protein 2 | / | / | / | / | / | 1.85 |
| 82 | *VIT_03s0063g00310* | K14432 | ABF | ABA responsive element | ABSCISIC | / | / | / | / | -1.10 | / |

| | | | | | | | | | | | |
|---|---|---|---|---|---|---|---|---|---|---|---|
| | | | | binding factor | ACID-INSENSITIVE 5-like protein 7 | | | | | | |
| 83 | *VIT_18s0001g03540* | K13946 | AUX1 | auxin influx carrier (AUX1 LAX family) | Auxin transporter-like protein 3 | / | / | / | / | 1.34 | / |
| 84 | *VIT_08s0007g02030* | K13946 | AUX1 | auxin influx carrier (AUX1 LAX family) | Auxin transporter-like protein 2 | / | / | -1.35 | / | / | -1.04 |
| 85 | *VIT_13s0067g00330* | K13946 | AUX1 | auxin influx carrier (AUX1 LAX family) | Auxin transporter-like protein 2 | / | / | / | / | / | -1.30 |
| 86 | *VIT_04s0008g00210* | K14490 | AHP | histidine-containing phosphotransfer peotein | Pseudo histidine-containing phosphotransfer protein 5 OS | / | -2.81 | -3.56 | / | -1.30 | -2.47 |
| 87 | *VIT_09s0002g03520* | K14490 | AHP | histidine-containing phosphotransfer peotein | Histidine-containing phosphotransfer protein 4 | / | -5.45 | -2.70 | / | / | -4.16 |
| 88 | *VIT_05s0020g02210* | K14490 | AHP | histidine-containing phosphotransfer peotein | Histidine-containing phosphotransfer protein 1 | / | -1.04 | -1.26 | / | / | / |
| 89 | *VIT_14s0030g00410* | K14490 | AHP | histidine-containing phosphotransfer peotein | Histidine-containing phosphotransfer protein 1 | / | -1.06 | / | / | / | / |
| 90 | *VIT_02s0012g01270* | K14496 | PYL | abscisic acid receptor PYR/PYL family | Abscisic acid receptor PYL1 | / | / | 1.42 | / | / | 1.58 |
| 91 | *VIT_08s0058g00470* | K14496 | PYL | abscisic acid receptor PYR/PYL family | Abscisic acid receptor PYL4 | / | / | 1.12 | / | / | / |
| 92 | *VIT_15s0046g01050* | K14496 | PYL | abscisic acid receptor PYR/PYL family | Abscisic acid receptor PYL9 | / | -1.08 | / | / | -1.09 | / |
| 93 | *VIT_04s0008g00890* | K14496 | PYL | abscisic acid receptor PYR/PYL family | Abscisic acid receptor PYL2 | -2.25 | / | -1.79 | -1.91 | / | / |
| 94 | *VIT_18s0001g01240* | K14505 | CYCD3 | cyclin D3, plant | unnamed protein product, partial | / | -4.51 | -2.64 | / | -6.58 | -2.06 |
| 95 | *VIT_07s0129g01100* | K14505 | CYCD3 | cyclin D3, plant | Cyclin-D3-2 | / | / | -1.50 | / | / | -2.14 |
| 96 | *VIT_03s0180g00040* | K14505 | CYCD3 | cyclin D3, plant | Cyclin-D3-2 | / | / | / | / | -2.31 | / |
| 97 | *VIT_18s0001g06310* | K14498 | SNRK2 | serine/threonine-protein kinase SRK2 [EC:2.7.11.1] | Serine/threonine-protein kinase SAPK2 | / | 1.03 | 1.33 | / | 1.01 | 1.30 |
| 98 | *VIT_00s0710g00020* | K14498 | SNRK2 | serine/threonine-protein kinase SRK2 [EC:2.7.11.1] | Serine/threonine-protein kinase SAPK2 | / | / | -1.25 | -1.56 | / | / |
| 99 | *VIT_07s0191g00070* | K14498 | SNRK2 | serine/threonine-protein kinase SRK2 [EC:2.7.11.1] | Serine/threonine-protein kinase SAPK2 | / | -1.03 | / | / | / | / |
| 100 | *VIT_07s0031g03210* | K14498 | SNRK2 | serine/threonine-protein kinase SRK2 [EC:2.7.11.1] | Serine/threonine-protein kinase SAPK7 | / | / | -1.51 | / | / | -1.27 |
| 101 | *VIT_06s0004g05460* | K14497 | PP2C | protein phosphatase 2C [EC:3.1.3.16] | Probable protein phosphatase 2C 78 | 2.24 | / | / | / | / | / |
| 102 | *VIT_11s0016g03170* | K14497 | PP2C | protein phosphatase 2C [EC:3.1.3.16] | Pseudo histidine-containing phosphotransfer protein 2 | / | / | -1.04 | / | -1.01 | -1.20 |
| 103 | *VIT_11s0016g03180* | K14497 | PP2C | protein phosphatase 2C [EC:3.1.3.16] | Probable protein phosphatase 2C 6 | / | / | / | / | -2.11 | / |
| 104 | *VIT_08s0007g06160* | K14431 | TGA | transcription factor TGA | bZIP transcription factor TGA10 | 3.77 | 3.31 | 5.10 | 4.67 | 3.41 | 4.69 |

| | | | | | | | | | | | |
|---|---|---|---|---|---|---|---|---|---|---|---|
| 105 | *VIT_07s0031g01320* | K14431 | TGA | transcription factor TGA | Transcription factor TGA1 | / | / | / | -1.10 | / | / |
| 106 | *VIT_07s0104g01320* | K14485 | TIR1 | transport inhibitor response 1 | protein TRANSPORT INHIBITOR RESPONSE 1 | / | / | / | / | -1.21 | / |
| 107 | *VIT_00s0181g00040* | K14485 | TIR1 | transport inhibitor response 1 | Protein TRANSPORT INHIBITOR RESPONSE 1 | / | / | / | -1.25 | / | / |
| 108 | *VIT_01s0011g04220* | K14491 | ARR-B, APRR6 | two-component response regulator ARR-B family | Myb family transcription factor PHL13 | / | -1.05 | 1.77 | / | / | / |
| 109 | *VIT_17s0000g10110* | K14491 | ARR-B, APRR6 | two-component response regulator ARR-B family | Two-component response regulator ORR26 | / | / | -5.38 | / | 2.27 | / |
| 110 | *VIT_11s0016g05410* | K14515 | EBF1_2 | EIN3-binding F-box protein | EIN3-binding F-box protein 1 | / | / | 1.20 | / | / | 1.46 |
| 111 | *VIT_07s0104g00930* | K14493 | GID1, ATGID1B | gibberellin receptor GID1 | Gibberellin receptor GID1B | / | / | 1.03 | / | / | 1.25 |
| 112 | *VIT_07s0129g01000* | K14495 | GID2, SLY1 | F-box protein GID2 | F-box protein GID2 | / | / | / | / | -1.11 | / |
| 113 | *VIT_01s0011g05260* | K14494 | DELLA | DELLA protein | DELLA protein GAI1 | / | / | / | / | -1.21 | / |
| 114 | *VIT_09s0018g01820* | K13413 | MKK4_5 | mitogen-activated protein kinase kinase 4/5 [EC:2.7.12.2] | Mitogen-activated protein kinase kinase 5 | / | 1.05 | / | / | / | / |
| 115 | *VIT_04s0023g01250* | K14503 | BZR1_2; BEH2 | brassinosteroid resistant 1/2 | BES1/BZR1 homolog protein 2 | / | / | / | / | 1.00 | / |
| 116 | *VIT_18s0001g10690* | K14499 | BKI1 | BRI1 kinase inhibitor 1 | BRI1 kinase inhibitor 1 | / | -1.29 | -1.01 | -1.15 | -2.26 | / |
| 117 | *VIT_07s0005g02510* | K12126 | PIF3 | phytochrome-interacting factor 3 | Transcription factor APG | / | -1.16 | -2.97 | / | -1.09 | -2.28 |
| 118 | *VIT_07s0005g05100* | K16189 | PIF4 | phytochrome-interacting factor 4 | Transcription factor PIF1 | / | / | -1.25 | / | / | -1.00 |
| 119 | *VIT_07s0031g01850* | K13415 | BRI1 | protein brassinosteroid insensitive 1 [EC:2.7.10.1 2.7.11.1] | Systemin receptor SR160 | / | / | / | / | -1.11 | / |
| 120 | *VIT_01s0011g06190* | K14489 | CRE1 | arabidopsis histidine kinase 2/3/4 (cytokinin receptor) [EC:2.7.13.3] | Histidine kinase 4 | / | / | -2.35 | / | / | / |
| 121 | *VIT_08s0007g05740* | K14508 | NPR1 | regulatory protein NPR1 | Regulatory protein NPR5 | / | / | / | / | -1.52 | / |
| 122 | *VIT_15s0048g02820* | K13422 | MYC2 | transcription factor MYC2 | Transcription factor bHLH14 | / | -1.75 | / | / | -2.32 | 1.76 |

**Table S5.** DEGs related to the phenylalanine metabolism pathway of the tissue cultured seedlings of 'Rose Honey' in response to endophytes fungi Epi R2-21 and Alt XHYN2 after 6 h, 6 d, and 15 d inoculation, respectively (|$Log_2FC$| ≥ 1; *p*-adjust < 0.05).

| No. | gene ID | KO ID | Gene product | KEGG definition | Gene description | Log2FC | | | | | |
|---|---|---|---|---|---|---|---|---|---|---|---|
| | | | | | | R2-21_6h vs Con_6h | R2-21_6d vs Con_6d | R2-21_15d vs Con_15d | XHYN2_6h vs Con_6h | XHYN2_6d vs Con_6d | XHYN2_15d vs Con_15d |
| 1 | *VIT_16s0039g01240* | K10775 | PAL | phenylalanine ammonia-lyase [EC:4.3.1.24] | Phenylalanine ammonia-lyase | 3.19 | 5.96 | 7.56 | 4.20 | 6.99 | 7.33 |
| 2 | *VIT_16s0039g01300* | K10775 | PAL | phenylalanine ammonia-lyase [EC:4.3.1.24] | Phenylalanine ammonia-lyase | 2.79 | 6.21 | 7.25 | 4.15 | 7.40 | 7.44 |
| 3 | *VIT_00s2849g00010* | K10775 | PAL | phenylalanine ammonia-lyase [EC:4.3.1.24] | Phenylalanine ammonia-lyase | 2.94 | 6.17 | 7.59 | 4.49 | 7.48 | 7.58 |
| 4 | *VIT_00s2508g00010* | K10775 | PAL | phenylalanine ammonia-lyase [EC:4.3.1.24] | Phenylalanine ammonia-lyase | 2.74 | 6.21 | 8.38 | 4.44 | 7.29 | 8.22 |
| 5 | *VIT_16s0039g01130* | K10775 | PAL | phenylalanine ammonia-lyase [EC:4.3.1.24] | Phenylalanine ammonia-lyase | 3.01 | 5.06 | 6.23 | 4.56 | 6.90 | 6.39 |
| 6 | *VIT_16s0039g01110* | K10775 | PAL | phenylalanine ammonia-lyase [EC:4.3.1.24] | Phenylalanine ammonia-lyase | 2.98 | 5.71 | 6.39 | 4.07 | 6.78 | 6.16 |
| 7 | *VIT_16s0039g01360* | K10775 | PAL | phenylalanine ammonia-lyase [EC:4.3.1.24] | Phenylalanine ammonia-lyase | 3.60 | 6.88 | 8.15 | 5.17 | 8.55 | 8.28 |
| 8 | *VIT_16s0039g01280* | K10775 | PAL | phenylalanine ammonia-lyase [EC:4.3.1.24] | Phenylalanine ammonia-lyase | 3.34 | 5.62 | 6.44 | 4.59 | 6.67 | 6.16 |
| 9 | *VIT_16s0039g01170* | K10775 | PAL | phenylalanine ammonia-lyase [EC:4.3.1.24] | Phenylalanine ammonia-lyase | 2.71 | 4.83 | 5.39 | 3.87 | 5.72 | 4.94 |
| 10 | *VIT_08s0040g01710* | K10775 | PAL | phenylalanine ammonia-lyase [EC:4.3.1.24] | Phenylalanine ammonia-lyase | 3.16 | 3.12 | 3.40 | 4.37 | 3.72 | 3.18 |
| 11 | *VIT_16s0039g01120* | K10775 | PAL | phenylalanine ammonia-lyase [EC:4.3.1.24] | Phenylalanine ammonia-lyase | 3.17 | 5.38 | 5.46 | 4.47 | 6.40 | 4.89 |
| 12 | *VIT_16s0039g01100* | K10775 | PAL | phenylalanine ammonia-lyase [EC:4.3.1.24] | Phenylalanine ammonia-lyase | / | 4.46 | 4.48 | 3.81 | 5.44 | 3.84 |
| 13 | *VIT_16s0039g01320* | K10775 | PAL | phenylalanine ammonia-lyase [EC:4.3.1.24] | Phenylalanine ammonia-lyase | / | 5.53 | 9.13 | / | 6.68 | 9.04 |
| 14 | *VIT_13s0019g04460* | K10775 | PAL | phenylalanine ammonia-lyase [EC:4.3.1.24] | Phenylalanine ammonia-lyase | / | 2.32 | 2.49 | / | 2.49 | 1.97 |
| 15 | *VIT_11s0016g01520* | K10775 | PAL | phenylalanine ammonia-lyase [EC:4.3.1.24] | Phenylalanine ammonia-lyase | / | / | / | / | 2.84 | / |
| 16 | *VIT_11s0016g01640* | K10775 | PAL | phenylalanine ammonia-lyase [EC:4.3.1.24] | Phenylalanine ammonia-lyase | / | / | / | / | 2.39 | / |
| 17 | *VIT_06s0004g02620* | K10775 | PAL | phenylalanine ammonia-lyase [EC:4.3.1.24] | Phenylalanine ammonia-lyase | -3.86 | -3.11 | 1.56 | -2.37 | -3.35 | / |
| 18 | *VIT_11s0016g01660* | K10775 | PAL | phenylalanine ammonia-lyase [EC:4.3.1.24] | Phenylalanine ammonia-lyase | / | / | / | / | -1.08 | / |
| 19 | *VIT_11s0052g01090* | K01904 | 4CL | 4-coumarate--CoA ligase [EC:6.2.1.12] | 4-coumarate--CoA ligase 1 | / | 3.00 | 2.49 | / | 3.92 | 2.74 |

| | | | | | | | | | | | |
|---|---|---|---|---|---|---|---|---|---|---|---|
| 20 | *VIT_11s0052g01110* | K01904 | 4CL | 4-coumarate--CoA ligase [EC:6.2.1.12] | 4-coumarate--CoA ligase 1 | / | / | 4.76 | / | 5.67 | 2.89 |
| 21 | *VIT_06s0061g00450* | K01904 | 4CL | 4-coumarate--CoA ligase [EC:6.2.1.12] | 4-coumarate--CoA ligase-like 7 | / | / | 1.88 | / | / | 1.85 |
| 22 | *VIT_16s0039g02040* | K01904 | 4CL | 4-coumarate--CoA ligase [EC:6.2.1.12] | 4-coumarate--CoA ligase 2 | / | 1.02 | / | / | 1.42 | / |
| 23 | *VIT_11s0065g00350* | K00487 | CYP73A | trans-cinnamate 4-monooxygenase [EC:1.14.14.91] | Cytochrome P450 CYP73A100 | 2.79 | 4.36 | 5.66 | 4.04 | 5.22 | 5.61 |
| 24 | *VIT_11s0078g00290* | K00487 | CYP73A | trans-cinnamate 4-monooxygenase [EC:1.14.14.91] | Cytochrome P450 CYP73A100 | / | 4.38 | 7.05 | / | 4.52 | 7.07 |
| 25 | *VIT_06s0004g08150* | K00487 | CYP73A | trans-cinnamate 4-monooxygenase [EC:1.14.14.91] | Trans-cinnamate 4-monooxygenase | / | / | 1.10 | 2.27 | / | 1.27 |
| 26 | *VIT_19s0014g02190* | K00815 | TAT | tyrosine aminotransferase [EC:2.6.1.5] | Probable aminotransferase TAT2 | / | 1.86 | 2.72 | / | 1.92 | 2.73 |
| 27 | *VIT_12s0028g03260* | K00815 | TAT | tyrosine aminotransferase [EC:2.6.1.5] | S-alkyl-thiohydroximate lyase SUR1 | / | / | 2.65 | / | 1.03 | 2.02 |
| 28 | *VIT_12s0028g03240* | K00815 | TAT | tyrosine aminotransferase [EC:2.6.1.5] | Tyrosine aminotransferase | / | / | 1.26 | / | / | / |
| 29 | *VIT_05s0020g03280* | K00276 | AOC3 | primary-amine oxidase [EC:1.4.3.21] | Primary amine oxidase | / | 3.26 | 5.50 | / | 2.88 | 4.87 |
| 30 | *VIT_00s0225g00090* | K00276 | AOC3 | primary-amine oxidase [EC:1.4.3.21] | Primary amine oxidase | / | 2.62 | 2.08 | / | 3.19 | 2.17 |
| 31 | *VIT_17s0000g09100* | K00276 | AOC3 | primary-amine oxidase [EC:1.4.3.21] | Primary amine oxidase | / | 1.34 | 1.39 | / | 1.10 | 1.27 |
| 32 | *VIT_05s0020g03260* | K00276 | AOC3 | primary-amine oxidase [EC:1.4.3.21] | Primary amine oxidase | / | / | / | / | 3.59 | / |
| 33 | *VIT_02s0025g04560* | K00276 | AOC3 | primary-amine oxidase [EC:1.4.3.21] | Primary amine oxidase | / | / | 1.04 | / | -1.10 | / |
| 34 | *VIT_05s0020g03310* | K00276 | AOC3 | primary-amine oxidase [EC:1.4.3.21] | Primary amine oxidase 1 | / | / | -3.16 | / | / | / |
| 35 | *VIT_04s0008g03770* | K14454 | GOT1 | aspartate aminotransferase, cytoplasmic [EC:2.6.1.1] | Aspartate aminotransferase 3, chloroplastic | / | 1.92 | 2.84 | / | 2.15 | 2.55 |
| 36 | *VIT_12s0028g01820* | K14455 | GOT2 | aspartate aminotransferase, mitochondrial [EC:2.6.1.1] | Aspartate aminotransferase, mitochondrial | / | 1.17 | 1.65 | / | / | 1.47 |
| 37 | *VIT_08s0058g01000* | K14455 | GOT2 | aspartate aminotransferase, mitochondrial [EC:2.6.1.1] | Aspartate aminotransferase, mitochondrial | / | / | 2.60 | / | 1.40 | / |
| 38 | *VIT_18s0001g04860* | K15849 | PAT, AAT | bifunctional aspartate aminotransferase and glutamate/aspartate-prephenate aminotransferase [EC:2.6.1.1 2.6.1.78 | Bifunctional aspartate aminotransferase and glutamate/aspartate-prephenate aminotransferase | / | 1.22 | 2.42 | / | 1.77 | 2.13 |

| | | | | | | | | | | | |
|---|---|---|---|---|---|---|---|---|---|---|---|
| | | | | 2.6.1.79] | | | | | | | |
| 39 | *VIT_07s0031g00960* | K15849 | PAT, AAT | bifunctional aspartate aminotransferase and glutamate/aspartate-prephenate aminotransferase [EC:2.6.1.1 2.6.1.78 2.6.1.79] | Bifunctional aspartate aminotransferase and glutamate/aspartate-prephenate aminotransferase | 1.73 | / | / | 2.44 | / | 1.90 |
| 40 | *VIT_07s0031g00980* | K15849 | PAT, AAT | bifunctional aspartate aminotransferase and glutamate/aspartate-prephenate aminotransferase [EC:2.6.1.1 2.6.1.78 2.6.1.79] | Bifunctional aspartate aminotransferase and glutamate/aspartate-prephenate aminotransferase | / | / | / | 2.53 | / | / |
| 41 | *VIT_07s0031g00350* | K00588 | CCoAOMT | caffeoyl-CoA O-methyltransferase [EC:2.1.1.104] | Caffeoyl-CoA O-methyltransferase | / | 2.58 | 3.00 | / | 2.65 | 3.22 |
| 42 | *VIT_12s0028g03110* | K00588 | CCoAOMT | caffeoyl-CoA O-methyltransferase [EC:2.1.1.104] | Caffeoyl-CoA O-methyltransferase | / | / | / | / | / | -1.25 |
| 43 | *VIT_12s0028g00710* | K00457 | HPD, hppD | 4-hydroxyphenylpyruvate dioxygenase [EC:1.13.11.27] | 4-hydroxyphenylpyruvate dioxygenase | / | 1.33 | 2.82 | / | 2.08 | 2.74 |
| 44 | *VIT_03s0063g00140* | K00588 | ATAMI1 | caffeoyl-CoA O-methyltransferase [EC:2.1.1.104] | Caffeoyl-CoA O-methyltransferase | / | 2.61 | 1.95 | / | 2.20 | 1.92 |
| 45 | *VIT_11s0052g00040* | K07253 | MIF | phenylpyruvate tautomerase [EC:5.3.2.1] | Macrophage migration inhibitory factor homolog | / | / | -1.26 | / | / | -1.09 |
| 46 | *VIT_03s0038g02010* | K01426 | amiE | amidase [EC:3.5.1.4] | Probable amidase At4g34880 | / | / | / | / | -3.78 | / |

**Table S6.** DEGs related to the phenylpropanoid biosynthesis pathway of the tissue cultured seedlings of ‘Rose Honey’ in response to endophytes fungi Epi R2-21 and Alt XHYN2 after 6 h, 6 d, and 15 d inoculation, respectively ($|Log_2FC| \geq 1$; *p*-adjust < 0.05).

| No. | gene ID | KO ID | Gene product | KEGG definition | Gene description | Log₂FC | | | | | |
|---|---|---|---|---|---|---|---|---|---|---|---|
| | | | | | | **R2-21_ 6h vs Con_6h** | **R2-21_ 6d vs Con_6d** | **R2-21_15 d vs Con_15d** | **XHYN2 _6h vs Con_6h** | **XHYN2 _6d vs Con_6d** | **XHYN2_ 15d vs Con_15d** |
| 1 | *VIT_16s0039g01240* | K10775 | PAL | phenylalanine ammonia-lyase [EC:4.3.1.24] | Phenylalanine ammonia-lyase | 3.19 | 5.96 | 7.56 | 4.20 | 6.99 | 7.33 |
| 2 | *VIT_00s2849g00010* | K10775 | PAL | phenylalanine ammonia-lyase [EC:4.3.1.24] | Phenylalanine ammonia-lyase | 2.94 | 6.17 | 7.59 | 4.49 | 7.48 | 7.58 |
| 3 | *VIT_00s2508g00010* | K10775 | PAL | phenylalanine ammonia-lyase [EC:4.3.1.24] | Phenylalanine ammonia-lyase | 2.74 | 6.21 | 8.38 | 4.44 | 7.29 | 8.22 |
| 4 | *VIT_16s0039g01110* | K10775 | PAL | phenylalanine ammonia-lyase [EC:4.3.1.24] | Phenylalanine ammonia-lyase | 2.98 | 5.71 | 6.39 | 4.07 | 6.78 | 6.16 |
| 5 | *VIT_16s0039g01300* | K10775 | PAL | phenylalanine ammonia-lyase [EC:4.3.1.24] | Phenylalanine ammonia-lyase | 2.79 | 6.21 | 7.44 | 4.15 | 7.40 | 7.25 |
| 6 | *VIT_16s0039g01360* | K10775 | PAL | phenylalanine ammonia-lyase [EC:4.3.1.24] | Phenylalanine ammonia-lyase | 3.60 | 6.88 | 8.15 | 5.17 | 8.55 | 8.28 |
| 7 | *VIT_16s0039g01130* | K10775 | PAL | phenylalanine ammonia-lyase [EC:4.3.1.24] | Phenylalanine ammonia-lyase | 3.01 | 5.06 | 6.23 | 4.56 | 6.90 | 6.39 |
| 8 | *VIT_16s0039g01280* | K10775 | PAL | phenylalanine ammonia-lyase [EC:4.3.1.24] | Phenylalanine ammonia-lyase | 3.34 | 5.62 | 6.44 | 4.59 | 6.67 | 6.16 |
| 9 | *VIT_16s0039g01120* | K10775 | PAL | phenylalanine ammonia-lyase [EC:4.3.1.24] | Phenylalanine ammonia-lyase | 3.17 | 5.35 | 5.41 | 4.46 | 6.36 | 4.86 |
| 10 | *VIT_16s0039g01170* | K10775 | PAL | phenylalanine ammonia-lyase [EC:4.3.1.24] | Phenylalanine ammonia-lyase | 2.71 | 4.83 | 5.39 | 3.87 | 5.72 | 4.94 |
| 11 | *VIT_08s0040g01710* | K10775 | PAL | phenylalanine ammonia-lyase [EC:4.3.1.24] | Phenylalanine ammonia-lyase | 3.16 | 3.12 | 3.40 | 4.37 | 3.72 | 3.18 |
| 12 | *VIT_16s0039g01100* | K10775 | PAL | phenylalanine ammonia-lyase [EC:4.3.1.24] | Phenylalanine ammonia-lyase | / | 4.46 | 4.48 | 3.81 | 5.44 | 3.84 |
| 13 | *VIT_16s0039g01320* | K10775 | PAL | phenylalanine ammonia-lyase [EC:4.3.1.24] | Phenylalanine ammonia-lyase | / | 5.53 | 9.13 | / | 6.68 | 9.04 |

| | | | | | | | | | | | |
|---|---|---|---|---|---|---|---|---|---|---|---|
| 14 | *VIT_13s0019g04460* | K10775 | PAL | phenylalanine ammonia-lyase [EC:4.3.1.24] | Phenylalanine ammonia-lyase | / | 2.32 | 2.49 | / | 2.49 | 1.97 |
| 15 | *VIT_11s0016g01520* | K10775 | PAL | phenylalanine ammonia-lyase [EC:4.3.1.24] | Phenylalanine ammonia-lyase | / | / | / | / | 2.84 | / |
| 16 | *VIT_11s0016g01640* | K10775 | PAL | phenylalanine ammonia-lyase [EC:4.3.1.24] | Phenylalanine ammonia-lyase | / | / | / | / | 2.39 | / |
| 17 | *VIT_11s0016g01660* | K10775 | PAL | phenylalanine ammonia-lyase [EC:4.3.1.24] | Phenylalanine ammonia-lyase | / | / | / | / | -1.08 | / |
| 18 | *VIT_06s0004g02620* | K10775 | PAL | phenylalanine ammonia-lyase [EC:4.3.1.24] | Phenylalanine ammonia-lyase | -3.86 | -3.11 | 1.56 | -2.37 | -3.35 | / |
| 19 | *VIT_12s0055g00990* | K00430 | POD | peroxidase [EC:1.11.1.7] | Peroxidase N1 | 1.81 | 2.20 | / | 2.18 | 2.60 | / |
| 20 | *VIT_01s0010g02010* | K00430 | POD | peroxidase [EC:1.11.1.7] | Lignin-forming anionic peroxidase | / | 8.27 | 9.16 | 5.68 | 7.31 | 7.77 |
| 21 | *VIT_06s0004g07770* | K00430 | POD | peroxidase [EC:1.11.1.7] | Peroxidase 4 | / | 2.82 | 4.19 | 2.78 | 3.66 | 4.36 |
| 22 | *VIT_08s0058g00970* | K00430 | POD | peroxidase [EC:1.11.1.7] | Cationic peroxidase 1 | / | 1.98 | 2.58 | / | 2.33 | 2.47 |
| 23 | *VIT_08s0058g00980* | K00430 | POD | peroxidase [EC:1.11.1.7] | Cationic peroxidase 1 | / | 2.67 | 2.65 | / | 3.21 | 2.91 |
| 24 | *VIT_07s0129g00360* | K00430 | POD | peroxidase [EC:1.11.1.7] | Peroxidase 73 | / | 1.43 | 3.23 | / | 1.00 | 3.45 |
| 25 | *VIT_12s0055g01010* | K00430 | POD | peroxidase [EC:1.11.1.7] | Peroxidase N1 | / | 3.24 | 2.36 | / | 2.92 | 2.64 |
| 26 | *VIT_18s0001g06850* | K00430 | POD | peroxidase [EC:1.11.1.7] | Cationic peroxidase 1 | / | 3.14 | 2.32 | / | 2.93 | 2.58 |
| 27 | *VIT_14s0068g01920* | K00430 | POD | peroxidase [EC:1.11.1.7] | Peroxidase 55 | / | 1.68 | 4.31 | / | 1.08 | 3.64 |
| 28 | *VIT_08s0058g00990* | K00430 | POD | peroxidase [EC:1.11.1.7] | Cationic peroxidase 1 | / | 6.47 | 6.95 | / | 6.53 | 5.37 |
| 29 | *VIT_01s0010g02020* | K00430 | POD | peroxidase [EC:1.11.1.7] | Lignin-forming anionic peroxidase | / | / | 7.48 | / | 8.84 | 5.63 |
| 30 | *VIT_05s0020g02120* | K00430 | POD | peroxidase [EC:1.11.1.7] | Peroxidase 24 | / | / | 10.15 | / | 9.68 | 9.95 |
| 31 | *VIT_18s0072g00160* | K00430 | POD | peroxidase [EC:1.11.1.7] | Peroxidase 12 | / | 1.06 | 2.43 | / | / | 2.27 |
| 32 | *VIT_01s0010g01950* | K00430 | POD | peroxidase [EC:1.11.1.7] | Lignin-forming anionic peroxidase | / | / | 7.67 | / | 7.17 | 6.08 |
| 33 | *VIT_14s0060g00540* | K00430 | POD | peroxidase [EC:1.11.1.7] | Peroxidase 5 | / | 5.37 | 4.67 | / | 5.22 | / |
| 34 | *VIT_14s0060g00520* | K00430 | POD | peroxidase [EC:1.11.1.7] | Peroxidase 5 | / | 4.14 | 3.35 | / | 3.83 | / |
| 35 | *VIT_04s0023g02570* | K00430 | POD | peroxidase [EC:1.11.1.7] | Peroxidase 72 | / | / | / | 1.75 | 2.64 | / |
| 36 | *VIT_04s0008g07040* | K00430 | POD | peroxidase [EC:1.11.1.7] | Peroxidase 18 | / | / | / | / | 2.76 | / |
| 37 | *VIT_12s0055g01020* | K00430 | POD | peroxidase [EC:1.11.1.7] | Peroxidase N1 | / | / | 2.04 | / | 2.39 | / |
| 38 | *VIT_06s0004g07740* | K00430 | POD | peroxidase [EC:1.11.1.7] | Cationic peroxidase 1 | / | 5.95 | / | / | 5.93 | / |
| 39 | *VIT_01s0010g01970* | K00430 | POD | peroxidase [EC:1.11.1.7] | Lignin-forming anionic peroxidase | / | 5.85 | / | / | 6.67 | / |
| 40 | *VIT_01s0010g01980* | K00430 | POD | peroxidase [EC:1.11.1.7] | Lignin-forming anionic peroxidase | / | 7.80 | / | / | 8.63 | / |
| 41 | *VIT_14s0060g00510* | K00430 | POD | peroxidase [EC:1.11.1.7] | Peroxidase 5 | / | 6.33 | / | / | 5.20 | / |
| 42 | *VIT_01s0010g02000* | K00430 | POD | peroxidase [EC:1.11.1.7] | Lignin-forming anionic peroxidase | / | 6.50 | / | / | 5.28 | / |
| 43 | *VIT_12s0028g01840* | K00430 | POD | peroxidase [EC:1.11.1.7] | Peroxidase 52 | / | / | / | / | 1.25 | 2.83 |
| 44 | *VIT_01s0010g01960* | K00430 | POD | peroxidase [EC:1.11.1.7] | Lignin-forming anionic | / | / | 7.20 | / | 7.20 | / |

| | | | | | | | | | | | |
|---|---|---|---|---|---|---|---|---|---|---|---|
| | | | | | peroxidase | | | | | | |
| 45 | *VIT_06s0004g01190* | K00430 | POD | peroxidase [EC:1.11.1.7] | Lignin-forming anionic peroxidase | 3.72 | / | / | 4.67 | / | / |
| 46 | *VIT_12s0055g01000* | K00430 | POD | peroxidase [EC:1.11.1.7] | Peroxidase N1 | 3.31 | / | / | 4.10 | / | / |
| 47 | *VIT_12s0055g01070* | K00430 | POD | peroxidase [EC:1.11.1.7] | Cationic peroxidase 2 | / | / | / | / | 8.72 | / |
| 48 | *VIT_01s0026g00830* | K00430 | POD | peroxidase [EC:1.11.1.7] | Peroxidase 65 | / | / | / | / | 7.43 | / |
| 49 | *VIT_12s0055g01030* | K00430 | POD | peroxidase [EC:1.11.1.7] | Peroxidase N1 | / | / | 1.61 | / | / | / |
| 50 | *VIT_18s0001g06890* | K00430 | POD | peroxidase [EC:1.11.1.7] | Cationic peroxidase 1 | / | 1.70 | -2.75 | / | 2.60 | / |
| 51 | *VIT_18s0001g06840* | K00430 | POD | peroxidase [EC:1.11.1.7] | Cationic peroxidase 1 | / | / | -3.18 | / | 1.88 | / |
| 52 | *VIT_14s0066g01850* | K00430 | POD | peroxidase [EC:1.11.1.7] | Peroxidase 31 | / | / | / | / | 1.06 | -1.12 |
| 53 | *VIT_11s0016g05320* | K00430 | POD | peroxidase [EC:1.11.1.7] | Peroxidase 25 | / | / | / | / | -1.92 | / |
| 54 | *VIT_07s0191g00050* | K00430 | POD | peroxidase [EC:1.11.1.7] | Peroxidase 17 | -1.93 | -1.57 | -1.85 | -1.85 | -1.19 | -1.49 |
| 55 | *VIT_11s0016g05280* | K00430 | POD | peroxidase [EC:1.11.1.7] | Peroxidase 25 | / | -1.31 | / | / | -1.84 | / |
| 56 | *VIT_06s0004g01180* | K00430 | POD | peroxidase [EC:1.11.1.7] | Peroxidase 15 | / | / | / | / | -1.21 | / |
| 57 | *VIT_12s0055g00810* | K00430 | POD | peroxidase [EC:1.11.1.7] | Peroxidase 43 | / | / | -1.79 | / | / | -1.25 |
| 58 | *VIT_12s0059g02420* | K00430 | POD | peroxidase [EC:1.11.1.7] | Peroxidase 3 | / | / | -5.31 | / | 3.14 | -4.64 |
| 59 | *VIT_05s0077g00880* | K00430 | POD | peroxidase [EC:1.11.1.7] | Peroxidase 29 | / | / | / | -1.90 | -2.06 | -1.38 |
| 60 | *VIT_10s0116g00340* | K00430 | POD | peroxidase [EC:1.11.1.7] | Peroxidase 27 | / | / | / | / | -3.83 | / |
| 61 | *VIT_00s1677g00010* | K00430 | POD | peroxidase [EC:1.11.1.7] | Peroxidase 64 | / | / | -1.81 | / | / | -1.96 |
| 62 | *VIT_10s0116g01780* | K00430 | POD | peroxidase [EC:1.11.1.7] | Peroxidase 42 | / | / | -3.82 | / | / | -2.71 |
| 63 | *VIT_07s0130g00220* | K00430 | POD | peroxidase [EC:1.11.1.7] | Peroxidase 47 | -1.70 | / | / | -1.63 | / | / |
| 64 | *VIT_10s0003g04910* | K00083 | CAD | cinnamyl-alcohol dehydrogenase [EC:1.1.1.195] | Probable mannitol dehydrogenase | / | 8.79 | 7.56 | 7.04 | 8.95 | 6.20 |
| 65 | *VIT_00s0174g00280* | K00083 | CAD | cinnamyl-alcohol dehydrogenase [EC:1.1.1.195] | Probable cinnamyl alcohol dehydrogenase 1 | / | 3.47 | 4.37 | / | 3.70 | 4.69 |
| 66 | *VIT_00s0174g00270* | K00083 | CAD | cinnamyl-alcohol dehydrogenase [EC:1.1.1.195] | Probable cinnamyl alcohol dehydrogenase 1 | 3.87 | 2.78 | 4.46 | / | 2.74 | / |
| 67 | *VIT_07s0129g01030* | K00083 | CAD | cinnamyl-alcohol dehydrogenase [EC:1.1.1.195] | Probable cinnamyl alcohol dehydrogenase | 1.40 | / | 1.39 | 1.29 | / | / |
| 68 | *VIT_00s0346g00110* | K00083 | CAD | cinnamyl-alcohol dehydrogenase [EC:1.1.1.195] | Probable mannitol dehydrogenase | / | / | 4.28 | / | 4.86 | / |
| 69 | *VIT_00s0371g00050* | K00083 | CAD | cinnamyl-alcohol dehydrogenase [EC:1.1.1.195] | Probable mannitol dehydrogenase | / | / | 5.44 | / | 5.15 | / |
| 70 | *VIT_18s0122g00450* | K00083 | CAD | cinnamyl-alcohol dehydrogenase [EC:1.1.1.195] | Probable mannitol dehydrogenase | / | 3.95 | 5.47 | / | / | / |
| 71 | *VIT_18s0001g14910* | K00083 | CAD | cinnamyl-alcohol dehydrogenase [EC:1.1.1.195] | Probable cinnamyl alcohol dehydrogenase 6 | / | / | / | / | 1.56 | / |
| 72 | *VIT_00s0615g00010* | K00083 | CAD | cinnamyl-alcohol dehydrogenase | Probable mannitol dehydrogenase | / | / | / | / | 5.32 | / |

| | | | | | | | | | | | |
|---|---|---|---|---|---|---|---|---|---|---|---|
| | | | | [EC:1.1.1.195] | | | | | | | |
| 73 | *VIT_00s0218g00010* | K00083 | CAD | cinnamyl-alcohol dehydrogenase [EC:1.1.1.195] | Probable mannitol dehydrogenase | / | / | / | / | 2.52 | / |
| 74 | *VIT_00s0615g00020* | K00083 | CAD | cinnamyl-alcohol dehydrogenase [EC:1.1.1.195] | Probable mannitol dehydrogenase | / | / | -4.82 | / | 2.13 | / |
| 75 | *VIT_00s0615g00030* | K00083 | CAD | cinnamyl-alcohol dehydrogenase [EC:1.1.1.195] | Probable mannitol dehydrogenase | / | / | / | / | / | -1.12 |
| 76 | *VIT_00s0346g00090* | K00083 | CAD | cinnamyl-alcohol dehydrogenase [EC:1.1.1.195] | Probable mannitol dehydrogenase | / | -4.24 | -6.65 | / | -3.02 | -3.73 |
| 77 | *VIT_00s0346g00080* | K00083 | CAD | cinnamyl-alcohol dehydrogenase [EC:1.1.1.195] | Probable mannitol dehydrogenase | / | / | / | / | -1.26 | -1.90 |
| 78 | *VIT_04s0044g00190* | K00083 | CAD | cinnamyl-alcohol dehydrogenase [EC:1.1.1.195] | Probable mannitol dehydrogenase | / | / | / | / | / | -1.78 |
| 79 | *VIT_00s0371g00100* | K00083 | CAD | cinnamyl-alcohol dehydrogenase [EC:1.1.1.195] | Probable mannitol dehydrogenase | / | / | -1.15 | / | / | -1.47 |
| 80 | *VIT_18s0001g01160* | K00083 | CAD | cinnamyl-alcohol dehydrogenase [EC:1.1.1.195] | Probable cinnamyl alcohol dehydrogenase 6 | / | / | 1.18 | / | -1.11 | / |
| 81 | *VIT_06s0004g01430* | K01188 | | beta-glucosidase [EC:3.2.1.21] | Beta-glucosidase 12 | / | 2.23 | 2.91 | / | 3.08 | 2.42 |
| 82 | *VIT_07s0005g00360* | K01188 | bglA | beta-glucosidase [EC:3.2.1.21] | Beta-glucosidase 11 | / | / | 1.08 | / | 1.74 | 1.23 |
| 83 | *VIT_06s0004g01440* | K01188 | bglA | beta-glucosidase [EC:3.2.1.21] | Beta-glucosidase 10 | / | / | 4.42 | / | 7.12 | 3.68 |
| 84 | *VIT_13s0064g00480* | K01188 | bglA | beta-glucosidase [EC:3.2.1.21] | Furcatin hydrolase | / | / | / | / | 2.18 | / |
| 85 | *VIT_13s0064g01660* | K01188 | bglA | beta-glucosidase [EC:3.2.1.21] | Beta-glucosidase 13 | / | / | / | / | 3.02 | / |
| 86 | *VIT_13s0064g01750* | K01188 | bglA | beta-glucosidase [EC:3.2.1.21] | Beta-glucosidase 13 | / | / | / | / | 2.82 | / |
| 87 | *VIT_06s0004g01420* | K01188 | bglA | beta-glucosidase [EC:3.2.1.21] | Beta-glucosidase 12 | / | 1.18 | / | / | / | / |
| 88 | *VIT_07s0005g00390* | K01188 | bglA | beta-glucosidase [EC:3.2.1.21] | Beta-glucosidase 11 | -1.08 | -1.48 | -1.82 | / | -1.09 | -1.63 |
| 89 | *VIT_13s0064g01720* | K01188 | bglA | beta-glucosidase [EC:3.2.1.21] | Beta-glucosidase 12 | / | / | -6.00 | / | / | / |
| 90 | *VIT_00s0220g00070* | K01188 | bglA | beta-glucosidase [EC:3.2.1.21] | Beta-glucosidase 42 | / | / | -1.21 | / | / | -1.03 |
| 91 | *VIT_13s0064g01640* | K01188 | bglA | beta-glucosidase [EC:3.2.1.21] | Beta-glucosidase 13 | / | / | -5.09 | / | / | / |

| | | | | | | | | | | | |
|---|---|---|---|---|---|---|---|---|---|---|---|
| 92 | *VIT_13s0064g00520* | K01188 | bglA | beta-glucosidase [EC:3.2.1.21] | Furcatin hydrolase | / | / | -5.76 | / | 3.25 | -3.15 |
| 93 | *VIT_06s0009g00810* | K01188 | bglA | beta-glucosidase [EC:3.2.1.21] | Beta-glucosidase BoGH3B | / | / | -1.40 | / | / | -1.48 |
| 94 | *VIT_17s0000g01760* | K01188 | bglA | beta-glucosidase [EC:3.2.1.21] | Beta-glucosidase 24 | -2.60 | / | -2.83 | -2.82 | / | -3.04 |
| 95 | *VIT_17s0000g02680* | K05350 | bglB | beta-glucosidase [EC:3.2.1.21] | Beta-glucosidase 44 | / | -1.83 | -3.00 | / | -1.04 | -2.43 |
| 96 | *VIT_19s0014g03240* | K05350 | bglB | beta-glucosidase [EC:3.2.1.21] | Beta-glucosidase 18 | / | -1.25 | -2.00 | / | / | -1.26 |
| 97 | *VIT_14s0006g01790* | K05350 | bglB | beta-glucosidase [EC:3.2.1.21] | Beta-glucosidase 44 | -1.49 | / | / | -1.30 | / | / |
| 98 | *VIT_08s0032g00470* | K05349 | bglX | beta-glucosidase [EC:3.2.1.21] | Beta-glucosidase BoGH3B | / | 2.78 | 3.77 | / | 2.61 | 3.57 |
| 99 | *VIT_06s0009g00800* | K05349 | bglX | beta-glucosidase [EC:3.2.1.21] | Beta-glucosidase BoGH3B | / | / | / | / | 4.71 | 3.91 |
| 100 | *VIT_13s0073g00550* | K05349 | bglX | beta-glucosidase [EC:3.2.1.21] | Beta-glucosidase BoGH3B | / | / | / | / | -2.71 | / |
| 101 | *VIT_11s0065g00350* | K00487 | CYP73A | trans-cinnamate 4-monooxygenase [EC:1.14.14.91] | Cytochrome P450 CYP73A100 | 2.79 | 4.36 | 5.66 | 4.04 | 5.22 | 5.61 |
| 102 | *VIT_11s0078g00290* | K00487 | CYP73A | trans-cinnamate 4-monooxygenase [EC:1.14.14.91] | Cytochrome P450 CYP73A100 | / | 4.38 | 7.05 | / | 4.52 | 7.07 |
| 103 | *VIT_06s0004g08150* | K00487 | CYP73A | trans-cinnamate 4-monooxygenase [EC:1.14.14.91] | Trans-cinnamate 4-monooxygenase | / | / | 1.10 | 2.27 | / | 1.27 |
| 104 | *VIT_11s0052g01090* | K01904 | 4CL | 4-coumarate--CoA ligase [EC:6.2.1.12] | 4-coumarate--CoA ligase 1 | / | 3.00 | 2.49 | / | 3.92 | 2.74 |
| 105 | *VIT_16s0039g02040* | K01904 | 4CL | 4-coumarate--CoA ligase [EC:6.2.1.12] | 4-coumarate--CoA ligase 2 | / | 1.02 | / | / | 1.42 | / |
| 106 | *VIT_06s0061g00450* | K01904 | 4CL | 4-coumarate--CoA ligase [EC:6.2.1.12] | 4-coumarate--CoA ligase-like 7 | / | / | 1.88 | / | / | 1.85 |
| 107 | *VIT_11s0052g01110* | K01904 | 4CL | 4-coumarate--CoA ligase [EC:6.2.1.12] | 4-coumarate--CoA ligase 1 | / | / | 4.76 | / | 5.67 | / |
| 108 | *VIT_02s0025g02920* | K13066 | COMT | caffeic acid 3-O-methyltransferase / acetylserotonin O-methyltransferase [EC:2.1.1.68 2.1.1.4] | Caffeic acid 3-O-methyltransferase | / | 2.66 | 2.70 | / | 3.11 | 2.64 |
| 109 | *VIT_16s0098g00850* | K13066 | COMT | caffeic acid 3-O-methyltransferase / acetylserotonin O-methyltransferase [EC:2.1.1.68 2.1.1.4] | Caffeic acid 3-O-methyltransferase 1 | 1.94 | / | / | 2.40 | / | / |
| 110 | *VIT_15s0048g02490* | K13066 | COMT | caffeic acid 3-O-methyltransferase / | Flavone 3'-O-methyltransferase 1 | / | / | / | / | 4.33 | / |

| | | | | | | | | | | | |
|---|---|---|---|---|---|---|---|---|---|---|---|
| | | | | acetylserotonin O-methyltransferase [EC:2.1.1.68 2.1.1.4] | | | | | | | |
| 111 | *VIT_19s0135g00030* | K13066 | COMT | caffeic acid 3-O-methyltransferase / acetylserotonin O-methyltransferase [EC:2.1.1.68 2.1.1.4] | Caffeic acid 3-O-methyltransferase | / | / | / | / | / | 2.13 |
| 112 | *VIT_18s0001g02610* | K13066 | COMT | caffeic acid 3-O-methyltransferase / acetylserotonin O-methyltransferase [EC:2.1.1.68 2.1.1.4] | Caffeic acid 3-O-methyltransferase | / | / | / | / | / | 1.10 |
| 113 | *VIT_18s0072g00920* | K13066 | COMT | caffeic acid 3-O-methyltransferase / acetylserotonin O-methyltransferase [EC:2.1.1.68 2.1.1.4] | Caffeic acid 3-O-methyltransferase | -6.84 | 3.25 | / | / | 4.14 | / |
| 114 | *VIT_07s0031g00350* | K00588 | CCoAOMT | caffeoyl-CoA O-methyltransferase [EC:2.1.1.104] | Caffeoyl-CoA O-methyltransferase | / | 2.58 | 3.00 | / | 2.65 | 3.22 |
| 115 | *VIT_03s0063g00140* | K00588 | CCoAOMT | caffeoyl-CoA O-methyltransferase [EC:2.1.1.104] | Caffeoyl-CoA O-methyltransferase | / | 2.61 | 1.95 | / | 2.20 | 1.92 |
| 116 | *VIT_12s0028g03110* | K00588 | CCoAOMT | caffeoyl-CoA O-methyltransferase [EC:2.1.1.104] | Caffeoyl-CoA O-methyltransferase | / | / | / | / | / | -1.25 |
| 117 | *VIT_11s0037g00440* | K13065 | HCT | shikimate O-hydroxycinnamoyltransferase [EC:2.3.1.133] | Shikimate O-hydroxycinnamoyltransferase | / | 1.36 | 2.11 | / | / | 2.27 |
| 118 | *VIT_09s0018g01190* | K13065 | HCT | shikimate O-hydroxycinnamoyltransferase [EC:2.3.1.133] | Shikimate O-hydroxycinnamoyltransferase | / | / | / | 2.09 | / | / |
| 119 | *VIT_11s0037g00570* | K13065 | HCT | shikimate O-hydroxycinnamoyltransferase [EC:2.3.1.133] | Spermidine hydroxycinnamoyl transferase | / | / | -1.22 | / | -1.11 | / |
| 120 | *VIT_11s0037g00580* | K13065 | HCT | shikimate O-hydroxycinnamoyltransferase [EC:2.3.1.133] | Spermidine hydroxycinnamoyl transferase | / | / | -1.12 | / | / | / |
| 121 | *VIT_04s0023g01290* | K12356 | UGT72E | coniferyl-alcohol glucosyltransferase [EC:2.4.1.111] | Anthocyanidin 3-O-glucosyltransferase 5 | / | 2.89 | / | / | 3.68 | / |
| 122 | *VIT_04s0023g01240* | K12356 | UGT72E | coniferyl-alcohol glucosyltransferase [EC:2.4.1.111] | Anthocyanidin 3-O-glucosyltransferase 5 | / | -3.12 | -2.68 | / | -3.06 | -1.72 |
| 123 | *VIT_16s0022g01970* | K12356 | UGT72E | coniferyl-alcohol glucosyltransferase | Anthocyanidin 3-O-glucosyltransferase 5 | / | / | -7.45 | / | / | / |

| | | | | | | | | | | | |
|---|---|---|---|---|---|---|---|---|---|---|---|
| | | | | [EC:2.4.1.111] | | | | | | | |
| 124 | *VIT_04s0023g02900* | K09755 | CYP84A, F5H | ferulate-5-hydroxylase [EC:1.14.-.-] | Cytochrome P450 84A1 | / | 2.21 | 2.63 | 3.00 | 1.91 | 2.83 |
| 125 | *VIT_08s0040g00780* | K09754 | CYP98A, C3'H | 5-O-(4-coumaroyl)-D-quinate 3'-monooxygenase [EC:1.14.14.96] | Cytochrome P450 98A2 | 1.79 | / | / | 2.46 | / | / |
| 126 | *VIT_09s0070g00240* | K09753 | CCR | cinnamoyl-CoA reductase [EC:1.2.1.44] | Cinnamoyl-CoA reductase 1 | / | 1.33 | 1.83 | / | 1.18 | 2.22 |
| 127 | *VIT_05s0020g00600* | K11188 | PRDX6 | peroxidase [EC:1.11.1.7] | 1-Cys peroxiredoxin | / | / | 5.93 | / | / | / |
| 128 | *VIT_09s0002g05730* | K18368 | CSE | caffeoylshikimate esterase [EC:3.1.1.-] | Caffeoylshikimate esterase | / | / | / | 1.52 | / | / |

**Table S7.** DEGs related to the flavonoid biosynthesis pathway of the tissue cultured seedlings of 'Rose Honey' in response to endophytes fungi Epi R2-21 and Alt XHYN2 after 6 h, 6 d, and 15 d inoculation, respectively ($|Log_2FC| \geq 1$; $p$-adjust < 0.05).

| No. | gene ID | KO ID | Gene product | KEGG definition | Gene description | Log₂FC | | | | | |
|---|---|---|---|---|---|---|---|---|---|---|---|
| | | | | | | **R2-21_6h vs Con_6h** | **R2-21_6d vs Con_6d** | **R2-21_15d vs Con_15d** | **XHYN2_6h vs Con_6h** | **XHYN2_6d vs Con_6d** | **XHYN2_15d vs Con_15d** |
| 1 | *VIT_16s0100g00770* | K00660 | CHS | chalcone synthase [EC:2.3.1.74] | Stilbene synthase 3 | 3.48 | 5.65 | 7.20 | 5.17 | 6.48 | 6.54 |
| 2 | *VIT_16s0100g01140* | K00660 | CHS | chalcone synthase [EC:2.3.1.74] | Stilbene synthase 1 | 3.85 | 5.53 | 7.71 | 5.27 | 6.19 | 7.26 |
| 3 | *VIT_16s0100g00780* | K00660 | CHS | chalcone synthase [EC:2.3.1.74] | Stilbene synthase 5 | 2.44 | 4.78 | 6.91 | 3.61 | 5.24 | 6.86 |
| 4 | *VIT_16s0100g00750* | K00660 | CHS | chalcone synthase [EC:2.3.1.74] | Stilbene synthase 6 | 3.51 | 6.09 | 7.32 | 4.72 | 6.87 | 6.87 |
| 5 | *VIT_16s0100g01110* | K00660 | CHS | chalcone synthase [EC:2.3.1.74] | Stilbene synthase 1 | 4.37 | 5.17 | 7.83 | 5.75 | 5.75 | 7.39 |
| 6 | *VIT_16s0100g01120* | K00660 | CHS | chalcone synthase [EC:2.3.1.74] | Stilbene synthase 6 | 4.66 | 5.53 | 8.14 | 7.32 | 7.14 | 7.89 |
| 7 | *VIT_16s0100g01190* | K00660 | CHS | chalcone synthase [EC:2.3.1.74] | Stilbene synthase 1 | 3.24 | 5.33 | 7.01 | 4.76 | 6.22 | 6.55 |
| 8 | *VIT_16s0100g00830* | K00660 | CHS | chalcone synthase [EC:2.3.1.74] | Stilbene synthase 5 | 3.20 | 5.25 | 6.42 | 4.58 | 5.96 | 6.03 |
| 9 | *VIT_16s0100g01150* | K00660 | CHS | chalcone synthase [EC:2.3.1.74] | Stilbene synthase 6 | 3.89 | 5.66 | 8.31 | 5.54 | 6.58 | 8.01 |
| 10 | *VIT_16s0100g00910* | K00660 | CHS | chalcone synthase [EC:2.3.1.74] | Stilbene synthase 5 | 3.00 | 5.47 | 4.88 | 4.29 | 6.00 | 4.35 |
| 11 | *VIT_16s0100g01000* | K00660 | CHS | chalcone synthase [EC:2.3.1.74] | Stilbene synthase 4 | 2.39 | 5.40 | 3.55 | 3.44 | 6.06 | 2.96 |
| 12 | *VIT_16s0100g00990* | K00660 | CHS | chalcone synthase [EC:2.3.1.74] | Stilbene synthase 2 | 3.00 | 5.87 | 4.56 | 4.43 | 6.57 | 3.90 |
| 13 | *VIT_16s0100g01040* | K00660 | CHS | chalcone synthase [EC:2.3.1.74] | Stilbene synthase 4 | 2.86 | 5.19 | 4.83 | 4.12 | 6.05 | 4.51 |
| 14 | *VIT_16s0100g01010* | K00660 | CHS | chalcone synthase [EC:2.3.1.74] | Stilbene synthase 2 | 2.47 | 5.62 | 3.48 | 4.10 | 6.26 | 2.75 |
| 15 | *VIT_10s0042g00920* | K00660 | CHS | chalcone synthase [EC:2.3.1.74] | Stilbene synthase 1 | 2.49 | 4.34 | 1.69 | 4.22 | 5.65 | 1.50 |
| 16 | *VIT_10s0042g00840* | K00660 | CHS | chalcone synthase [EC:2.3.1.74] | Stilbene synthase 3 | 3.19 | 4.71 | 1.94 | 5.38 | 5.71 | 1.77 |
| 17 | *VIT_16s0100g00840* | K00660 | CHS | chalcone synthase [EC:2.3.1.74] | Stilbene synthase 4 | 2.51 | 4.84 | 2.47 | 3.69 | 5.51 | 1.90 |
| 18 | *VIT_16s0100g00940* | K00660 | CHS | chalcone synthase [EC:2.3.1.74] | Stilbene synthase 4 | 2.62 | 4.91 | 2.45 | 4.12 | 5.42 | 1.91 |
| 19 | *VIT_16s0100g00930* | K00660 | CHS | chalcone synthase [EC:2.3.1.74] | Stilbene synthase 4 | 2.28 | 4.86 | 2.19 | 4.17 | 5.48 | 1.72 |
| 20 | *VIT_16s0100g00920* | K00660 | CHS | chalcone synthase | Stilbene synthase 4 | 2.55 | 4.47 | 2.06 | 3.94 | 5.20 | 1.65 |

| | | | | | | | | | | |
|---|---|---|---|---|---|---|---|---|---|---|
| | | | | [EC:2.3.1.74] | | | | | | |
| 21 | *VIT_16s0100g01100* | K00660 | CHS | chalcone synthase [EC:2.3.1.74] | Stilbene synthase 1 | / | 5.09 | 9.82 | 6.26 | 6.32 | 9.31 |
| 22 | *VIT_16s0100g01070* | K00660 | CHS | chalcone synthase [EC:2.3.1.74] | Stilbene synthase 1 | / | 5.00 | 7.67 | 3.36 | 5.77 | 7.23 |
| 23 | *VIT_16s0100g00880* | K00660 | CHS | chalcone synthase [EC:2.3.1.74] | Stilbene synthase 4 | / | 5.54 | 4.06 | 3.76 | 6.26 | 3.54 |
| 24 | *VIT_16s0100g01170* | K00660 | CHS | chalcone synthase [EC:2.3.1.74] | Stilbene synthase 1 | / | 5.26 | 5.86 | 4.12 | 6.16 | 5.44 |
| 25 | *VIT_16s0100g01020* | K00660 | CHS | chalcone synthase [EC:2.3.1.74] | Stilbene synthase 4 | / | 5.67 | 3.85 | / | 6.35 | 3.33 |
| 26 | *VIT_16s0100g00850* | K00660 | CHS | chalcone synthase [EC:2.3.1.74] | Stilbene synthase 4 | / | 5.33 | 2.53 | / | 6.45 | 2.38 |
| 27 | *VIT_10s0042g00930* | K00660 | CHS | chalcone synthase [EC:2.3.1.74] | Stilbene synthase 1 | 2.57 | 3.96 | / | 3.73 | 4.75 | / |
| 28 | *VIT_10s0042g00910* | K00660 | CHS | chalcone synthase [EC:2.3.1.74] | Stilbene synthase 3 | / | 5.67 | 2.04 | 4.21 | 6.66 | / |
| 29 | *VIT_10s0042g00850* | K00660 | CHS | chalcone synthase [EC:2.3.1.74] | Stilbene synthase 2 | / | 3.91 | / | 5.69 | 5.34 | / |
| 30 | *VIT_10s0042g00880* | K00660 | CHS | chalcone synthase [EC:2.3.1.74] | Stilbene synthase 3 | / | 7.24 | / | / | 8.45 | / |
| 31 | *VIT_10s0042g00860* | K00660 | CHS | chalcone synthase [EC:2.3.1.74] | Stilbene synthase 4 | / | 4.75 | / | 4.54 | / | / |
| 32 | *VIT_05s0136g00260* | K00660 | CHS | chalcone synthase [EC:2.3.1.74] | Chalcone synthase 2 | / | / | 1.28 | / | / | / |
| 33 | *VIT_14s0068g00920* | K00660 | CHS | chalcone synthase [EC:2.3.1.74] | Chalcone synthase | -3.56 | / | 1.64 | -5.74 | -2.66 | / |
| 34 | *VIT_14s0068g00930* | K00660 | CHS | chalcone synthase [EC:2.3.1.74] | Chalcone synthase | -2.96 | / | / | -4.73 | / | / |
| 35 | *VIT_16s0100g01130* | K13232; K00660 | STS;CHS | stilbene synthase [EC:2.3.1.95];chalcone synthase [EC:2.3.1.74] | Stilbene synthase 1 | 3.07 | 5.68 | 7.20 | 4.15 | 6.51 | 6.77 |
| 36 | *VIT_10s0042g00890* | K13232; K00660 | STS;CHS | stilbene synthase [EC:2.3.1.95];chalcone synthase [EC:2.3.1.74] | Stilbene synthase 1 | 3.03 | 4.47 | 2.14 | 4.68 | 6.04 | 2.56 |
| 37 | *VIT_16s0100g00860* | K13232; K00660 | STS;CHS | stilbene synthase [EC:2.3.1.95];chalcone synthase [EC:2.3.1.74] | Stilbene synthase 4 | 2.68 | 4.88 | 2.74 | 4.07 | 5.37 | 2.27 |
| 38 | *VIT_10s0042g00870* | K13232; K00660 | STS;CHS | stilbene synthase [EC:2.3.1.95];chalcone synthase [EC:2.3.1.74] | Stilbene synthase 2 | 2.86 | 4.57 | 2.59 | 4.53 | 5.71 | 2.51 |
| 39 | *VIT_16s0100g00960* | K13232; K00660 | STS;CHS | stilbene synthase [EC:2.3.1.95];chalcone synthase [EC:2.3.1.74] | Stilbene synthase 4 | 2.59 | 5.67 | 3.61 | 3.84 | 6.24 | 2.97 |
| 40 | *VIT_16s0100g00950* | K13232; K00660 | STS;CHS | stilbene synthase [EC:2.3.1.95];chalcone synthase [EC:2.3.1.74] | Stilbene synthase 2 | 2.09 | 5.71 | 3.24 | 3.51 | 6.41 | 2.63 |

| | | | | | | | | | | | |
|---|---|---|---|---|---|---|---|---|---|---|---|
| 41 | *VIT_16s0100g00810* | K13232; K00660 | STS;CHS | stilbene synthase [EC:2.3.1.95];chalcone synthase [EC:2.3.1.74] | Stilbene synthase 4 | 2.41 | 5.54 | 3.81 | 3.83 | 6.30 | 3.16 |
| 42 | *VIT_06s0009g03110* | K13083 | CYP75A | flavonoid 3',5'-hydroxylase [EC:1.14.14.81] | Flavonoid 3',5'-hydroxylase 2 | / | 5.86 | 5.77 | / | 5.66 | / |
| 43 | *VIT_08s0007g05160* | K13083 | CYP75A | flavonoid 3',5'-hydroxylase [EC:1.14.14.81] | Flavonoid 3',5'-hydroxylase 2 | / | / | 2.91 | / | / | 2.56 |
| 44 | *VIT_06s0009g03040* | K13083 | CYP75A | flavonoid 3',5'-hydroxylase [EC:1.14.14.81] | Flavonoid 3',5'-hydroxylase 2 | / | 2.20 | / | / | / | / |
| 45 | *VIT_06s0009g02810* | K13083 | CYP75A | flavonoid 3',5'-hydroxylase [EC:1.14.14.81] | Flavonoid 3',5'-hydroxylase 2 | / | / | -3.94 | / | 2.61 | / |
| 46 | *VIT_06s0009g03010* | K13083 | CYP75A | flavonoid 3',5'-hydroxylase [EC:1.14.14.81] | Flavonoid 3',5'-hydroxylase 2 | / | -1.98 | -1.92 | / | -2.28 | -2.56 |
| 47 | *VIT_06s0009g02860* | K13083 | CYP75A | flavonoid 3',5'-hydroxylase [EC:1.14.14.81] | Flavonoid 3',5'-hydroxylase 2 | / | -2.34 | -2.50 | / | -2.98 | -2.21 |
| 48 | *VIT_06s0009g02840* | K13083 | CYP75A | flavonoid 3',5'-hydroxylase [EC:1.14.14.81] | Flavonoid 3',5'-hydroxylase 2 | / | -1.18 | / | / | / | -1.08 |
| 49 | *VIT_06s0009g02920* | K13083 | CYP75A | flavonoid 3',5'-hydroxylase [EC:1.14.14.81] | Flavonoid 3',5'-hydroxylase 2 | / | / | -2.07 | / | / | / |
| 50 | *VIT_06s0009g02830* | K13083 | CYP75A | flavonoid 3',5'-hydroxylase [EC:1.14.14.81] | Flavonoid 3',5'-hydroxylase | / | -2.19 | / | / | / | / |
| 51 | *VIT_06s0009g02970* | K13083 | CYP75A | flavonoid 3',5'-hydroxylase [EC:1.14.14.81] | Flavonoid 3',5'-hydroxylase 2 | / | -1.93 | / | / | -2.05 | / |
| 52 | *VIT_06s0009g02910* | K13083 | CYP75A | flavonoid 3',5'-hydroxylase [EC:1.14.14.81] | Flavonoid 3',5'-hydroxylase | / | -4.14 | / | / | -3.13 | -4.12 |
| 53 | *VIT_06s0009g03050* | K13083 | CYP75A | flavonoid 3',5'-hydroxylase [EC:1.14.14.81] | Flavonoid 3',5'-hydroxylase 2 | / | / | -4.81 | / | / | -5.01 |
| 54 | *VIT_11s0065g00350* | K00487 | CYP73A | trans-cinnamate 4-monooxygenase [EC:1.14.14.91] | Cytochrome P450 CYP73A100 | 2.79 | 4.36 | 5.66 | 4.04 | 5.22 | 5.61 |
| 55 | *VIT_11s0078g00290* | K00487 | CYP73A | trans-cinnamate 4-monooxygenase [EC:1.14.14.91] | Cytochrome P450 CYP73A100 | / | 4.38 | 7.05 | / | 4.52 | 7.07 |
| 56 | *VIT_06s0004g08150* | K00487 | CYP73A | trans-cinnamate 4-monooxygenase [EC:1.14.14.91] | Trans-cinnamate 4-monooxygenase | / | / | 1.10 | 2.27 | / | 1.27 |
| 57 | *VIT_18s0001g03430* | K05278 | FLS | flavonol synthase [EC:1.14.20.6] | Flavonol synthase/flavanone 3-hydroxylase | / | / | 1.38 | / | / | 1.29 |
| 58 | *VIT_18s0001g03510* | K05278 | FLS | flavonol synthase [EC:1.14.20.6] | Flavonol synthase/flavanone 3-hydroxylase | / | / | / | / | 3.03 | 3.73 |
| 59 | *VIT_18s0001g14310* | K00475 | F3H | naringenin 3-dioxygenase [EC:1.14.11.9] | Flavanone 3-dioxygenase | / | / | 3.59 | / | / | 3.68 |
| 60 | *VIT_04s0023g03370* | K00475 | F3H | naringenin 3-dioxygenase [EC:1.14.11.9] | Naringenin,2-oxoglutarate 3-dioxygenase | / | / | 1.08 | / | / | / |
| 61 | *VIT_13s0067g03820* | K01859 | CHI | chalcone isomerase | Chalcone--flavonone | 1.80 | 1.24 | 2.58 | 2.64 | 1.37 | 2.28 |

| | | | | | | | | | | | |
|---|---|---|---|---|---|---|---|---|---|---|---|
| | | | | [EC:5.5.1.6] | isomerase 2 | | | | | | |
| 62 | *VIT_07s0031g00350* | K00588 | CCoAOMT | caffeoyl-CoA O-methyltransferase [EC:2.1.1.104] | Caffeoyl-CoA O-methyltransferase | / | 2.58 | 3.00 | / | 2.65 | 3.22 |
| 63 | *VIT_03s0063g00140* | K00588 | CCoAOMT | caffeoyl-CoA O-methyltransferase [EC:2.1.1.104] | Caffeoyl-CoA O-methyltransferase | / | 2.61 | 1.95 | / | 2.20 | 1.92 |
| 64 | *VIT_12s0028g03110* | K00588 | CCoAOMT | caffeoyl-CoA O-methyltransferase [EC:2.1.1.104] | Caffeoyl-CoA O-methyltransferase | / | / | / | / | / | -1.25 |
| 65 | *VIT_11s0037g00440* | K13065 | HCT | shikimate O-hydroxycinnamoyltransferase [EC:2.3.1.133] | Shikimate O-hydroxycinnamoyltransferase | / | 1.36 | 2.11 | / | / | 2.27 |
| 66 | *VIT_09s0018g01190* | K13065 | HCT | shikimate O-hydroxycinnamoyltransferase [EC:2.3.1.133] | Shikimate O-hydroxycinnamoyltransferase | / | / | / | 2.09 | / | / |
| 67 | *VIT_11s0037g00570* | K13065 | HCT | shikimate O-hydroxycinnamoyltransferase [EC:2.3.1.133] | Spermidine hydroxycinnamoyl transferase | / | / | -1.22 | / | -1.11 | / |
| 68 | *VIT_11s0037g00580* | K13065 | HCT | shikimate O-hydroxycinnamoyltransferase [EC:2.3.1.133] | Spermidine hydroxycinnamoyl transferase | / | / | -1.12 | / | / | / |
| 69 | *VIT_06s0061g01430* | K13080 | C12RT1 | flavanone 7-O-glucoside 2"-O-beta-L-rhamnosyltransferase [EC:2.4.1.236] | Flavanone 7-O-glucoside 2'-O-beta-L-rhamnosyltransferase | / | / | 1.86 | / | / | 1.39 |
| 70 | *VIT_02s0025g04720* | K05277 | ANS | anthocyanidin synthase [EC:1.14.20.4] | Leucoanthocyanidin dioxygenase | / | / | 2.27 | / | / | 1.81 |
| 71 | *VIT_08s0040g00780* | K09754 | CYP98A, C3'H | 5-O-(4-coumaroyl)-D-quinate 3'-monooxygenase [EC:1.14.14.96] | Cytochrome P450 98A2 | 1.79 | / | / | 2.46 | / | / |
| 72 | *VIT_18s0001g12800* | K13082 | DFR | bifunctional dihydroflavonol 4-reductase/flavanone 4-reductase [EC:1.1.1.219 1.1.1.234] | Dihydroflavonol 4-reductase | / | / | / | / | -1.48 | / |
| 73 | *VIT_01s0011g02960* | K13081 | LAR | leucoanthocyanidin reductase [EC:1.17.1.3] | Leucoanthocyanidin reductase | / | -3.49 | / | / | / | / |
| 74 | *VIT_17s0000g07200* | K05280 | CYP75B1 | flavonoid 3'-monooxygenase [EC:1.14.14.82] | Flavonoid 3'-monooxygenase | / | / | / | / | -1.32 | / |

**Table S8.** DEGs related to the stilbenoid, diarylheptanoid and gingerol biosynthesis pathway of the tissue cultured seedlings of 'Rose Honey' in response to endophytes fungi Epi R2-21 and Alt XHYN2 after 6 h, 6 d, and 15 d inoculation, respectively ($|Log_2FC| \geq 1$; *p*-adjust < 0.05).

| No. | gene ID | KO ID | Gene product | KEGG definition | Gene description | $Log_2FC$ | | | | | |
|---|---|---|---|---|---|---|---|---|---|---|---|
| | | | | | | **R2-21_ 6h vs Con_6h** | **R2-21_ 6d vs Con_6d** | **R2-21_15 d vs Con_15d** | **XHYN2 _6h vs Con_6h** | **XHYN2 _6d vs Con_6d** | **XHYN2_ 15d vs Con_15d** |
| 1 | *VIT_16s0100g01130* | K13232; K00660 | STS;CHS | stilbene synthase [EC:2.3.1.95];chalcone synthase [EC:2.3.1.74] | Stilbene synthase 1 | 3.07 | 4.15 | 6.77 | 6.51 | 5.68 | 7.20 |
| 2 | *VIT_16s0100g00810* | K13232; K00660 | STS;CHS | stilbene synthase [EC:2.3.1.95];chalcone synthase [EC:2.3.1.74] | Stilbene synthase 4 | 2.41 | 3.83 | 3.16 | 6.30 | 5.54 | 3.81 |
| 3 | *VIT_16s0100g00960* | K13232; K00660 | STS;CHS | stilbene synthase [EC:2.3.1.95];chalcone synthase [EC:2.3.1.74] | Stilbene synthase 4 | 2.59 | 3.84 | 2.97 | 6.24 | 5.67 | 3.61 |
| 4 | *VIT_16s0100g00860* | K13232; K00660 | STS;CHS | stilbene synthase [EC:2.3.1.95];chalcone synthase [EC:2.3.1.74] | Stilbene synthase 4 | 2.68 | 4.07 | 2.27 | 5.37 | 4.88 | 2.74 |
| 5 | *VIT_10s0042g00870* | K13232; K00660 | STS;CHS | stilbene synthase [EC:2.3.1.95];chalcone synthase [EC:2.3.1.74] | Stilbene synthase 2 | 2.86 | 4.53 | 2.51 | 5.71 | 4.57 | 2.59 |
| 6 | *VIT_16s0100g00950* | K13232; K00660 | STS;CHS | stilbene synthase [EC:2.3.1.95];chalcone synthase [EC:2.3.1.74] | Stilbene synthase 2 | 2.09 | 3.51 | 2.63 | 6.41 | 5.71 | 3.24 |
| 7 | *VIT_10s0042g00890* | K13232; K00660 | STS;CHS | stilbene synthase [EC:2.3.1.95];chalcone synthase [EC:2.3.1.74] | Stilbene synthase 1 | / | / | 2.56 | 6.04 | 4.47 | 2.14 |
| 8 | *VIT_16s0100g01160* | K13232 | STS | stilbene synthase [EC:2.3.1.95] | Stilbene synthase 1 | 3.62 | 5.20 | 7.14 | 5.31 | 4.60 | 7.54 |
| 9 | *VIT_16s0100g00900* | K13232 | STS | stilbene synthase [EC:2.3.1.95] | Stilbene synthase 4 | 2.50 | 4.05 | 3.68 | 6.44 | 5.70 | 4.24 |
| 10 | *VIT_16s0100g01030* | K13232 | STS | stilbene synthase [EC:2.3.1.95] | Stilbene synthase 3 | 2.79 | 3.98 | 4.33 | 6.01 | 5.17 | 4.51 |
| 11 | *VIT_16s0100g01200* | K13232 | STS | stilbene synthase [EC:2.3.1.95] | Stilbene synthase 6 | / | 3.76 | 7.25 | 5.92 | 5.01 | 7.59 |
| 12 | *VIT_10s0003g00480* | K16040 | ROMT | trans-resveratrol di-O-methyltransferase [EC:2.1.1.240] | Trans-resveratrol di-O-methyltransferase | / | / | 1.81 | 1.50 | 1.43 | 1.81 |
| 13 | *VIT_12s0028g01940* | K16040 | ROMT | trans-resveratrol di-O-methyltransferase [EC:2.1.1.240] | Trans-resveratrol di-O-methyltransferase | / | / | 3.74 | 4.40 | 4.58 | 3.76 |
| 14 | *VIT_12s0028g02800* | K16040 | ROMT | trans-resveratrol di-O-methyltransferase [EC:2.1.1.240] | Trans-resveratrol di-O-methyltransferase | / | / | 8.70 | 7.76 | 6.91 | 9.55 |

| | | | | | | | | | | | |
|---|---|---|---|---|---|---|---|---|---|---|---|
| 15 | *VIT_12s0028g02700* | K16040 | ROMT | trans-resveratrol di-O-methyltransferase [EC:2.1.1.240] | Trans-resveratrol di-O-methyltransferase | / | / | 10.06 | 7.65 | 7.33 | 10.97 |
| 16 | *VIT_12s0028g02740* | K16040 | ROMT | trans-resveratrol di-O-methyltransferase [EC:2.1.1.240] | Trans-resveratrol di-O-methyltransferase | / | / | 7.56 | / | 10.15 | 9.93 |
| 17 | *VIT_12s0028g02760* | K16040 | ROMT | trans-resveratrol di-O-methyltransferase [EC:2.1.1.240] | Trans-resveratrol di-O-methyltransferase | / | / | 5.25 | / | / | 4.97 |
| 18 | *VIT_12s0028g02710* | K16040 | ROMT | trans-resveratrol di-O-methyltransferase [EC:2.1.1.240] | Trans-resveratrol di-O-methyltransferase | 5.34 | / | 4.78 | / | / | / |
| 19 | *VIT_12s0028g02830* | K16040 | ROMT | trans-resveratrol di-O-methyltransferase [EC:2.1.1.240] | Trans-resveratrol di-O-methyltransferase | / | / | 5.35 | / | / | / |
| 20 | *VIT_12s0028g02840* | K16040 | ROMT | trans-resveratrol di-O-methyltransferase [EC:2.1.1.240] | Trans-resveratrol di-O-methyltransferase | / | / | / | / | 3.49 | / |
| 21 | *VIT_12s0028g02890* | K16040 | ROMT | trans-resveratrol di-O-methyltransferase [EC:2.1.1.240] | Trans-resveratrol di-O-methyltransferase | / | / | / | / | / | 1.36 |
| 22 | *VIT_10s0003g00460* | K16040 | ROMT | trans-resveratrol di-O-methyltransferase [EC:2.1.1.240] | Trans-resveratrol di-O-methyltransferase | / | / | 4.64 | / | / | / |
| 23 | *VIT_12s0028g01880* | K16040 | ROMT | trans-resveratrol di-O-methyltransferase [EC:2.1.1.240] | Trans-resveratrol di-O-methyltransferase | / | / | / | 2.70 | / | / |
| 24 | *VIT_12s0028g02810* | K16040 | ROMT | trans-resveratrol di-O-methyltransferase [EC:2.1.1.240] | Trans-resveratrol di-O-methyltransferase | / | / | / | / | / | 5.68 |
| 25 | *VIT_12s0028g02850* | K16040 | ROMT | trans-resveratrol di-O-methyltransferase [EC:2.1.1.240] | Trans-resveratrol di-O-methyltransferase | / | / | -7.64 | / | / | / |
| 26 | *VIT_15s0045g01490* | K16040 | ROMT | trans-resveratrol di-O-methyltransferase [EC:2.1.1.240] | Trans-resveratrol di-O-methyltransferase | / | / | / | / | / | -4.39 |
| 27 | *VIT_10s0003g00440* | K16040; K13262 | ROMT;7-IOMT | trans-resveratrol di-O-methyltransferase [EC:2.1.1.240];isoflavone-7-O-methyltransferase [EC:2.1.1.150] | Trans-resveratrol di-O-methyltransferase | / | / | 5.19 | / | / | / |
| 28 | *VIT_11s0037g00440* | K13065 | HCT | shikimate O-hydroxycinnamoyltransferase [EC:2.3.1.133] | Shikimate O-hydroxycinnamoyltransferase | / | 1.38 | 2.15 | / | / | 2.28 |
| 29 | *VIT_09s0018g01190* | K13065 | HCT | shikimate O-hydroxycinnamoyltransferase [EC:2.3.1.133] | Shikimate O-hydroxycinnamoyltransferase | / | 2.09 | / | / | / | / |

| | | | | | | | | | | | |
|---|---|---|---|---|---|---|---|---|---|---|---|
| 30 | *VIT_11s0037g00570* | K13065 | HCT | shikimate O-hydroxycinnamoyltransferase [EC:2.3.1.133] | Shikimate O-hydroxycinnamoyltransferase | / | / | / | -1.11 | / | -1.22 |
| 31 | *VIT_11s0037g00580* | K13065 | HCT | shikimate O-hydroxycinnamoyltransferase [EC:2.3.1.133] | Shikimate O-hydroxycinnamoyltransferase | / | / | -1.12 | / | / | / |
| 32 | *VIT_07s0031g00350* | K00588 | CCoAOMT | caffeoyl-CoA O-methyltransferase [EC:2.1.1.104] | Caffeoyl-CoA O-methyltransferase | / | / | 3.22 | 2.65 | 2.58 | 3.00 |
| 33 | *VIT_03s0063g00140* | K00588 | CCoAOMT | caffeoyl-CoA O-methyltransferase [EC:2.1.1.104] | Caffeoyl-CoA O-methyltransferase | / | / | 1.92 | 2.20 | 2.61 | 1.95 |
| 34 | *VIT_12s0028g03110* | K00588 | CCoAOMT | caffeoyl-CoA O-methyltransferase [EC:2.1.1.104] | Caffeoyl-CoA O-methyltransferase | / | / | -1.25 | / | / | / |
| 35 | *VIT_11s0065g00350* | K00487 | CYP73A | trans-cinnamate 4-monooxygenase [EC:1.14.14.91] | Cytochrome P450 CYP73A100 | 2.79 | 4.04 | 5.61 | 5.22 | 4.36 | 5.66 |
| 36 | *VIT_11s0078g00290* | K00487 | CYP73A | trans-cinnamate 4-monooxygenase [EC:1.14.14.91] | Cytochrome P450 CYP73A100 | / | / | 7.07 | 4.52 | 4.38 | 7.05 |
| 37 | *VIT_06s0004g08150* | K00487 | CYP73A | trans-cinnamate 4-monooxygenase [EC:1.14.14.91] | Trans-cinnamate 4-monooxygenase | / | 2.27 | 1.27 | / | / | 1.10 |
| 38 | *VIT_08s0040g00780* | K09754 | CYP98A, C3'H | 5-O-(4-coumaroyl)-D-quinate 3'-monooxygenase [EC:1.14.14.96] | Cytochrome P450 98A2 | 1.79 | / | / | 2.46 | / | / |
