# Supplementary figures and images for: Cultivation of Fungal Endophytes with Tissue Culture Grapevine Seedlings Reprograms Metabolism by Triggering Defence Responses
Source: Metabolites. 2024 Jul 24;14(8):402. doi: 10.3390/metabo14080402 (PMC11356313; doi:10.3390/metabo14080402)

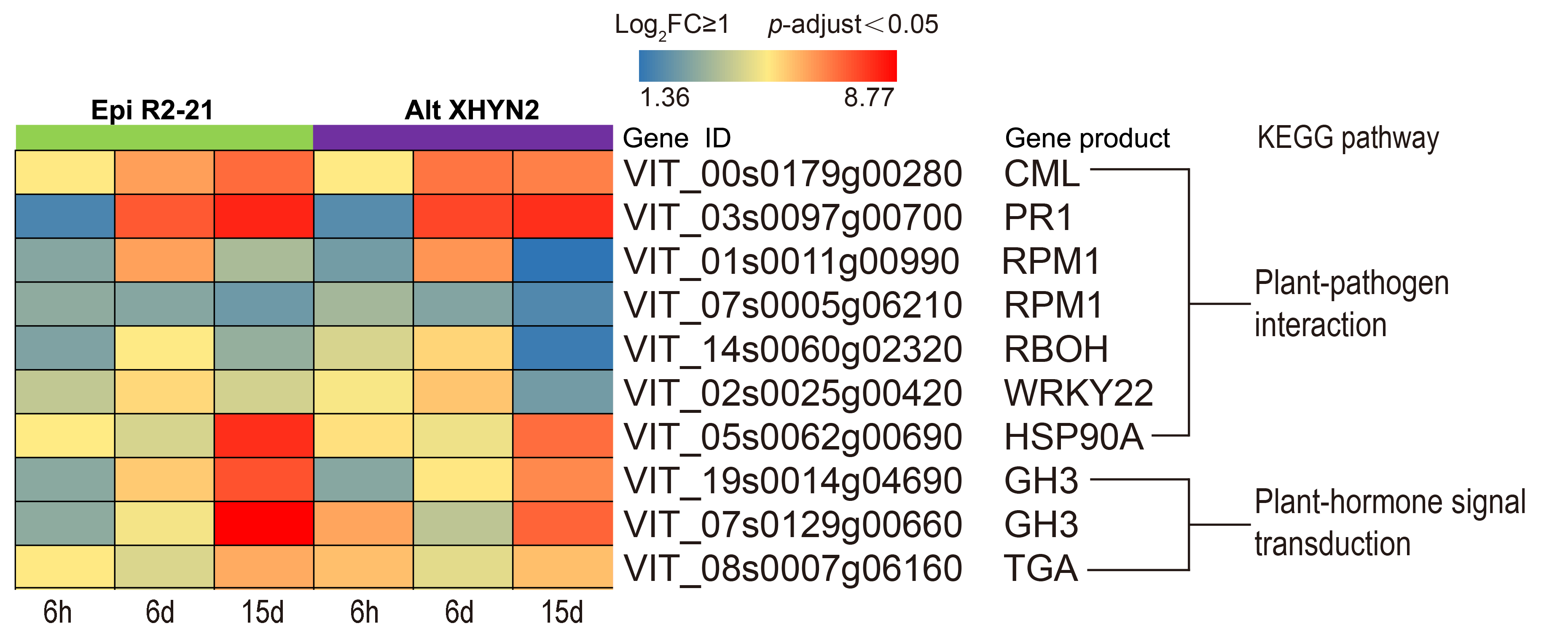

Supplement: Supplementary file 1 [file metabolites-14-00402-s001.zip › Figure S3.tif]

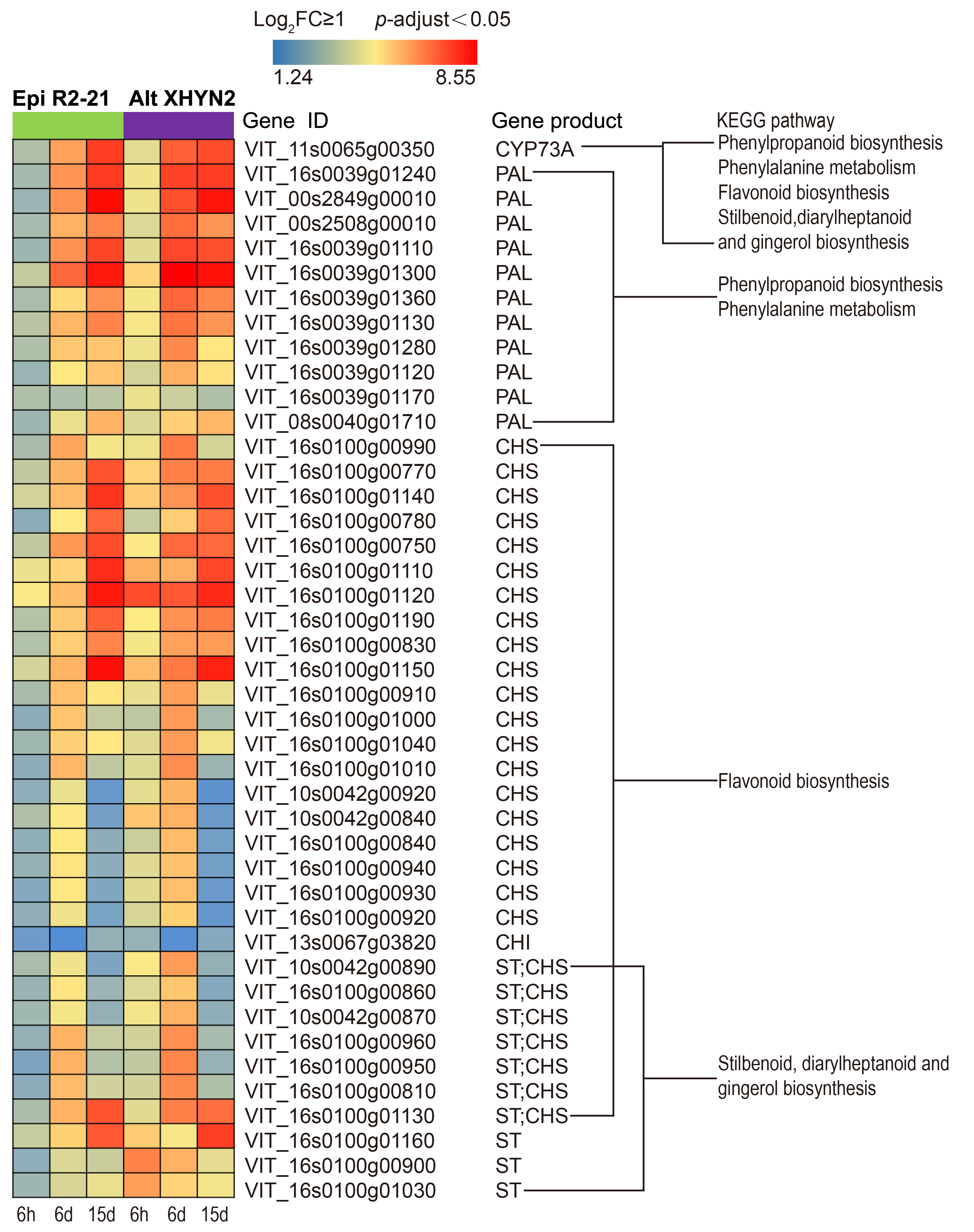

Supplement: Supplementary file 1 [file metabolites-14-00402-s001.zip › Figure S4.tif]
